# Identification, occurrence and prevention of aspartimide-related byproducts in chemical protein synthesis†

**DOI:** 10.1039/d5sc03824c

**Published:** 2025-07-08

**Authors:** El Hadji Cisse, Vincent Aucagne

**Affiliations:** a Centre de Biophysique Moléculaire, CNRS UPR 4301 Rue Charles Sadron 45071 Orléans Cedex 2 France vincent.aucagne@cnrs-orleans.fr

## Abstract

Formation of a five-membered ring aspartimide through the attack of a backbone amide to the side chain of aspartate and asparagine residues is a long-known side-reaction in solid phase peptide synthesis, and is also associated with *in vivo* protein ageing and instability of purified proteins. Conversely, its possible occurrence during chemical ligation-based protein synthesis, in particular when using the gold-standard reaction NCL (native chemical ligation), is dubious. We herein report a systematic study which demonstrates that the prevalence of this side-reaction may have been overlooked, due to the difficulty to identify it through standard HPLC analytical methods, but also the *in situ* conversion of aspartimide into other byproducts, having the same molecular mass as the parent aspartate residue. We show that the formation of aspartimide and derived byproducts can be limited by adopting “good NCL practices”, which involve restricting the ligation temperature and reaction times, as well as replacing the commonly used phosphate buffer with HEPES. However, the efficiency of such precautions is expected to vary considerably depending on the sequence of the target protein, and the amount of byproducts is expected to grow with the length of the target protein, as a result of the number of NCL reactions and potential aspartimide hotspots. To overcome such limitations, we developed a novel straightforward and potentially generally applicable methodology based on the temporary protection of the backbone nitrogen by a 2-(4-aminobutanoyloxy)-4-methoxybenzyl (GABA-Hmb) group. This strategy was validated by the byproduct-free synthesis of SUMO-2 and a SUMOylated peptide mimic.

## Abstract 2

Aspartimide formation is an overlooked side-reaction occurring during chemical ligation-based protein synthesis. We developed a straightforward and potentially generally applicable methodology to prevent it, based on a backbone nitrogen protection.

## Graphical abstract

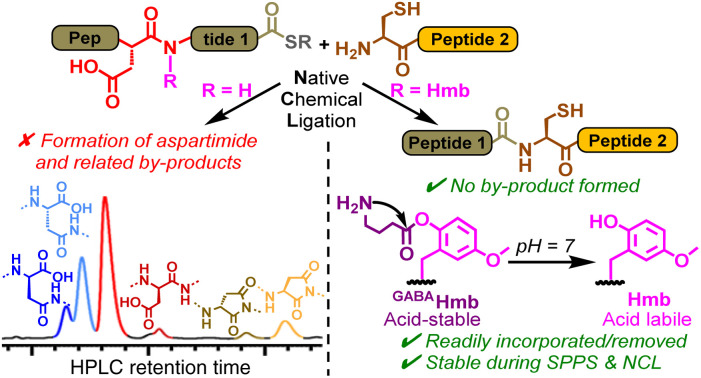

## Introduction

**Scheme 1:**
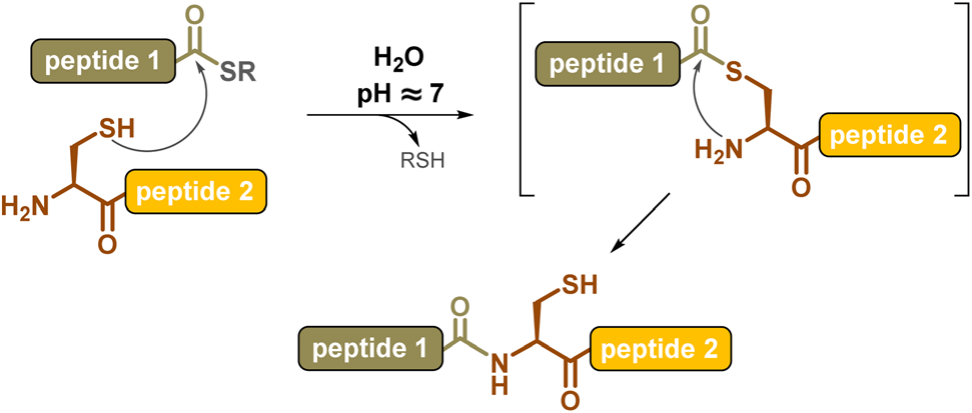
Native Chemical Ligation (NCL).

Chemical synthesis offers an effective alternative to access proteins that are difficult or impossible to produce through biotechnological means, such as ones with post-translational modifications (PTM) or “mirror-image” d-proteins, respectively. The invention of the chemical ligation concept^1^ in the early 1990s revolutionized this field, introducing proteins into the realm of synthetic organic chemistry. The chemoselective coupling of unprotected peptide segments equipped with mutually reactive groups largely outperformed the limits of solid phase peptide synthesis^2^ (SPPS), increasing the length of polypeptide chains that can be assembled in a controlled fashion from a few dozen amino acids to several hundred, and ultimately leading to correctly folded, high-purity proteins. The key breakthrough in the field was the introduction in 1994, by Kent and co-workers, of the native chemical ligation^3^ (NCL) reaction, consisting of the coupling of a peptide C-terminal thioester segment with a second segment bearing an N-terminal cysteine. The NCL reaction involves a reversible *trans*-thioesterification followed by spontaneous intramolecular *S*-to-*N*-acyl shift (Scheme 1), and can be carried out under mild aqueous conditions, compatible with a large variety of protein segments.

Despite other amide-forming ligation reactions^4–7^ being actively pursued, NCL remains, almost thirty years after its invention, the most commonly used reaction, and over a thousand different proteins have been obtained through this method.^8,9^ Recent impressive achievements include the synthesis of a 53 kDa tetra-ubiquitinylated α-myoglobin^10^ composed of 472 amino acids (AA), or a 775 AA mirror-image split Pfu DNA-polymerase,^11^ through the ligation of numerous segments.

Not surprisingly, the development of methodologies aimed to broaden the applicability of NCL is an extremely active field of research. Novel strategies include successive ligation reactions without isolation of intermediate products, such as one-pot^12^ or solid-supported approaches,^13^ methods aimed at simplifying the access to C-terminal thioesters through the widely adopted Fmoc protecting group-based SPPS,^8*b*,14^ incorporation of temporary solubilizing tags for handling insoluble or aggregation-prone segments,^15^ and strategies aimed at overcoming the need for a cysteine residue at a retrosynthetic ligation disconnection.^16^ Addressing side reactions occurring during NCL is another key point. Side reactions leading to the loss of reactivity of a segment, for example thioester hydrolysis, can be overcame through application of appropriate purification protocols,^17^ while side reactions involving the side chain of an amino acid residue can prove particularly problematic and compromise the purity of the chemically-synthesized protein. A typical example is oxidation of the methionine side chain, during the acidic cleavage and deprotection step of SPPS, as well as during further purifications and handling steps, which is prevented through its replacement by an isologous norleucine residue.^18^ Another is partial epimerization at the C-terminal residue, as recently pointed out by Melnyk, Agouridas and co-workers for serine thioesters,^19^ which could be an overlooked problem considering the difficulty to discriminate between a single d- or l-AA within a long polypeptide chain through standard HPLC and MS-based analytical methods.

In this context, the possible formation of an aspartimide (3-amino-*N*-alkyl-succinimide, Asi) five-membered ring, from an aspartate (Asp)^20^ or asparagine (Asn)^21^ residue during NCL drew our attention. Base-mediated Asi formation through the attack of a backbone amide on the ester-protected side chain of an Asp has been one of the most prominent long-standing problems in Fmoc-SPPS.^22,23^ Spontaneous Asi formation at physiological pH from either an Asp or Asn residue is also involved in the ageing of proteins *in vivo*^24^ and their stability *in vitro*.^25^ Additionally, aspartimide has been identified more recently as an evolution-selected PTM.^26^

Reports of aspartimide formation in the course of chemical ligation-mediated protein assembly have been extremely scarce. An early 2003 work on the NCL-based synthesis of the 166 AA protein H-RAS did report a compound showing a lower mass than expected (M-18 ± 2 Da) as the sole product.^27^ This could be attributed to aspartimide formation from an Asp (M-18, loss of a water molecule) or Asn (M-17, loss of ammonia) residue. Asi formation during an NCL reaction has only been suspected in four others reports to date.^20,21^ In only one case additional evidence, other than mass spectrometry, supported the aspartimide hypothesis: replacement of the Asp residue by a Glu abolished the M-18 byproduct observed during the NCL-mediated cyclization of a small peptide, sunflower trypsin inhibitor-1.^20*a*^

Strikingly, two of the above-cited reports, by Melnyk's lab^20*b*,*c*^ and ourselves,^20*d*^ concern the same protein, human SUMO-2, obtained in both cases through the ligation of two segments at an identical retrosynthetic disconnection at the sole cysteine residue (Cys48).^28^ The present report details a systematic evaluation of the propensity of Asi formation under NCL conditions: an overlooked and probably frequent side reaction whose occurrence remains uncharted because it forms byproducts that are not readily distinguishable from the target synthetic proteins by means of standard analytical methods. We propose a potentially generally-applicable methodology dedicated to completely abolish the formation of Asi and Asi-derived byproducts during NCL or other ligation reactions, and apply it to the aspartimide-free synthesis of SUMO-2 and derivatives.

## Results and discussion

### SUMO-2

Discovered in 1995,^29^ the Small Ubiquitin-like Modifier (SUMO) protein family belong to the ubiquitin-like category,^30^ a class of proteins that exhibit weak sequence similarity but have a three-dimensional structure strongly analogous to ubiquitin. To date five different paralogs, referred to as SUMO-1 to 5, have been identified in humans.^31^ SUMOylation is a PTM in which the C-terminus of the SUMO is attached to the lysine side chain of a target protein *via* an isopeptide bond. Dysregulation of SUMOylation is associated with many diseases.^31,32^ However, the physiological and pathological roles of SUMOylation remains to be fully elucidated, and the development of dedicated chemical biological tools is essential to improve our understanding of the underlying mechanisms.

### Early hints on aspartimide (Asi) formation during previous chemical synthesis of SUMO-2

**Scheme 2:**
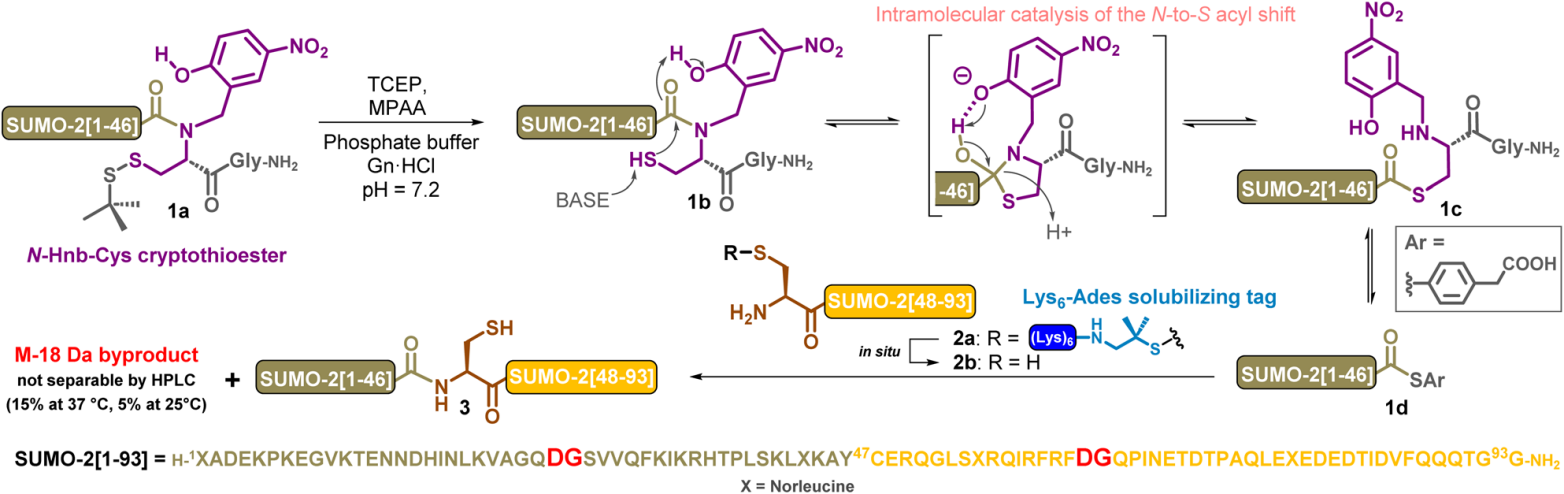
Previous work: synthesis of SUMO-2 (3) by NCL, showing the formation of a byproduct with a 18 Da mass loss, *N*-Hnb-Cys: *N*-(2-hydroxy-4-nitrobenzyl)cysteine and Ades: 2-amino-1,1-dimethyl-1-sulfanyl.

In our previous study, we synthesized SUMO-2 through the ligation of segments 1 and 2a (Scheme 2).^20*d*^ The SUMO-2[1–47] segment 1 was equipped at its C-terminus with the crypto-thioester device *N*-(2-hydroxy-4-nitrobenzyl)cysteine (*N*-Hnb-Cys), which has the ability to form a thioester *in situ* under NCL condition. This occurs through a spontaneous *N*-to-*S* acyl shift which is considerably accelerated by an intramolecular catalysis mediated by the phenol group.^33,34^ By itself, the SUMO-2[48–93] segment 2b is poorly soluble and shows a propensity for aggregation and an unusual HPLC behaviour,^35^ hampering its handling and purification. To solve this problem, we developed a dedicated strategy based on the introduction of a temporary solubilizing tag. (Lys)_6_-Ades (Ades = 2-amino-1,1-dimethyl-1-sulfanyl) is linked to the N-terminal cysteine by a disulfide linkage. The resulting segment 2a incorporates six additional hydrophilic lysine residues, which dramatically enhanced its aqueous solubility. NCL is usually conducted under reducing conditions to prevent formation of disulfides, typically using large excesses of tris-carboxyethylphosphine (TCEP) and 4-mercaptophenylacetic acid (MPAA),^36^ which also acts as a catalyst by forming *in situ* a highly reactive aryl thioester through a *trans*-thioesterification. Under such conditions, the (Lys)_6_-Ades tag of 2a is cleaved within few minutes, yielding an NCL-reactive segment (2b) which remains soluble: the ligation buffer contains 6 M guanidine hydrochloride (Gn·HCl), a denaturing medium extensively used for NCL to improve peptide solubility and avoid the partial folding of segments that could bury the reactive sites. Overnight incubation at 37 °C led to the obtainment of the desired protein 3. However, we also observed the formation of ≈15% of a byproduct showing an 18 Da mass decrease that could not be separated by HPLC, and could be attributed to aspartimide formation from an Asp residue. This was consistent with Melnyk's observations when synthesizing SUMO-2 using a bis-sulfanylethyl (SEA) *N*-to-*S* shift-based crypto-thioester,^37^ suggesting that formation of this byproduct is inherent to the target protein, and not to the method used to perform the NCL. In accordance with Melnyk's findings,^20*b*,*c*^ the amount of M-18 byproduct could be decreased to ≈5% by conducting the reaction at room temperature for 12 h.

### Characterization and rationalization of Asi byproducts on SUMO-2-based models

In the present work, we sought to characterize the putative aspartimide byproduct and to decipher the reasons for its formation. Besides environmental effects^25*a*,38^ (buffer, temperature *etc.*), numerous studies have highlighted the strong impact of the intrinsic properties of the peptide or protein. The local conformation, and the three-dimensional structure of the protein have a substantial influence on its propensity for Asi formation.^39^ The sequence surrounding the Asn/Asp is recognized to play a prominent role, and the Asp–Gly (DG) and Asn–Gly (NG) dipeptide motifs represent, by far, the main hotspots.^25,40^ The occurrence of DG and NG motifs has been estimated to an average 1.2 and 1.5 units per protein, respectively.^8*b*^ Given that over 1000 different proteins have been synthesized using NCL,^8*b*,9^ it is reasonable to assume that several hundred amongst them contain these motifs. However, to the best of our knowledge, only 5 articles have mentioned or suspected aspartimide formation during ligation reactions. This could mean that either only a very limited number of specific peptide sequences are likely to form aspartimides in the context of NCL, or that the detection of Asi and Asi-derived byproducts has been hampered by the analytical challenge it represents. Following the first hypothesis, and considering that, among these five examples, two articles concern human SUMO-2,^20*c*,*d*^ it seemed likely that this protein bears such a specific sequence with an exacerbated propension for aspartimide formation. SUMO-2 contains a total of 7 aspartate residues, including two in a DG motif (Asp27 and Asp64). We hypothesized that one of these two residues is likely to be involved, in either the context of the individual segments or in the full-length polypeptide chain.

**Scheme 3:**
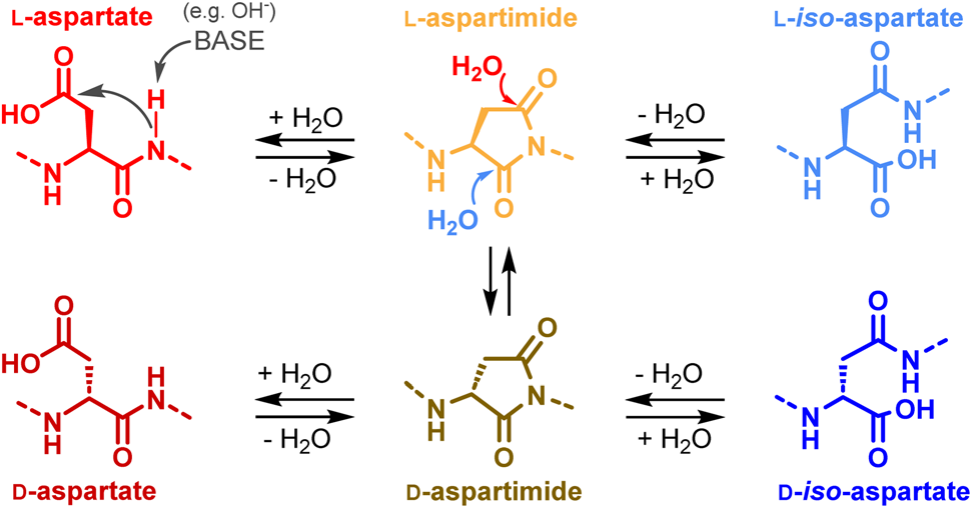
Aspartimide and aspartimide-derived byproducts.

**Scheme 4:**
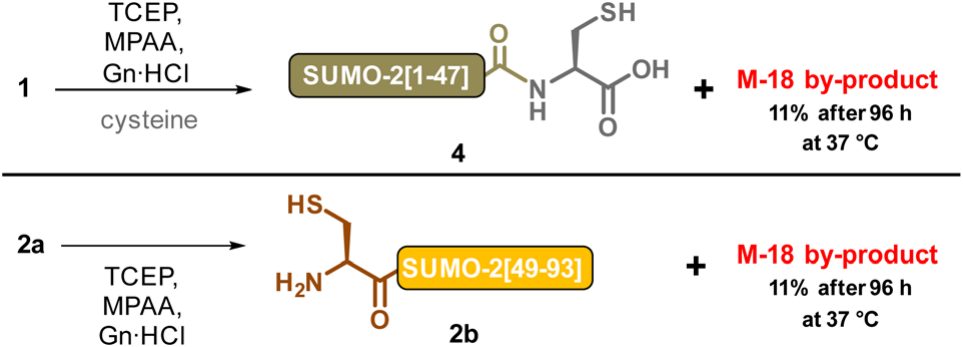
Individual incubation of the two SUMO-2 segments under NCL condition: 100 mM MPAA, 50 mM TCEP, 6 M Gu HCl, 200 mM sodium phosphate, apparent pH = 6.5, corrected pH^42^ = 7.2.

We first investigated whether SUMO-2 had an intrinsic capacity to form an aspartimide, even under non-denaturing conditions. We were surprised to see a decrease in the amount of the M-18 byproduct after incubating a sample of the protein in phosphate buffer saline (PBS, pH 7.4) at 37 °C for 2 days, (ESI, p. S6†). Aspartimide is known to be susceptible to nucleophilic attack by water leading to the formation of Asp and isoAsp (Scheme 3),^25*a*^ the latter being often preferred (average ratio 1 : 3). In addition to hydrolysis, Asi can also epimerize due to increased acidity of the Cα hydrogen.^41^ This leads to the formation of d-Asi, which will in turn be able to generate d-Asp and d-isoAsp (Scheme 3). The decrease we observed in the amount of the M-18 byproduct can thus likely be attributed to Asi hydrolysis under the incubation conditions. This observation did not rule out our hypothesis of a particularly Asi formation sensitive Asp residue in SUMO-2, and we therefore conducted experiments to determine the culpable residue. Taking advantage that the two DG motifs of SUMO-2 are located in different synthetic segments, we separately incubated 1 and 2a under NCL conditions (Scheme 4). Contrary to our expectations, we found that the two segments both led to the formation of M-18 byproducts, and with comparable kinetics: approximately 11% after 96 h at 37 °C for each of the two segments (ESI, p. S6–S8†). If the hypothesis that only a few specific sequences, amongst ones containing a DG or NG motif, are genuinely predisposed to aspartimide formation under NCL conditions is valid, it would seem very improbable that SUMO-2 contains two such sequences. The M-18 Da mass observed during the analysis of the full-length NCL product 3 is thus likely a mixture of the two aspartimide formed from both Asp residues at positions 26 and 63.

Taken collectively, our results suggest that the formation of aspartimide, assumed to be the M-18 Da products, could be an underestimated side reaction frequently occurring during NCL. Given that the separation by standard HPLC means of the target protein from Asi, but also from all the Asi-derived byproducts (*e.g.* isoAsp, d-Asp, d-isoAsp, and d-Asi), is not expected in the case of a long polypeptide chain, and that three of the byproducts are isobaric to the target (identical molecular mass), this raises an important concern that needs to be addressed.

### Model studies of formation of Asi and Asi-derived byproducts during NCL

To scrutinize the formation of Asi and Asi-derived byproducts during NCL, we concentrated on the model heptapeptide 5 encompassing the SUMO-2 [61–66] sequence with a central DG motif (Arg–Phe–Asp–Gly–Gln–Pro), and incorporating an extra C-terminal tryptophan residue for UV spectrophotometry quantification purposes. In addition to 5 we synthesized five analytical standards (6–10) corresponding to Asi formation (6), as well as related byproducts that could potentially be formed from 6 under NCL conditions. Peptides 7, 8 and 10 (l-isoAsp, d-isoAsp and d-Asp, respectively) were obtained by Fmoc-SPPS using commercially available building blocks: Fmoc-Asp-O*t*Bu, Fmoc-d-Asp(O*t*Bu)-OH and Fmoc-d-Asp(O*t*Bu)-OH, respectively. During the synthesis of l/d-isoAsp-containing peptides 7 and 10, we were able to obtain l/d aspartimide-containing peptides 6 and 9 respectively, formed during the SPPS (ESI, p. S8–S17†). We prepared an equimolar mixture of peptides 5–10 and optimized HPLC conditions for their analytical separation (Fig. S15†), the various peaks could then be distinguished by injecting each reference separately and comparing the retention times obtained. This separation is feasible for such small segments in strong contrast from what is expected with much longer segments typically employed for NCL-based chemical protein synthesis-

Having a suitable analytical tool in hand, we first examined the stability of the aspartimide peptide 6. Confirming the results observed with full length SUMO-2, incubation of 6 in PBS for 48 h at 37 °C led to >98% conversion into a mixture of peptides 5 and 7–10 (Table. S1† p. S18), with the isoAsp peptide 7 being the major compound (67%), having the same molecular mass as the parent Asp peptide 5. Similarly, incubation under NCL conditions (6 M Gn·HCl, 100 mM MPAA, 50 mM TCEP and 200 mM sodium phosphate, corrected pH^42^ = 7.2) for 16 h at 37 °C also led to the formation of significant amounts of the same products, albeit at lower rates, including 12% of epimerized forms (8–10) and 16% of isoAsp 7. This clearly indicates that, if formed during NCL, an aspartimide can further be converted into other byproducts, including d- and iso-forms undistinguishable by simple MS and HPLC analysis.

**Fig. 1:**
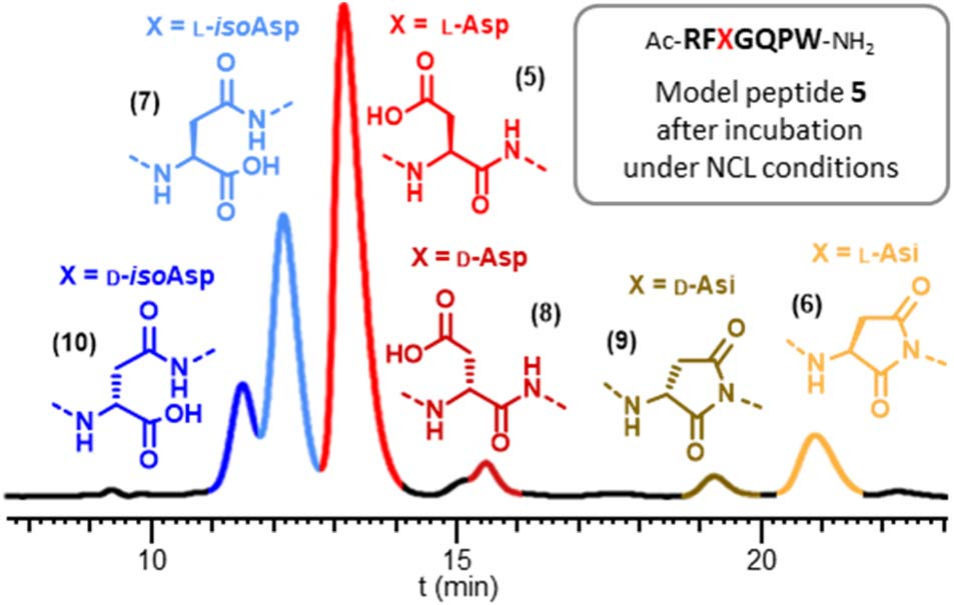
Typical RP-HPLC monitoring of the formation of aspartimide and derived byproducts from the DG motif-containing model peptide 5 upon incubation under forcing NCL conditions (100 mM MPAA, 50 mM TCEP, 6 M Gn·HCl, 200 mM sodium phosphate, apparent pH = 6.5, corrected pH^42^ = 7.2, 50 °C for 192 h (8 days)).

**Fig. 2:**
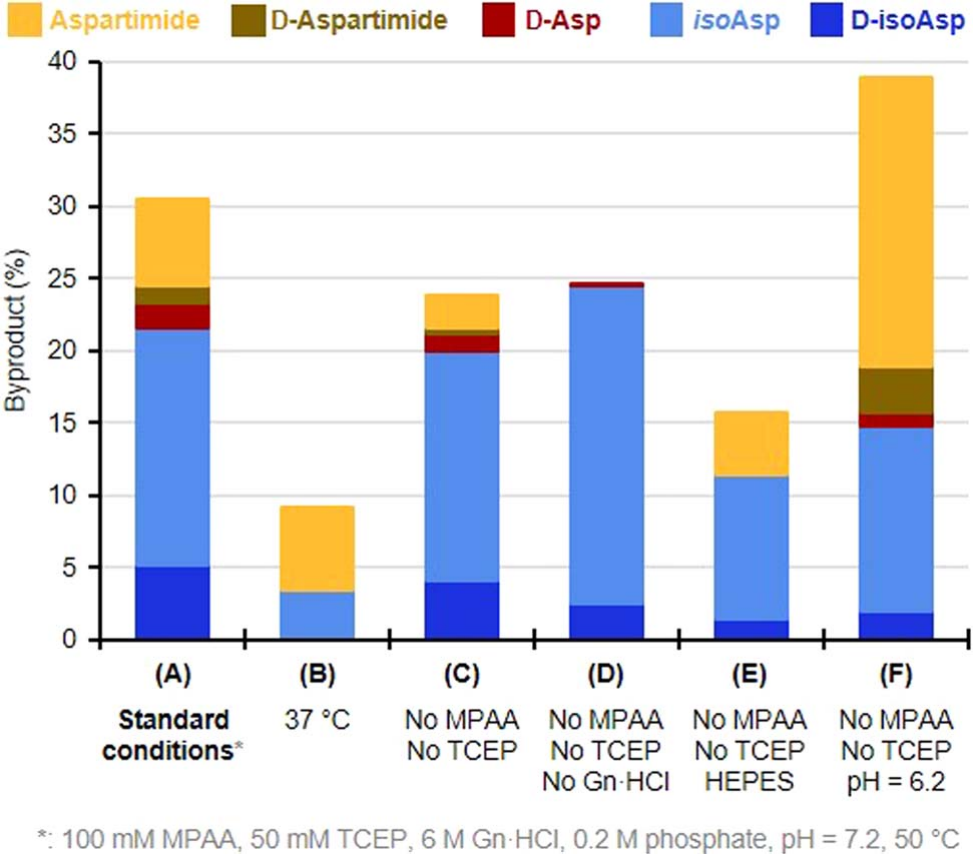
Effect of deviation from starting forcing NCL conditions on the formation of Asi and Asi-derived byproducts, evaluated using peptide 5 as a model substrate. All incubations were performed for 96 h.

We then turned to monitoring the formation of Asi and Asi-derived byproducts upon incubation of the native Asp peptide 5 under various NCL reaction conditions, starting with a classical NCL buffer, at 50 °C for eight days (Fig. 1). These NCL reaction conditions were deliberately forced in terms of temperature and time in order to exaggerate formation of byproducts and facilitate their quantification.^43^ We observed the formation of a peak (*c.a.* 9%) that co-eluted with the Asi standard (6), confirming that the product with an 18 Da mass loss formed during NCL was the aspartimide. Importantly, we were also able to observe the formation of isoAsp and epimerization products in considerable amounts, Asi only representing 20% of the total amount of byproducts. The incubation of an NG motif-containing peptide analogue under similar conditions also led to aspartimide and derived byproducts formation, in comparable amounts (Fig. S17†). Following these observations, we investigated which parameters were responsible for the formation of Asi during NCL in order to help prevent this in future ligation reactions. To do this, we screened a set of different conditions, either by omitting some of the NCL components (TCEP, MPAA, Gn·HCl), or by changing the pH, the temperature or the nature of the buffer (Fig. 2 and ESI, p. S19–S28†). Although none of the above variations did completely suppress the formation of Asi and Asi-derived byproducts after prolonged incubation, each set of conditions gave rise to different outcomes. For example, we observed that the use of phosphate as buffer significantly increased the extent of epimerization as well as Asi formation compared to HEPES (Fig. 2, entries C and E and S18†); or again, that guanidinium chloride exerted a protective effect against the hydrolysis of Asi. Consistent with earlier studies on the degradation of small peptides or protein,^25^ lowering the pH proved to have a strong effect: if the total amount of byproducts and their relative proportions do not significantly vary in the 6.7–7.7 pH range (corrected pH value^42^), a much higher rate of aspartimide was observed at pH 6.2 (Fig. S23–S26†).

Not surprisingly,^25,20*c*,*d*^ the parameter that most affected the occurrence of side reactions is temperature. Lowering it from 50 °C to 37 °C (entries 1 and 2) or 20 °C (Table 1) reduced the total amount of byproducts by nearly threefold, and more than twentyfold, respectively. This suggests that these reactions require significant activation energy. Although at 20 °C, the secondary reaction was not completely eliminated after a prolonged 8 days incubation, no traces of aspartimide or derived byproducts were observed after up to 4 days (Table 1). Despite the almost total inhibition of aspartimide formation at 20 °C with model peptide 5, earlier SUMO-2 synthesis carried out at this same temperature, either by us^20*d*^ or Melnyk's group,^20*c*^ resulted in 5% and 10% formation of aspartimide, respectively, after the different reaction times 20 h in our case, against 33 h for Melnyk's group required for completion of the ligations. Moreover, these values were determined by evaluation of the amount of M-18 Da compound by mass spectrometry, and thus do not take into account the isobaric Asi-derived byproducts that are likely formed in the course of the reactions.

**Table 1:** Influence of the temperature on the formation of Asi and Asi-derived byproducts during NCL conditions

Entry	Temperature	Time (h)	d-isoAsp (10) (%)	l-isoAsp (7) (%)	d-Asp (8) (%)	d-Asi (9) (%)	l-Asi (6) (%)	Total (%)	Asi/total byproducts (%)
1	50 °C	24	0.8	4.0	—	0.8	4.6	10.2	53.2
		96	5.1	16.4	1.8	1.2	6.0	30.5	23.6
		192	9.8	27.2	3.1	2.2	8.6	51.0	21.3
2	37 °C	24	—	—	—	—	2.5	2.5	100
		96	—	3.5	—	—	5.7	9.2	62.1
		192	1.2	7.2	0.6	0.9	7.6	17.4	49.0
3	20 °C	24	—	—	—	—	—	—	—
		96	—	—	—	—	—	—	—
		192	—	—	—	—	2.3	2.3	100

In the present comprehensive model study, we clearly demonstrated that aspartimide can be formed under NCL conditions. Our results suggest that this could be the source of side-reactions potentially occurring frequently during chemical protein synthesis. Such byproducts are likely to have been somewhat underestimated because of the furtive features of Asi and its low stability, resulting in byproducts with a mass equal to that of the target protein, and indistinguishable using standard HPLC/MS methods.^44^ Considering that the formation of aspartimide and its related byproducts can significantly alter the 3D structure and the activity of a protein, it is of prime importance to devise methods to prevent it. In our attempt to identify the NCL parameters promoting aspartimide formation, we established that the reaction can occur spontaneously and is probably mainly catalyzed by water rather than other NCL buffer components, as observed in previous biochemical studies and further supported by DFT simulations.^45^

The formation of aspartimide and derived byproducts can be limited by adopting “good NCL practices”, which involve restricting the ligation temperature and reaction times, as well as replacing the commonly used phosphate buffer with HEPES. However, the efficiency of such precautions may vary considerably depending on the Asi formation-propensity of the sequence of the target protein, which remains difficult to evaluate, even though DG- and NG-motifs containing sequences can be likely considered as the most problematic. Even more concerning, the amounts of Asi and its byproducts are expected to grow with the length of the target protein, as a result of the number of NCL reactions to be implemented and potential aspartimide hotspots (ESI, p. S29–S32†). This particularly true when intermediate products are not isolated and purified.

### A general synthetic strategy to suppress the formation of aspartimide

Therefore, we wished to develop a straightforward, efficient and generally-applicable strategy to totally suppress Asi formation at targeted sites during NCL. Inspired by strategies developed for SPPS, we decided to incorporate a protecting group on the backbone amide nitrogen atom of the residue C-terminal to the Asp residue involved in Asi-forming cyclization, thus making the side reaction impossible to occur.^22^ Our criteria for an ideal protecting group were that it should be: chemically-compatible with Fmoc-SPPS and NCL, easily incorporated into the peptide using inexpensive reagents and easily removable under mild conditions. We opted for the 2-hydroxy-4-methoxybenzyl (Hmb) moiety as a removable backbone modification, initially developed to counter peptide aggregation during SPPS,^46^ and later used for the same purpose during peptide purification^47^ and NCL.^48^ Importantly, *N*-Hmb protection is also a highly effective method to suppress aspartimide formation during Fmoc-SPPS.^49,50^ It can be incorporated in a peptide either using commercially available Hmb-containing building blocks, or through solid-phase reductive amination.^51^ Hmb is readily removed by trifluoroacetic acid (TFA) treatments used for peptide resin cleavage and deprotection after Fmoc-SPPS. In our case, we need to retain the Hmb protection while carrying out the NCL.

**Scheme 5:**
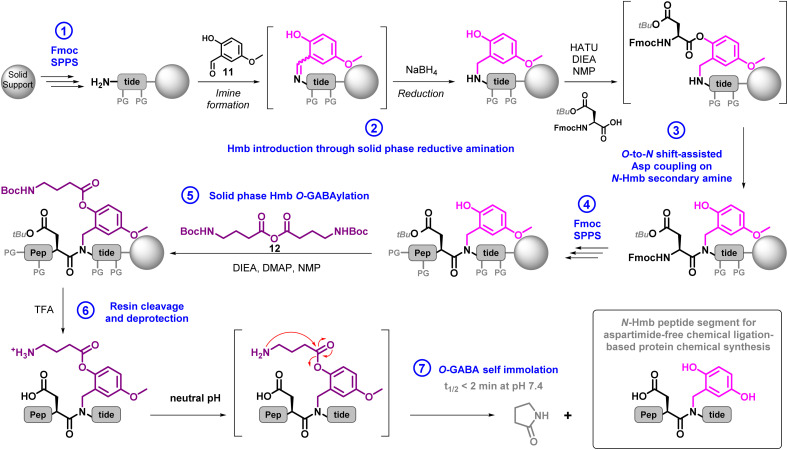
General strategy to synthesize Hmb-containing peptide segments. Steps 1–5 are fully automated on a standard peptide synthesizer. Step 6 makes use of standard TFA cleavage cocktails. Step 7 consists in simple dissolution in PBS buffer.

Conveniently, the sensitivity of Hmb to TFA can be completely switched off by acylating the 2-hydroxy group,^47,48,51^ commonly with an acetyl group. Acyl group removal restores the sensitivity towards TFA, mild conditions for most peptides. Deacetylation of Hmb can be conveniently carried out by a treatment with a large excess of hydrazine, typically as a 5% solution in DMF.^47*a*,48*a*^ However, we previously experienced an incompatibility of aqueous hydrazine with alkyne-containing peptides,^52^ and similar conditions were recently shown to promote severe oxidation of a small protein.^53^ We thus preferred to opt for a phenol ester that would be removed under mild neutral aqueous conditions through intramolecular aminolysis. Such a strategy has been initially introduced for the protection of tyrosine phenol by the *N*-methyl-*N*-[2-(methyl-amino)-ethyl]-carbamoyl (Nmec) group,^54^ later applied to an acid sensitivity-switchable peptide solubilizing tag,^55^ and an *S*-Hmb cysteine side chain protecting group.^56^ However, due to the modest electrophilicity of carbamate and low nucleophilicity of the secondary amine, the self-immolative cleavage of Nmec is relatively slow at neutral pH. We decided to set our sights on the gamma-aminobutyric acid ester (GABA) group, for which a fast self-immolation (Scheme 5, step 7) has been highlighted in other contexts.^57^

The synthesis of Hmb-equipped segments required the optimization of two key steps, (1) Hmb incorporation and (2) its solid-phase GABAylation (Scheme 5, steps 2 and 5, and ESI, p. S32–S41†). We opted for a reductive amination approach to incorporate the Hmb from the inexpensive 2-hydroxy-4-methoxybenzaldehyde (HmbA, 11).^58^ The resulting secondary amine benefits from the assistance of the 2-hydroxyl group of Hmb to facilitate the coupling of the next residue (Scheme 5, steps 3),^59^ in our case Asp(O*t*Bu) or Asn(Tr*t*). We first optimized conditions for Hmb incorporation using the SUMO-2[48–93] segment as a model.^60^ We found that a key point to ensure a quantitative reaction was the repetition of a long treatment with excess HmbA (2 treatments of 2 hours each with 10 equiv.) in a DMF/MeOH/AcOH 44.5 : 44.5 : 1 mixture to form the imine, before washing away the excess aldehyde and performing its reduction with sodium borohydride in DMF (ESI, p. S33†). Gratifyingly, these conditions were readily transposable to other sequences, including SUMO-2[1–47], and were easy to perform on a peptide synthesizer.

After Hmb incorporation and peptide elongation,^61^ GABAylation can be conveniently carried out using *N*-Boc GABA anhydride (12) as an acylating agent readily synthesized on a multi-gram scale (ESI, p. S34†). As optimal conditions for this reaction we found that using an excess of i-Pr_2_NEt and 12, in the presence of catalytic amounts of DMAP (1 mol%) in DMF, yielded very robust results (ESI, p. S36†). The Boc group of Boc-GABA-Hmb is cleaved during subsequent TFA-mediated peptide deprotection and cleavage from the resin, resulting in a GABA-Hmb-protected peptide, which is stable in its ammonium form, for example when using RP-HPLC solvents containing 0.1% TFA.^62^ Conversely, a rapid and clean self-immolation of the GABA moiety was observed upon incubation in a neutral aqueous buffer (PBS, pH 7.4), with a half reaction time (*t*_1/2_) of less than 2 min, and complete removal within half an hour. The reaction demonstrated typical first order kinetics (*k* = 0.09 s^−1^), and was several orders of magnitude faster than the hydrolysis of an acetyl group, which did not even reach 10% completion after 6 days incubation under the same conditions (ESI, p. S37–S38†).

### Aspartimide-free synthesis of SUMO-2 and derivatives

Having devised robust conditions for Hmb incorporation, the next step was to demonstrate the effectiveness of our strategy against Asi formation during NCL, and we chose to apply it to the synthesis of the problematic SUMO-2 protein.

The incorporation of Hmb was performed at the DG motifs of both segments. As in our previous report,^20*d*^ SUMO-2[1–47] (13a) was synthesized as a C-terminal *N*-Hnb-Cys cryptothioester^63^ and SUMO-2[48–93] segment (14a) was equipped with a (Lys)_6_-Ades solubilizing tag. The hexalysine of 14a was N-terminally modified with a GABA group during the peptide synthesiser-mediated automated process because we thought it unnecessary to keep the N-terminal amine of the tag for the solubilizing purposes, which would have required the coupling of the significantly more expensive Boc-Lys(Boc)-OH instead of the Fmoc-Lys(Boc)-OH standard Fmoc-SPPS building block.

We took the occasion of this work to improve conditions for the installation of the tag. Our initial strategy was based on the coupling of Boc-Cys(Npys)-OH (15), followed by reaction with excess 2-amino-1,1-dimethyl-ethane-1-thiol (16, ADET) to form the disulfide-based linker. A key feature of the Ades disulfide linker is the tertiary carbon bearing a sulfur atom within ADET, which makes the cysteine disulfide residue resistant to beta-elimination during piperidine-mediated Fmoc cleavage, inspired from the *S*-S*t*Bu Cys protecting group.^64,65^ However, we later found that our published protocol sometimes gave unreliable results, perhaps because of the unstability of Boc-Cys(Npys)-OH, either during its coupling or when standing in solution prior to delivery to the peptidyl resin by the automatic peptide synthesizer, as evidenced by the observation of peptides not incorporating the (Lys)_6_-Ades solubilizing tag. Additionally, this strategy requires a large excess of costly Boc-Cys(Npys)-OH and ADET. To overcome these limitations we decided to introduce the linker using a building-block, Boc-Cys(Fmoc-Ades)-OH (22). This compound is obtained in excellent yields through the reaction of stoichiometric amounts of Boc-Cys(Npys)-OH and ADET followed by one-pot protection of the Ades amino group using Fmoc-OSu (ESI, p. S44–S46†).

This building block can be readily coupled as the N-terminal Cys group of a peptide segment, followed by Fmoc-SPPS-based elongation to incorporate the hexalysine solubilizing sequence.

The crude 13a and 14a segments were analyzed by HPLC after GABA removal under neutral conditions, and indicated satisfactory Hmb incorporation, GABAylation and self-immolation of the GABA (ESI, p. S43–S48†).

**Fig. 3:**
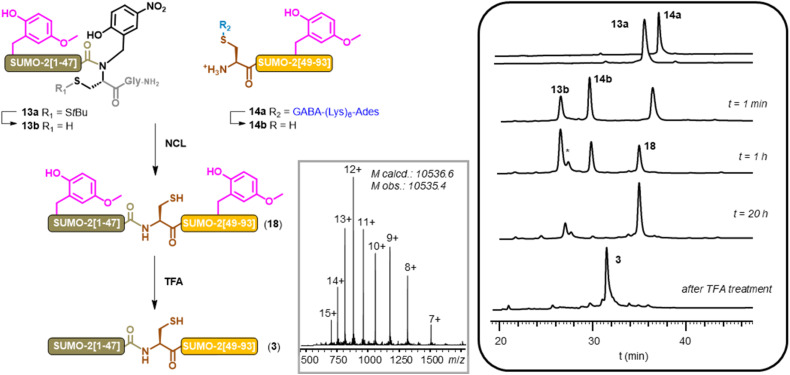
NCL-based aspartimide-free synthesis of SUMO-2 (NCL conditions: 200 mM phosphate, 6 M Gu·HCl, 100 mM MPAA, 50 mM TCEP, 37 °C). Grey box insert: MS of purified 3. *: HPLC peak corresponding to the MPAA thioester derivative of 13b.

Native chemical ligation of the two purified segments proceeded smoothly and led to the expected bis-Hmb SUMO-2 (18) after overnight incubation at 37 °C (Fig. 3). After a dialysis step to remove salts, TCEP and MPAA (82% recovery yield), the Hmb groups were removed by a TFA treatment followed by precipitation in diethyl ether, gratifyingly leading to a very clean crude SUMO-2 protein (3). A final HPLC purification afforded the pure compound in a satisfactory 21% yield. Most importantly, in the previous synthesis of SUMO-2, LC-MS analysis revealed approximately 15% of a M-18 Da byproduct, whereas no trace of such byproduct was detected using the Hmb-based strategy and applying strictly similar NCL conditions (ESI, p. S53†). These results unequivocally confirm that the byproduct observed in our first SUMO-2 synthesis was indeed due to the formation of aspartimide at the DG motifs. Furthermore, given its crucial role as an intermediate, the absence of aspartimide strongly suggests the absence of Asi-derived isobaric byproducts.

Enthused by these promising outcomes, we further challenged our methodology through the synthesis of a SUMOylated peptide. The preparation of such compounds has been previously reported. However, relying exclusively on NCL necessitates multiple steps of ligation and deprotection of terminal reactive groups.^35,66^ We opted to synthesize a SUMOylated peptide by combining NCL with another orthogonal ligation method, copper(i)-catalyzed azide/alkyne cycloaddition (CuAAC).^67^ The 1,4-disubstituted 1,2,3-triazole formed in this reaction is considered as an excellent mimic of an amide bond.^68^

**Fig. 4:**
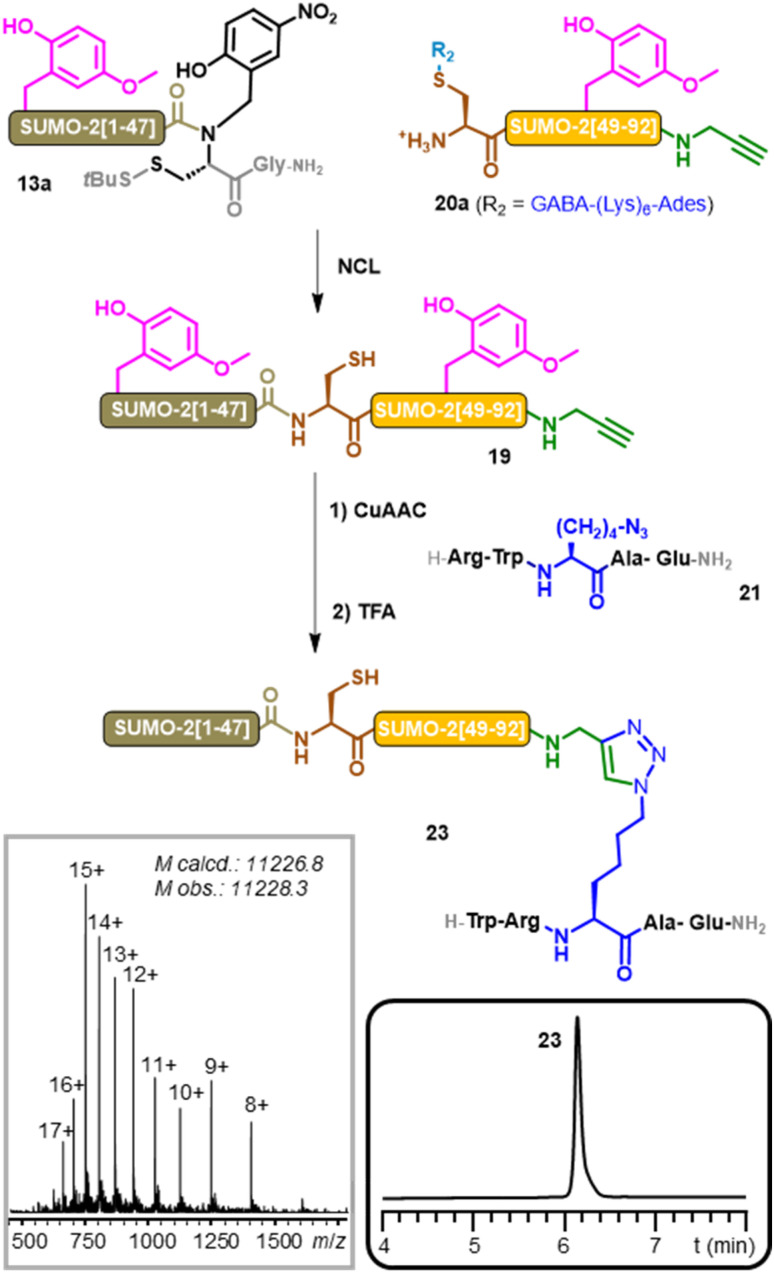
Aspartimide-free synthesis of SUMOylated peptide mimics 23 through a combination of native chemical ligation and peptidometic triazole ligation. (CuAAC condition: 200 mM HEPES/HFIP (1 : 1) 10 eq. CuS_4_, 30 eq. THPTA & 42.5 eq. sodium ascorbate, 37 °C) grey and black box inserts: MS and RP-HPLC analyses of purified 23.

Bis-Hmb-SUMO-2-alkyne 19 was obtained through NCL using a similar synthetic strategy as for bis-Hmb-SUMO-2 18 (ESI, p. S54–56†), from the NCL of crypto-thioester 13a and SUMO-2[48–92]-propargylamide segment 20a (Fig. 4).

Functionalization with an alkyne moiety was achieved by introducing a propargylamide in place of ^93^Gly in the [48–93] segment, using a backbone amide (BAL) linker^69^ (ESI, p. S49†).

The sequence of peptide 21 was inspired by the consensus motif of SUMOylation,^70^*Ψ*K*x*D/E (with *Ψ* = any hydrophobic residue, and *x* = any amino acid) and was obtained by incorporating azidonorleucine instead of lysine through Fmoc-SPPS (ESI, p. S57†).

The CuAAC ligation between 19 and 21 proceeded cleanly in a mixture of pH 8 HEPES buffer and hexafluoroisopropanol (HFIP)^71^ in presence of a catalytic system composed of CuSO_4_, the Cu(i) ligand THPTA, and sodium ascorbate as a copper(ii) ion reductant.^72^ Incubation at 37 °C during 4 h resulted in the complete conversion of 19 into the corresponding bis-Hmb SUMOylated peptide 22. The reaction was purified *via* HPLC before performing a TFA treatment to remove the Hmb groups.

A final purification step provided the desired SUMOylated peptide mimic 23 with an overall 28% yield, with no trace of M-18 Da byproduct being detected by MS. This final result highlights the applicability of our Hmb-method to chemical protein synthesis beyond the scope of NCL,^1^ which represents a simple and efficient strategy to obtain SUMOylated peptide mimics.

## Conclusions

In summary, we demonstrate in this work that formation of aspartimide and derived byproducts occurs during NCL and could be much more frequent than one might expect. We show that these side-reactions can be limited by restricting the ligation temperature and reaction times, as well as replacing the commonly used phosphate buffer with HEPES. However, the efficiency of such “good practices” is expected to vary considerably depending on the sequence of the target protein. Moreover, the amount of byproducts is expected to grow with its length, as a result of the number of potential aspartimide hotspots and NCL reactions implemented (Tables S4–S9†).

Facing these results, we propose a simple and potentially generally applicable methodology based on an *O*-GABAylated Hmb protecting group. This removable backbone modification is easily incorporated into the problematic site by automated solid phase reductive amination using the inexpensive 2-hydroxy-4-methoxybenzaldehyde. The *O*-acylation of Hmb by the GABA group is a key point in our strategy, as it switches off lability towards TFA-based peptidyl resin ending Fmoc-SPPS. The rapid self-immolation of the GABA ester under neutral conditions yields Hmb-equipped segments that are compatible with NCL, completely preventing formation of aspartimide at the incorporation site. The Hmb can be cleanly and efficiently removed from the synthesized protein through a final simple TFA treatment. The efficiency of this strategy was demonstrated through the aspartimide-free synthesis of the SUMO-2 protein and an analogue equipped with a C-terminal alkyne, as well as a mimic of a SUMOyalted peptide, further demonstrating the compatibility of the methodology with copper-catalyzed azide/alkyne cycloaddition, another chemoselective ligation reaction frequently used in peptide chemistry.

## Author contributions

EHC: data curation, investigation, methodology, validation, visualization, writing – original draft, writing – review & editing. VA: conceptualization, data curation, funding acquisition, methodology, resources, supervision, validation, visualization, writing – review & editing.

## Conflicts of interest

There are no conflicts to declare.

## Supplementary Material

SC-OLF-D5SC03824C-s001

## Data Availability

A comprehensive dataset supporting this article have been uploaded as part of the ESI,† including copies of NMR spectra, HPLC traces and MS spectra. Raw data (FID NMR, chromatograms, MS spectra) are available from the authors upon request.

## References

[cit1] Schnölzer M., Kent S. B. H. (1992). Constructing Proteins by Dovetailing Unprotected Synthetic Peptides: Backbone-Engineered HIV Protease. Science.

[cit2] Merrifield R. B. (1963). Solid Phase Peptide Synthesis: I. The Synthesis of a Tetrapeptide. J. Am. Chem. Soc..

[cit3] Dawson P. E., Muir T. W., Clark-Lewis I., Kent S. B. H. (1994). Synthesis of Proteins by Native Chemical Ligation. Science.

[cit4] Saxon E., Armstrong J. I., Bertozzi C. R. (2000). A “Traceless” Staudinger Ligation for the Chemoselective Synthesis of Amide Bonds. Org. Lett..

[cit5] Bode J. W. (2017). Chemical Protein Synthesis with the α-Ketoacid–Hydroxylamine Ligation. Acc. Chem. Res..

[cit6] Liu H., Li X. (2018). Serine/Threonine Ligation: Origin, Mechanistic Aspects, and Applications. Acc. Chem. Res..

[cit7] Hartrampf N., Saebi A., Poskus M., Gates Z. P., Callahan A. J., Cowfer A. E., Hanna S., Antilla S., Schissel C. K., Quartararo A. J., Ye X., Mijalis A. J., Simon M. D., Loas A., Liu S., Jessen C., Nielsen T. E., Pentelute B. L. (2020). Synthesis of Proteins by Automated Flow Chemistry. Science.

[cit8] Kulkarni S. S., Sayers J., Premdjee B., Payne R. J. (2018). Rapid and Efficient Protein Synthesis through Expansion of the Native Chemical Ligation Concept. Nat. Rev. Chem..

[cit9] Agouridas V., El Mahdi O., Cargoët M., Melnyk O. (2017). A Statistical View of Protein Chemical Synthesis Using NCL and Extended Methodologies. Bioorg. Med. Chem..

[cit10] Sun H., Brik A. (2019). The Journey for the Total Chemical Synthesis of a 53 KDa Protein. Acc. Chem. Res..

[cit11] Fan C., Deng Q., Zhu T. F. (2021). Bioorthogonal Information Storage in L-DNA with a High-Fidelity Mirror-Image Pfu DNA Polymerase. Nat. Biotechnol..

[cit12] Kamo N., Hayashi G., Okamoto A. (2018). Triple Function of 4-Mercaptophenylacetic Acid Promotes One-Pot Multiple Peptide Ligation. Angew. Chem., Int. Ed..

[cit13] Abboud S. A., Amoura M., Madinier J., Renoux B., Papot S., Piller V., Aucagne V. (2021). Enzyme-Cleavable Linkers for Protein Chemical Synthesis through Solid-Phase Ligations. Angew. Chem., Int. Ed..

[cit14] Fang G., Li Y., Shen F., Huang Y., Li J., Lin Y., Cui H., Liu L. (2011). Protein Chemical Synthesis by Ligation of Peptide Hydrazides. Angew. Chem., Int. Ed..

[cit15] Zhao D. D., Fan X. W., Hao H., Zhang H. L., Guo Y. (2019). Temporary Solubilizing Tags Method for the Chemical Synthesis of Hydrophobic Proteins. Curr. Org. Chem..

[cit16] Jin K., Li X. (2018). Advances in Native Chemical Ligation–Desulfurization: A Powerful Strategy for Peptide and Protein Synthesis. Chem. – Eur. J..

[cit17] Loibl S. F., Harpaz Z., Zitterbart R., Seitz O. (2016). Total Chemical Synthesis of Proteins without HPLC Purification. Chem. Sci..

[cit18] Xu W., Jiang W., Wang J., Yu L., Chen J., Liu X., Liu L., Zhu T. F. (2017). Total Chemical Synthesis of a Thermostable Enzyme Capable of Polymerase Chain Reaction. Cell Discovery.

[cit19] Snella B., Diemer V., Drobecq H., Agouridas V., Melnyk O. (2018). Native Chemical Ligation at Serine Revisited. Org. Lett..

[cit20] (b) Bouchenna J. , Sénéchal M., Drobecq H., Vicogne J. and Melnyk O., The Problem of Aspartimide Formation During Protein Chemical Synthesis Using SEA-Mediated Ligation, In Peptide and Protein Engineering, Ed.O. Iranzo and A. C. Roque, Springer Protocols Handbooks, New York, NY, 2020, pp 13–28

[cit21] Lelièvre D., Terrier V. P., Delmas A. F., Aucagne V. (2016). Native Chemical Ligation Strategy to Overcome Side Reactions during Fmoc-Based Synthesis of C-Terminal Cysteine-Containing Peptides. Org. Lett..

[cit22] Subirós-Funosas R., El-Faham A., Albericio F. (2011). Aspartimide Formation in Peptide Chemistry: Occurrence, Prevention Strategies and the Role of N-Hydroxylamines. Tetrahedron.

[cit23] Neumann K., Farnung J., Baldauf S., Bode J. W. (2020). Prevention of Aspartimide Formation during Peptide Synthesis Using Cyanosulfurylides as Carboxylic Acid-Protecting Groups. Nat. Commun..

[cit24] Clarke S. (2003). Aging as War between Chemical and Biochemical Processes: Protein Methylation and the Recognition of Age-Damaged Proteins for Repair. Ageing Res. Rev..

[cit25] Geiger T., Clarke S. (1987). Deamidation, Isomerization, and Racemization at Asparaginyl and Aspartyl Residues in Peptides. Succinimide-Linked Reactions That Contribute to Protein Degradation. J. Biol. Chem..

[cit26] Elashal H. E., Koos J. D., Cheung-Lee W. L., Choi B., Cao L., Richardson M. A., White H. L., Link A. J. (2022). Biosynthesis and Characterization of Fuscimiditide, an Aspartimidylated Graspetide. Nat. Chem..

[cit27] Becker C. F. W., Hunter C. L., Seidel R., Kent S. B. H., Goody R. S., Engelhard M. (2003). Total Chemical Synthesis of a Functional Interacting Protein Pair: The Protooncogene H-Ras and the Ras-Binding Domain of Its Effector c-Raf1. Proc. Natl. Acad. Sci. U.S.A.

[cit28] Melnyk O., Vicogne J. (2016). Total chemical synthesis of SUMO proteins. Tetrahedron Lett..

[cit29] Meluh P. B., Koshland D. (1995). Evidence That the MIF2 Gene of Saccharomyces Cerevisiae Encodes a Centromere Protein with Homology to the Mammalian Centromere Protein CENP-C. Mol. Biol. Cell.

[cit30] Cappadocia L., Lima C. D. (2018). Ubiquitin-like Protein Conjugation: Structures, Chemistry, and Mechanism. Chem. Rev..

[cit31] Celen A. B., Sahin U. (2020). Sumoylation on Its 25th Anniversary: Mechanisms, Pathology, and Emerging Concepts. FEBS J..

[cit32] Mandel N., Agarwal N. (2022). Role of SUMOylation in Neurodegenerative Diseases. Cells.

[cit33] Terrier V. P., Adihou H., Arnould M., Delmas A. F., Aucagne V. A. (2016). Straightforward Method for Automated Fmoc-Based Synthesis of Bio-Inspired Peptide Crypto-Thioesters. Chem. Sci..

[cit34] Bi S., Liu P., Ling B., Yuan X., Jiang Y. (2018). Mechanism of N-to-S Acyl Transfer of N-(2-Hydroxybenzyl) Cysteine Derivatives and Origin of Phenol Acceleration Effect. Chin. Chem. Lett..

[cit35] Bondalapati S., Eid E., Mali S. M., Wolberger C., Brik A. (2017). Total Chemical Synthesis of SUMO-2-Lys63-Linked Diubiquitin Hybrid Chains Assisted by Removable Solubilizing Tags. Chem. Sci..

[cit36] Johnson E. C. B., Kent S. B. H. (2006). Insights into the Mechanism and Catalysis of the Native Chemical Ligation Reaction. J. Am. Chem. Soc..

[cit37] Ollivier N., Dheur J., Mhidia R., Blanpain A., Melnyk O. (2010). Bis(2-Sulfanylethyl)Amino Native Peptide Ligation. Org. Lett..

[cit38] Lewis U. J., Cheever E. V., Hopkins W. C. (1970). Kinetic Study of the Deamidation of Growth Hormone and Prolactin. Biochim. Biophys. Acta, Nucleic Acids Protein Synth..

[cit39] Radkiewicz J. L., Zipse H., Clarke S., Houk K. N. (2001). Neighboring Side Chain Effects on Asparaginyl and Aspartyl Degradation: An Ab Initio Study of the Relationship between Peptide Conformation and Backbone NH Acidity. J. Am. Chem. Soc..

[cit40] McKerrow J. H., Robinson A. B. (1974). Primary Sequence Dependence of the Deamidation of Rabbit Muscle Aldolase. Science.

[cit41] Radkiewicz J. L., Zipse H., Clarke S., Houk K. N. (1996). Accelerated Racemization of Aspartic Acid and Asparagine Residues via Succinimide Intermediates: An Ab Initio Theoretical Exploration of Mechanism. J. Am. Chem. Soc..

[cit42] Garcia-Mira M. M., Sanchez-Ruiz J. M. (2001). pH Corrections and Protein Ionization in Water/Guanidinium Chloride. Biophys. J..

[cit43] Asi formation at 37°C is significantly slower for model peptide 5 than for the longer segments 1 and 2a (5.8% versus 12% after 3 days)

[cit44] Ying Y., Li H. (2022). Recent Progress in the Analysis of Protein Deamidation Using Mass Spectrometry. Methods.

[cit45] Takahashi O. (2013). Two-Water-Assisted Racemization of the Succinimide Intermediate Formed in Proteins, A Computational Model Study. Health.

[cit46] Johnson T., Quibell M., Owen D., Sheppard R. C. (1993). A Reversible Protecting Group for the Amide Bond in Peptides. Use in the Synthesis of ‘Difficult Sequences. J. Chem. Soc., Chem. Commun..

[cit47] Quibell M., Turnell W. G., Johnson T. (1994). Preparation and Purification of β-Amyloid (1-43) via Soluble, Amide Backbone Protected Intermediates. J. Org. Chem..

[cit48] (a) Abdel-Aal A. B. M. , Raz R., Papageorgiou G., and Offer J., Synthesis of Amide Backbone-Modified Peptides Peptide Synthesis, In Methods in Molecular Biology, Ed.W. M. Hussein, M. Skwarczynski and I. Toth, Springer US, New York, 2020, Chapter 15, 2103, 225–237 10.1007/978-1-0716-0227-0_15 31879929

[cit49] Quibell M., Owen D., Packman L. C., Johnson T. (1994). Suppression of Piperidine-Mediated Side Product Formation for Asp(OBut)-Containing Peptides by the Use of N-(2-Hydroxy-4-Methoxybenzyl) (Hmb) Backbone Amide Protection. J. Chem. Soc. Chem. Commun..

[cit50] Cui T., Chen J., Zhao R., Guo Y., Tang J., Li Y., Bierer D., Liu L. (2021). Use of a Removable Backbone Modification Strategy to Prevent Aspartimide Formation in the Synthesis of Asp Lactam Cyclic Peptides. Chin. J. Chem..

[cit51] Ede N. J., Ang K. H., James I. W., Bray A. M. (1996). Incorporation of 2-hydroxy-4-methoxybenzyl protection during peptide synthesis via reductive alkylation on the solid phase. Tetrahedron Lett..

[cit52] Galibert M., Piller V., Piller F., Aucagne V., Delmas A. F. (2015). Combining Triazole Ligation and Enzymatic Glycosylation on Solid Phase Simplifies the Synthesis of Very Long Glycoprotein Analogues. Chem. Sci..

[cit53] Han J., Dang B. (2025). Optimizing Solid-Phase Protein Synthesis Using CPG-2000 and a Nickel-Cleavable SNAC-tag Linker. Org. Lett..

[cit54] Wahlström K., Planstedt O., Undén A. A. (2008). Carbamoyl-Protective Group for Tyrosine That Facilitates Purification of Hydrophobic Synthetic Peptides. Tetrahedron Lett..

[cit55] Zheng J. S., Yu M., Qi Y.-K., Tang S., Shen F., Wang Z.-P., Xiao L., Zhang L., Tian C. L., Liu L. (2014). Expedient Total Synthesis of Small to Medium-Sized Membrane Proteins via Fmoc Chemistry. J. Am. Chem. Soc..

[cit56] Qi Y.-K., Tang S., Huang Y. C., Pan M., Zheng J. S., Liu L. (2016). Hmb off/on as a Switchable Thiol Protecting Group for Native Chemical Ligation. Org. Biomol. Chem..

[cit57] DeWit M. A., Gillies E. R. (2011). Design, Synthesis, and Cyclization of 4-Aminobutyric Acid Derivatives: Potential Candidates as Self-Immolative Spacers. Org. Biomol. Chem..

[cit58] (a) Abdel-Aal A.-B. M. , Raz R., Papageorgiou G. and Offer J., Synthesis of Amide Backbone-Modified Peptides Peptide Synthesis, In Methods in Molecular Biology, Ed W. M. Hussein, M. Skwarczynski and I. Toth, Springer US, New York, NY, 2020, Chapter 15, 2103, 225–237 10.1007/978-1-0716-0227-0_15 31879929

[cit59] Johnson T., Quibell M., Owen D., Sheppard R. C. (1993). A Reversible Protecting Group for the Amide Bond in Peptides. Use in the Synthesis of ‘Difficult Sequences. J. Chem. Soc., Chem. Commun..

[cit60] Optimization of the conditions for Hmb incorporation conditions were first undertook using a short peptide with the same sequence as 5 (ESI, p. S33†). The optimized conditions were readily applicable to a NG motif-containing derivative, where the Asp(OtBu) residue was replaced by an Asn(Trt) (peptide S9, ESI, p. S40†). Disappointingly, these conditions did not enable quantitative incorporation into longer peptide segments. Consequently, we carried out a second optimization tailored for longer peptides, using the SUMO-2[48-93] segment, the key point being the repetition of a long treatment with excess HmbA (ESI, p. S33†)

[cit61] The unprotected phenol group of the Hmb moiety is partially acylated during each AA coupling SPPS step and subsequent acetic anhydride-based capping of potentially unreacted amine groups. The ester formed is readily cleaved during the following piperidine treatment used to deprotect the N-Fmoc group. Therefore, the last AA coupled, N-terminal of the sequence, required to be protected with a TFA cleavage-labile group (N-Boc), and its coupling is followed by a piperidine treatment to ensure recovering free phenol-containing Hmb for subsequent Boc-GABAylation. This precaution was not necessary for the incorporation of the Lys6 solubilizing sequence cleaved during the NCL, which was thus terminated with a hydrophilic GABA group, bearing a positive charge under acidic HPLC conditions

[cit62] The free phenol group of the N-Hnb group of the crypto-thioester device is also O-GABylated during the synthetic process. Contrasting with GABA-Hmb, we observed slow self-immolation of the GABA-Hnb under the acidic conditions used for HPLC purification (0.1% TFA, pH ≈ 2), and thus preferred to perform a neutral pH treatment to self-immolate all GABA esters prior to segment purification and subsequent NCL, a precaution that should be unnecessary for peptide segments not containing an Hnb

[cit63] Abboud S. A., Aucagne V. (2020). An Optimized Protocol for the Synthesis of N -2-Hydroxybenzyl-Cysteine Peptide Crypto-Thioesters. Org. Biomol. Chem..

[cit64] (b) Andreu D. , Albericio F., Solé N. A., Munson M. C., Ferrer M. and Barany G., Formation of Disulfide Bonds in Synthetic Peptides and Proteins. In: Peptide Synthesis Protocols. Methods in Molecular Biology, ed M. W. Pennington, and B. M. Dunn, 1994, 35 10.1385/0-89603-273-6:91 7894611

[cit65] Liu J., Wei T., Tan Y., Liu H., Li X. (2022). Enabling chemical protein (semi)synthesis via reducible solubilizing tags (RSTs). Chem. Sci..

[cit66] Bouchenna J., Sénéchal M., Drobecq H., Vicogne J., Melnyk O. (2019). Total Chemical Synthesis of All SUMO-2/3 Dimer Combinations. Bioconjugate Chem..

[cit67] Rostovtsev V. V., Green L. G., Fokin V. V., Sharpless K. B. (2002). A Stepwise Huisgen Cycloaddition Process: Copper(I)-Catalyzed Regioselective “Ligation” of Azides and Terminal Alkynes. Angew. Chem., Int. Ed..

[cit68] Valverde I. E., Lecaille F., Lalmanach G., Aucagne V., A F. (2012). Delmas Synthesis of a Biologically Active Triazole-Containing Analogue of Cystatin A Through Successive Peptidomimetic Alkyne-Azide Ligations. Angew. Chem., Int. Ed..

[cit69] Jensen K. J., Alsina J., Songster M. F., Vágner J., Albericio F., Barany G. (1998). Backbone Amide Linker (BAL) Strategy for Solid-Phase Synthesis of C-Terminal-Modified and Cyclic Peptides. J. Am. Chem. Soc..

[cit70] Hendriks I. A., D'Souza R. C. J., Yang B., Verlaan-de Vries M., Mann M., Vertegaal A. C. O. (2014). Uncovering Global SUMOylation Signaling Networks in a Site-Specific Manner. Nat. Struct. Mol. Biol..

[cit71] Peschke F., Taladriz-Sender A., Andrews M. J., Watson A. J. B., Burley G. A. (2023). Glutathione Mediates Control of Dual Differential Bio-orthogonal Labelling of Biomolecules. Angew. Chem. Int. Ed.

[cit72] Hong V., Presolski S. I., Ma C., Finn M. G. (2009). Analysis and Optimization of Copper-Catalyzed Azide–Alkyne Cycloaddition for Bioconjugation. Angew. Chem..

