# Supplementary material for: Identification, occurrence and prevention of aspartimide-related byproducts in chemical protein synthesis
Source: Chem Sci. 2025 Jul 8;16(32):14496–508. doi: 10.1039/d5sc03824c (PMC12262139; doi:10.1039/d5sc03824c)
Supplement: SC-016-D5SC03824C-s001 [file SC-016-D5SC03824C-s001.pdf]

Supplementary Information (SI) for Chemical Science.
This journal is © The Royal Society of Chemistry 2025

Identification, occurrence and prevention of aspartimide-related byproducts in chemical protein synthesis

El hadji CISSE and Vincent Aucagne

Centre de Biophysique Moléculaire, CNRS UPR 4301, Rue Charles Sadron, 45071 Orléans Cedex 2, France.
E-mail : vincent.aucagne@cnrs-orleans.fr

Supporting information

1 General information ... 3
2 General procedures for solid phase peptide synthesis ... 4
3 Incubation of SUMO-2 protein (**3**) under physiological conditions. ... 6
4 Incubation of SUMO-2 segments under NCL conditions. ... 6
5 Synthesis of HPLC standards to monitor Asi and related byproducts formation on a model peptide sequence ... 8
5.1 Synthesis of native model peptide (**5**) ... 8
5.2 Synthesis of L-Asi and L-*iso*Asp-containing peptides (**6** & **7**) ... 10
5.3 Synthesis of D-Asp-containing model peptide (**8**) ... 12
5.4 Synthesis of D-Asi- and D-*iso*Asp-containing peptides (**9** & **10**) ... 13
5.5 Synthesis of Asn-containing peptide (**S01**) ... 16
6 Separation of the model peptide and analytical standards by HPLC. ... 17
7 Monitoring of Asi hydrolysis and epimerization ... 18
8 Monitoring of Asi formation during various NCL condition incubation ... 19
8.1 Incubation of model peptide **5** under NCL conditions ... 19
8.2 Incubation of NG-containing model peptide **S01** under NCL conditions ... 19
8.3 Influence of the buffer on the rate of formation of Asi and related byproducts ... 20
8.4 Influence of the pH on the rate of formation of Asi and related byproducts. ... 23
8.5 Influence of the temperature on the rate of formation of Asi and related byproducts. ... 27
9 Estimation of the cumulated amount of aspartimide and related byproducts formed through the assembly of a protein by multiple successive NCLs ... 29
10 Development of the Hmb strategy ... 32
10.1 Acetylation conditions ... 32
10.2 Hmb incorporation through solid-supported reductive amination ... 32
10.3 Solid phase *O*-GABAylation of Hmb ... 34
10.3.1 Synthesis of *N*-Boc-γ-aminobutanoic anhydride (**12**) ... 34

10.4 Monitoring of the self-immolation of the GABA group on the Hmb ... 36
10.5 Synthesis of [64]Gly-*N*-[Ac]Hmb-SUMO-2[61-67]W-NH_2 (**S07**)... 39
10.6 Synthesis of [64]Gly-*N*-[Ac]Hmb-SUMO-2[61-67]W-NH_2 D63N (**S09**)... 40
10.7 Estimation of the molar extinction coefficient of the Hmb group at λ = 280 nm ... 41
11 Synthesis and purification of SUMO-2 derived peptide segments ... 43
11.1 [27]Gly-*N*-Hmb SUMO-2[1-46]-(Hnb)Cys(S*t*Bu)-Gly-NH_2 crypto-thioester (**13a**)... 43
11.2 Synthesis of the SUMO-2 C-ter segments **14a** and **20a** ... 44
11.2.1 Synthesis of Boc-Cys(Fmoc-Ades)-OH (**17**)... 44
11.2.2 [64]Gly-*N*-Hmb Cys(K_6-Ades)-SUMO-2[48-93]-OH (**14a**)... 47
11.2.3 [64]Gly-*N*-Hmb Cys(K_6-Ades)-SUMO-2[48-92] propargylamide (**20a**) ... 49
12 Native chemical ligation ... 50
12.1 Aspartimide-free synthesis of SUMO-2 (**3**)... 50
12.2 Synthesis of a SUMO-2 protein equipped with a C-terminal alkyne moiety... 54
13 Chemical SUMOylation... 57
13.1 Synthesis of azido model peptide (**21**) ... 57
13.2 CuAAC reaction between **19** and **21**... 58
14 Bibliography... 60

1 General information

All reagents and solvents were used without further purification. Protected amino acids were purchased from Gyros Protein Technology (Uppsala, Sweden). 1-Amino-2-methyl-2-propanethiol hydrochloride (Ades·HCl) was purchased from Key organics (Camelford, UK). DIEA was purchased from Carlo Erba (Val-de-Reuil, France). Rink amide ChemMatrix resin was purchased from Biotage (Uppsala, Sweden). Peptide synthesis grade DMF was obtained from VWR (Fontenay-sous-Bois, France). Fmoc-Cys(NPys)-OH was purchased from BACHEM (Bubendorf, Switzerland). All other chemicals were from Merck-Sigma-Aldrich (St-Quentin-Fallavier, France) and solvents from Carlo Erba. Ultrapure water was obtained using a Milli-Q water system from Millipore (Molsheim, France). Polypropylene syringes fitted with polypropylene frits were obtained from Torviq (Niles, MI, USA) and were equipped with PTFE stopcocks bought from Biotage. HPLC analyses were carried out on a Chromaster system equipped with a 5160 pump, a 5430 diode array detector and a 5260 auto sampler, semi-preparative purifications were carried out on a LaChromElite system equipped with a Hitachi L-2130 pump, a Hitachi L-2455 diode array detector and a Hitachi L-2200 auto sampler and preparative purification were carried out on Nexera prep system equipped with a Shimadzu LC-20AT pump, a Shimadzu SPD-40 UV-visible detector, a Shimadzu SCL-40 controller and a Shimadzu FRC-10A fraction collector**.** Chromolith High Resolution RP-18e (150 Å, 10 × 4.6 mm, 3 mL/min flow rate), Jupiter C4 (300 Å, 5 µm, 250 × 4.6 mm, 1 mL/min flow rate) or Nucleosil C18 (300 Å, 5 µm, 250 × 4.6 mm, 1 mL/min flow rate) columns were used for analysis. Nucleosil C18 (300 Å, 5 µm, 250 × 10 mm, 3 mL/min flow rate) and Nucleosil C18 (300 Å, 5 µm, 250 × 32 mm, 30 mL/min flow rate) columns were used for semi-preparative and preparative purifications, respectively. Solvents A and B are 0.1 % TFA in H_2O and 0.1 % TFA in MeCN, respectively. Each gradient was followed by a washing step to elute any compound not eluted during the gradient (up to 95% B/A over 0.5 min, then isocratic 95% B/A for 0.5 min for the HR Chromolith). LC-MS analyses were carried out on an Agilent 1260 Infinity HPLC system, coupled with an Agilent 6120 mass spectrometer (ESI + mode), and fitted with an Aeris Widepore XB-C18 2 (3.6 µm, 150 × 2.1 mm, 0.5 mL/min flow rate, 60 °C) column. Solvents A' and B' were 0.1 % formic acid in H_2O and 0.1 % formic acid in MeCN, respectively. Gradient: 3% B'/A' for 0.6 min, then 3 to 50% B'/A' over 10.8 min. Low resolution MS of pure compounds were obtained using this system. The reported *m/z* values correspond either to the average or monoisotopic ions as specified in the characterization descriptions. When needed, the multiply-charged envelope was deconvoluted using the charge deconvolution tool in Agilent OpenLab CDS ChemStation software to obtain the average [M] value. High resolution ESI-MS analyses were performed on a maXis™ ultra-high-resolution Q-TOF mass spectrometer (Bruker Daltonics, Bremen, Germany), using the positive mode. The multiply-charged envelope was deconvoluted using the charge deconvolution algorithm in Bruker Data Analysis 4.1 software to obtain the monoisotopic [M] value. Dialysis was performed with GeBaFlex-tubes from Gene Bio-Application Ltd. (Yavne, Israel)

For yield calculations purposes, the quantities of purified peptides were determined by UV quantification at 280 nm using the molar absorption coefficient (ε) of Trp[1] (5500 $M^{-1}\cdot cm^{-1}$), Tyr[1] (1400 $M^{-1}\cdot cm^{-1}$), *N*-2-hydroxy-5-nitrobenzyl[2] (3440 $M^{-1}\cdot cm^{-1}$) and *N*-2-hydroxy-4-methoxybenzyl (1607 $M^{-1}\cdot cm^{-1}$, see p. S42).

The presence of Gn·HCl is known to bias pH measurements obtained with a pH meter. In our work, the concentration of Gu-HCl used is 6 M, at this concentration δpH (= pHactual – pHapparent) is +0.72. [3]

2 General protocols

Protocol PS1 -Peptide synthesis: automated Fmoc-based solid phase peptide syntheses (SPPS) were carried out on a Prelude synthesizer from Protein technologies. Manual couplings were performed on polypropylene syringes fitted with polypropylene frits using rotation stirring. The side-chain protecting groups used for Fmoc L-amino acids were Arg(Pbf), Asn(Trt), Asp(O*t*Bu), Cys(Trt), Cys(S*t*Bu), Glu(O*t*Bu), Gln(Trt), His(Trt), Lys(Boc), Ser(*t*Bu), Thr(*t*Bu), Trb(Boc) and Tyr(*t*Bu). Boc-Lys(Boc) and Boc-Nle were also used. Syntheses were performed on a 0.050 mmol-per-reactor scale. Protected amino acids (0.5 mmol, 10 equiv.) in DMF (1 mL) were coupled for 30 minutes using HATU (180 mg, 0.475 mmol, 9.5 equiv.) in DMF (1 mL), DIEA (174 µL, 0.1 mmol, 20 equiv.) in NMP (1 mL) and additional NMP (1 mL). Couplings on *N*-Hnb-cysteine and *N*-Hmb-glycine secondary amines were performed through three successive couplings for 2 h. Fmoc-Lys(N_3)-OH was incorporated using 10 equiv., with 9.5 equiv. of HATU and 20 equiv. of DIEA during 12 h. Capping of possible unreacted amine groups was achieved by treatment with acetic anhydride (286 µL, 3.02 mmol, 60 equiv.), DIEA (136 µL, 0.8 mmol, 15.5 equiv.) and HOBt (12 mg, 0.088 mmol, 1.8 equiv.) in NMP (5 mL) for 7 min (4 x 7 min in the case of *N*-Hnb-cysteine and *N*-Hmb-glycine secondaries amines). Fmoc group was deprotected by three successive treatments with 20% *v*/*v* piperidine in NMP (4 mL) for 3 min.

Protocol PS2 -Reductive amination: introduction of the 2-hydroxy-5-nitrobenzyl (Hnb) group : 50 µmol of H-Cys(S*t*Bu)-Gly-Rink-resin was washed with 1:1 DMF/MeOH (4 x 3 mL, 30 s). 2-Hydroxy-5-nitrobenzaldehyde (84 mg, 10 equiv.) in 4 mL 44.5:44.5:1 DMF/MeOH/AcOH (125 mM aldehyde concentration) was then added, and the resin was stirred for 5 min. The reactor was drained and the resin was washed with 1:1 DMF/MeOH (3 x 3 mL, 5 s) then DMF (3 x 3 mL, 5 s). Without delay, a fresh solution of sodium borohydride (38 mg, 20 equiv.) in 4 mL DMF (250 mM borohydride concentration) was added and the reactor was stirred for 20 min. The reactor was drained and the resin was washed with DMF (4 × 5 mL, 30 s), 20% *v*/*v* piperidine in NMP (3 × 5 mL, 3 min), NMP (3 × 5 mL, 30 s), dichloromethane (3 × 5 mL, 30 s) and NMP (3 × 5 mL, 30 s).

Protocol PS3 -Optimized reductive amination: introduction of the 2-hydroxy-4-methoxybenzyl (Hmb) group: - *For short sequence (model peptide)*: 50 µmol of Gly-peptidyl resin was washed with 1:1 DMF/MeOH (4 x 3 mL, 30 s). 2-Hydroxy-5-methoxybenzaldehyde (76 mg, 10 equiv.) in 4 mL 44.5:44.5:1 DMF/MeOH/AcOH (125 mM aldehyde concentration) was then coupled during 1 h. The reactor was drained and the resin was washed with 1:1 DMF/MeOH (3 x 3 mL, 5 s) then DMF (3 x 3 mL, 5 s). Without delay, a fresh solution of sodium borohydride (38 mg, 20 equiv.) in 4 mL DMF (250 mM borohydride concentration) was added and the reactor was stirred for 20 min. The reactor was drained and the resin was washed with DMF (4 × 5 mL, 30 s), 20% *v*/*v* piperidine in NMP (3 × 5 mL, 3 min), NMP (3 × 5 mL, 30 s), dichloromethane (3 × 5 mL, 30 s) and NMP (3 × 5 mL, 30 s).

- *For long sequence* (SUMO-2 segments): 50 µmol of Gly-peptidyl resin was washed with 1:1 DMF/MeOH (4 x 3 mL, 30 s). 2-Hydroxy-5-methoxybenzaldehyde (76mg, 10 equiv.) in 4 mL 44.5:44.5:1 DMF/MeOH/AcOH (125mM aldehyde concentration) was then coupled twice successively, during 2h. The reactor was drained and the resin was washed with 1:1 DMF/MeOH (3 x 3 mL, 5 s) then DMF (3 x 3 mL, 5 s). Without delay, a fresh solution of sodium borohydride (38mg, 20 equiv.) in 4 mL DMF (250mM borohydride concentration) was added and the reactor was stirred for 20min. The reactor was drained and the resin was washed with DMF (4 × 5 mL, 30 s), 20% *v*/*v* piperidine in NMP (3 × 5 mL, 3min), NMP (3 × 5 mL, 30 s), dichloromethane (3 × 5 mL, 30 s) and NMP (3 × 5 mL, 30 s).

Protocol PS4 -Selective Hnb and Hmb ester cleavage to allow UV titration of Fmoc deprotection: as a consequence of the formation of variable amount (5-90%) of *O*-acylated Hnb and Hmb during each coupling, this ester being cleaved upon piperidine treatment during Fmoc deprotection, standard UV

titration of the fluorenylmethyl-piperidine adduct after Fmoc deprotection is useless unless using a prior treatment for selective ester cleavage before piperidine treatment. Ester cleavage mixture was prepared as follows: 1.25 g (1.80 mmol) of $NH_2OH·HCl$ and 0.92 g (1.35 mmol) of imidazole were suspended in 5 mL of NMP and the mixture was sonicated until complete dissolution. This solution can be stored for few months at -20 °C. 5 volumes of this solution is diluted with 1 volume of DCM prior to utilization, and the resin is treated with the resulting mixture for 3 × 20 min for quantitative ester cleavage. The fluorenylmethyl-piperidine adduct is quantified by UV spectroscopy at λ = 301 nm (ε = 7800 L mol^{-1} cm^{-1}) in order to evaluate the Fmoc SPPS elongation yield of crypto-thioester peptides.

Protocol PS5 – Peptide deprotection and cleavage from the resin: the peptide was deprotected and cleaved from the resin through a treatment with TFA/H_2O/*i*Pr_3SiH/phenol 88:5:2:5. The peptide was then precipitated by dilution into an ice-cold diethyl ether/petroleum ether 1:1 mixture, recovered by centrifugation and washed twice with diethyl ether.

Protocol PS6 –Solid phase Hmb *O*-GABAylation: the ortho-hydroxyl of the Hmb group was acylated using a freshly prepared *N*-Boc-GABA anhydride solution in NMP (0.5 mmol, 0.20 g, 0.5 M), in the presence of DMAP (0.005 mmol, 0.61 mg) and DIEA (0.5 mmol, 0.065 g, 0.09 mL) for 2 h. The reaction vessel was drained, and the peptide-resin was thoroughly washed with NMP (3 × 5 mL, 30 s).

Protocol PS7 –Solid phase Hmb *O*-acetylation: the ortho-hydroxyl of the Hmb group was acylated using a freshly prepared solution of acetic anhydride in NMP (0.1 mmol, 0.010 g, 0.1 M) and DIEA (0.5 mmol, 0.065 g, 0.09 mL) for 1.5 h. The reaction vessel was drained, and the peptide-resin was thoroughly washed with NMP (3 × 5 mL, 30 s).

Protocol PS8 –Solution phase Hmb de-GABAylation: to remove the GABA group on the ortho-hydroxyl group of the Hmb, peptides were solubilized in a solution of PBS-2X/MeCN (8:2) or 6 M Gn·HCl /PBS-10X/MeCN (65/20/25) then were incubated during 30 min. Afterwards the mixture was neutralized with 1% *v*/*v* AcOH.

Protocol PS9 –General procedure for native chemical ligation:

<u>Preparation of the NCL buffer</u> (100 mM MPAA, 50 mM TCEP, 6 M guanidinium chloride, 200 mM sodium phosphate, apparent pH = 6.5, corrected pH[3] = 7.2):

A 200 mM disodium hydrogen phosphate, 6 M guanidinium chloride aqueous solution was prepared by dissolving 356 mg disodium hydrogen phosphate dihydrate (2 mmol) and 5.73 g guanidinium chloride in water (10 mL final volume). This solution can be kept at 4°C for several months. Just before use, this solution was thoroughly deoxygenated through successive vacuum/argon sparge cycles.

16.8 mg 4-mercaptophenylacetic acid (MPAA, 0.18 mmol), 14.3 mg *tris*-carboxyethylphosphine hydrochloride (TCEP, 0.057 mmol) and 10.3 mg of dry NaOH powder (0.26 mmol) were weighted into a vial, which was sealed with a septum and purged with an argon flow.

Under argon, 1 mL of this solution was added to the MPAA/TCEP/NaOH vial, followed by sonication upon complete dissolution to give the NCL buffer which was immediately used.

<u>NCL reaction</u>: The quantities of cysteinyl and crypto-thioester peptide segments were estimated by UV spectrophotometry at λ = 280 nm. The two lyophilized powders were pooled in a centrifuge tube, which was sealed with a septum and purged with an argon flow. The volume of the NCL buffer appropriate to reach the desired final peptide segments concentration was added under argon, the reaction vessel was sealed with Parafilm, and the resulting yellow solution was allowed to stir at 37 °C.

After completion, the reaction was quenched by dilution into 10 volumes of a 6 M Gn·HCl solution containing 1% *v*/*v* AcOH.

Protocol PS10 –General procedure for Hmb deprotection: the Hmb-containing peptide was deprotected through a treatment with TFA/H_2O/*i*Pr_3SiH/3,6-dioxa-1,8-octanedithiol (DODT)/phenol 83:5:2:5:5 for 1.5 h. The peptide was then precipitated by dilution into an ice-cold diethyl ether/petroleum ether 1:1 mixture, recovered by centrifugation and washed twice with diethyl ether.

Protocol PS11 –General procedure for model peptide incubation and monitoring of formation of aspartimide and related byproducts: 0.2 µmol of peptide was dissolved in 200 µL of various buffers (1 mM peptide concentration) and was incubated at the specified temperature for 196 h. The reaction course was followed by analytical HPLC using the optimized analytical gradient (Jupiter C4, 16-21% over 30 min at 20 °C), and the relative ratio between starting peptide and byproducts was determined by HPLC integration of the peaks at λ = 280 nm. In the case of buffers containing MPAA, the reaction was conducted under argon using deoxygenated buffers.

3 Incubation of SUMO-2 protein (3) under physiological conditions.

Aspartimide-containing SUMO-2 protein (**3**), which had been synthesized using the previously reported conditions not making use of *N*-Hmb groups,[4] was subjected to incubation at a concentration of 2 mM in PBS at a pH of 7.4, maintained at a temperature of 37 °C for 44 h. The rate of M-18 Da byproducts was determined by integrating the peaks corresponding to M and M-18 of the multicharged ions $[M+9H]^{9+}$, chosen as the most abundant multicharged ion enabling resolution of the two peaks. It was observed that the M-18 rate decreased from 6% to 3.5%.

b. Protein **3** before incubation.

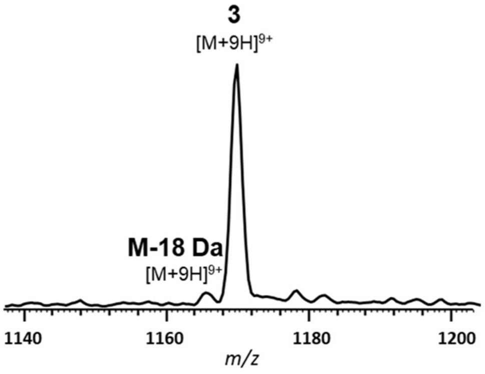

a. Protein **3** after incubation.

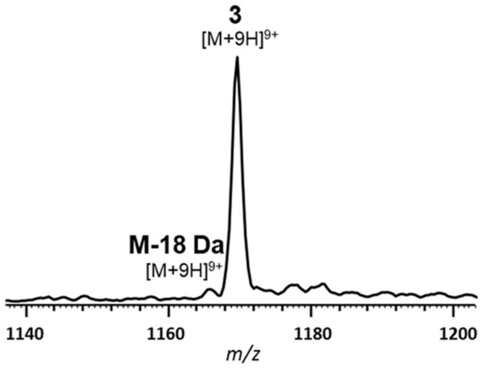

Supplementary figure S01: Mass spectra of the $[M+9H]^{9+}$ ion for the protein mixture incubated under mimicking physiological conditions.

4 Incubation of SUMO-2 segments under NCL conditions.

SUMO-2[1-47] cryptothioester **1** and Cys(K_6-Ades)-SUMO-2[49-93]-OH (**2a**) were synthesized as described in previous report.[4] These segments were incubated separately at 2 mM in NCL buffer.

To prevent the formation of numerous byproducts from the cryptothioester (*N*-Hnb-Cys) device in segment **1**, excess cysteine (10 mM) was added during the incubation experiment to form the NCL product **4**.

Asi formation was monitored using LC-MS. The rate of M-18 byproducts formation was determined by integrating the peaks corresponding to M and M-18 of the multicharged ions chosen as the most abundant multicharged ion enabling resolution of the two peaks. In both cases, a ≈11% rate of M-byproducts was observed.

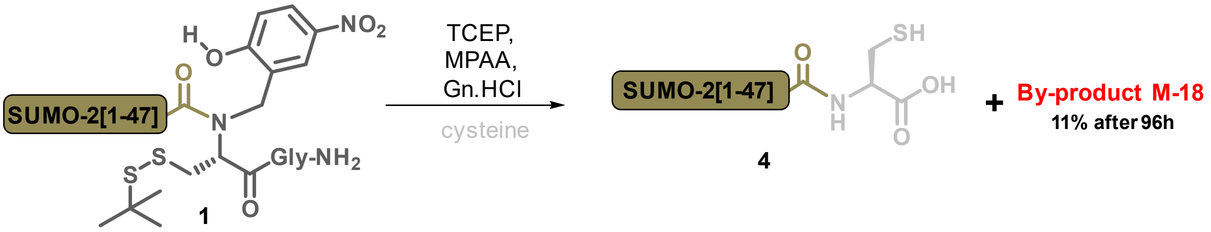

a. Before incubation.

b. After 96h incubation.

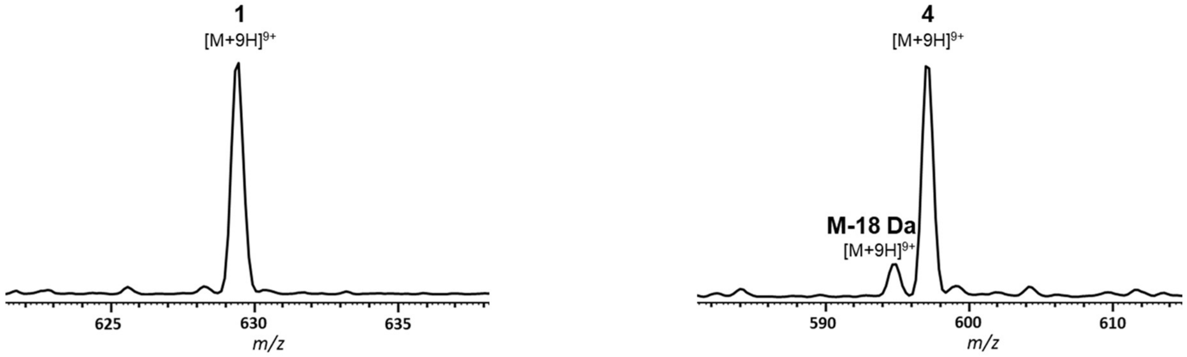

Supplementary figure S02: Comparative mass spectra of the N-terminal segment of SUMO-2: before and after incubation under NCL conditions (zoom on the $[M+9H]^{9+}$ peaks).

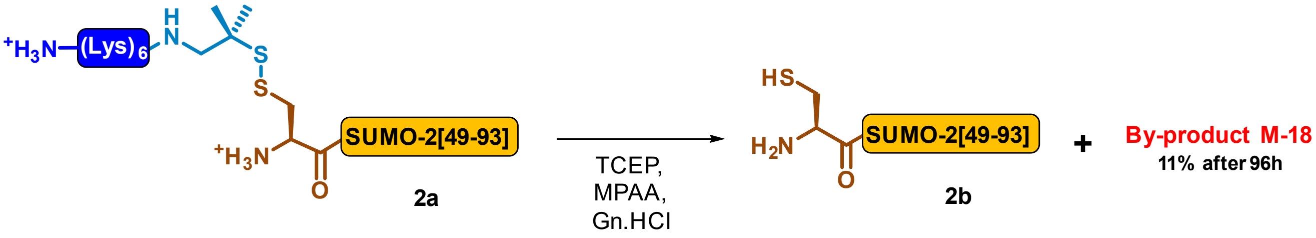

a. Before incubation.

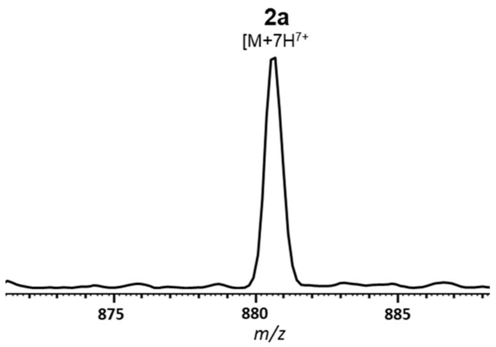

b. After 96h incubation.

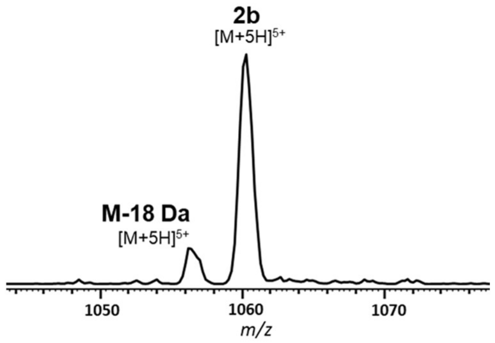

Supplementary figure S03: Comparative mass spectra of the C-terminal segment of SUMO-2: before and after incubation under NCL conditions (zoom on the $[M+7H]^{7+}$(**2a**) and $[M+5H]^{5+}$ (**2b**) peaks).

5 Synthesis of HPLC standards to monitor Asi and related byproducts formation on a model peptide sequence

5.1 Synthesis of native model peptide

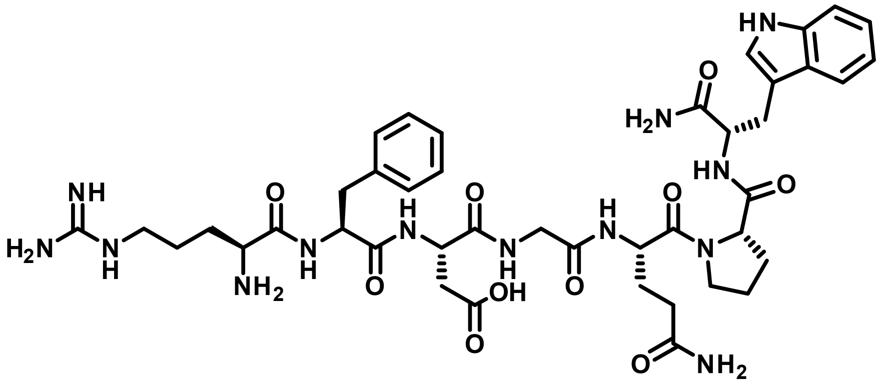

H-Arg-Phe-Asp-Gly-Gln-Pro-Trp-NH_2 (**5**)

Peptide **5** was synthesized on a Rink amide MBHA polystyrene resin (0.68 mmol/g) following protocol PS1. Cleavage was performed for 2 h following protocol PS5. The crude peptide was purified by preparative HPLC.

ESI-MS (*m/z*): [M+H] calcd. for $C_{42}H_{58}N_{13}O_{10}^{+}$: 904.4, found: 904.4 (monoisotopic mass).

HPLC analysis: t_R = 3.36 min (Chromolith, gradient: 5-50% B over 5 min).

HPLC purification: [5 mg/mL] Nucleosil C18, gradient: 15-45% B over 60 min, 34%.

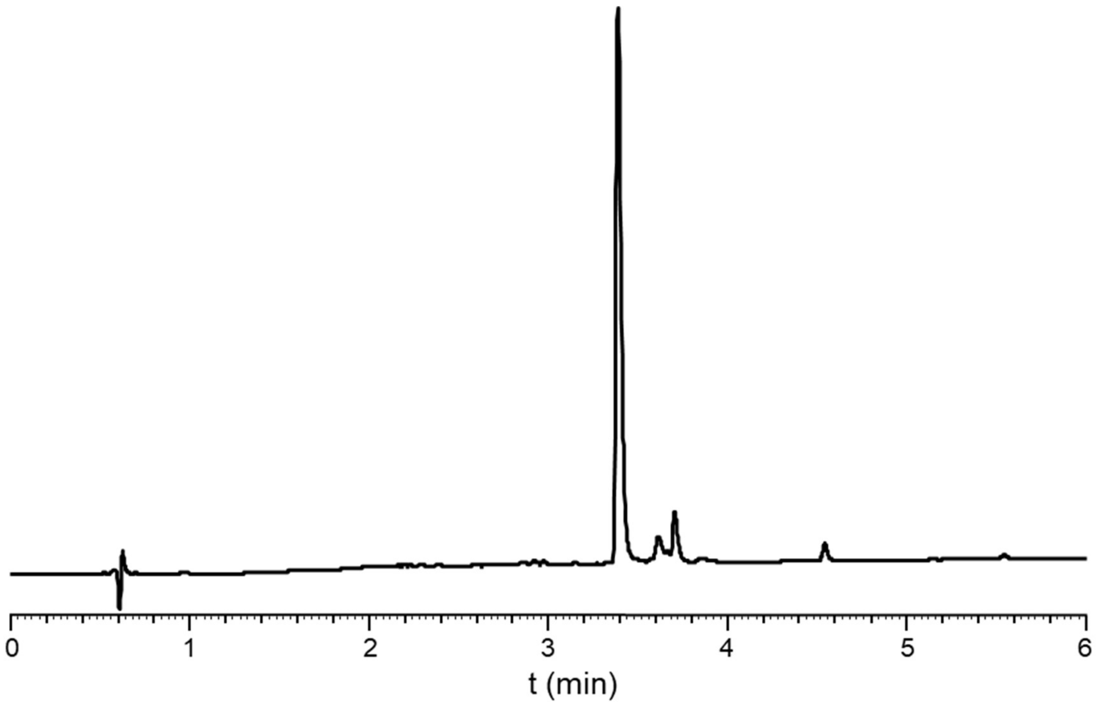

Supplementary figure S04: HPLC trace of crude **5** (λ = 214 nm).

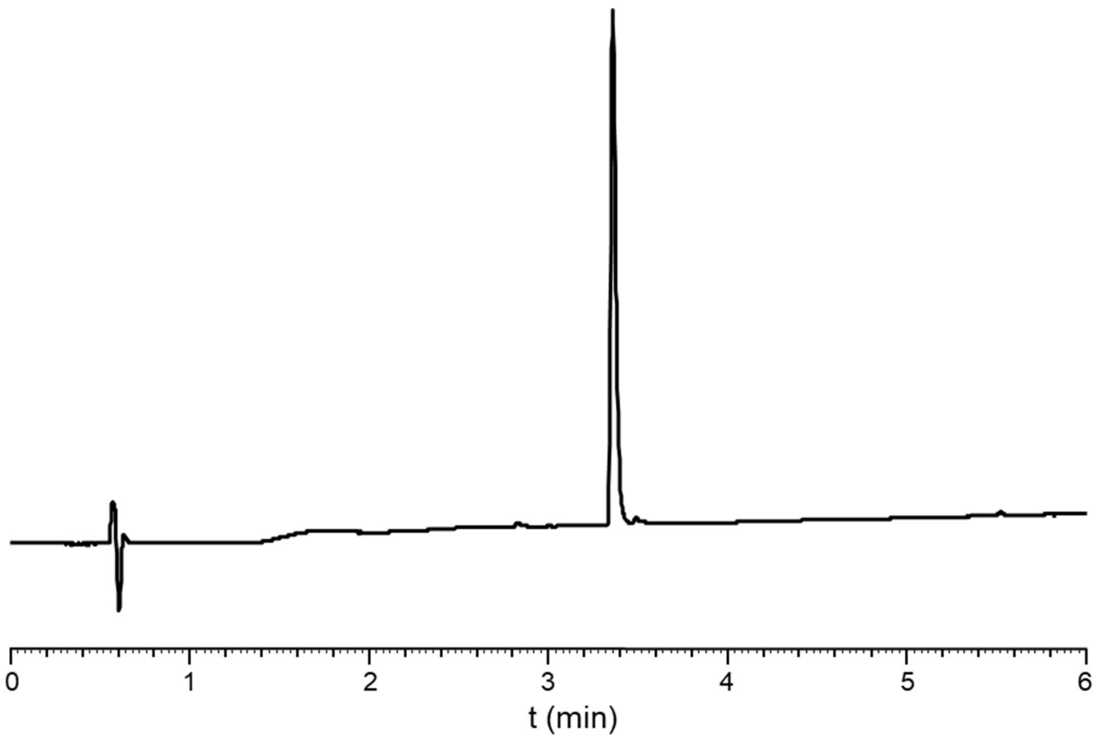

Supplementary figure S05: HPLC trace of purified **5** (λ = 214 nm).

5.2 Synthesis of L-aspartimide (L-Asi) and L-*iso*Asp-containing peptides

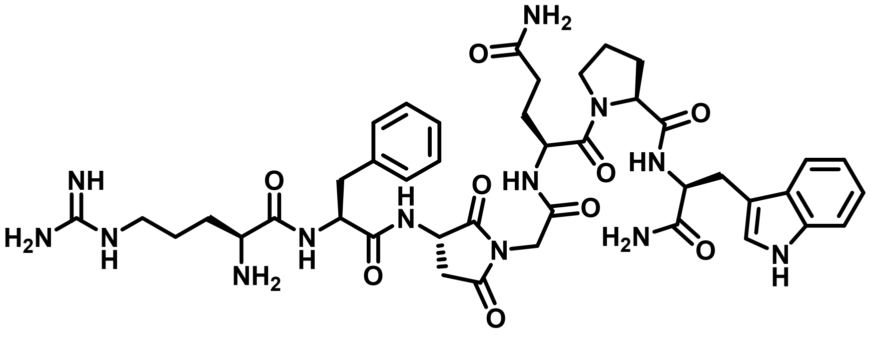

H-Arg-Phe-Asi-Gly-Gln-Pro-Trp-NH_2 (**6**)

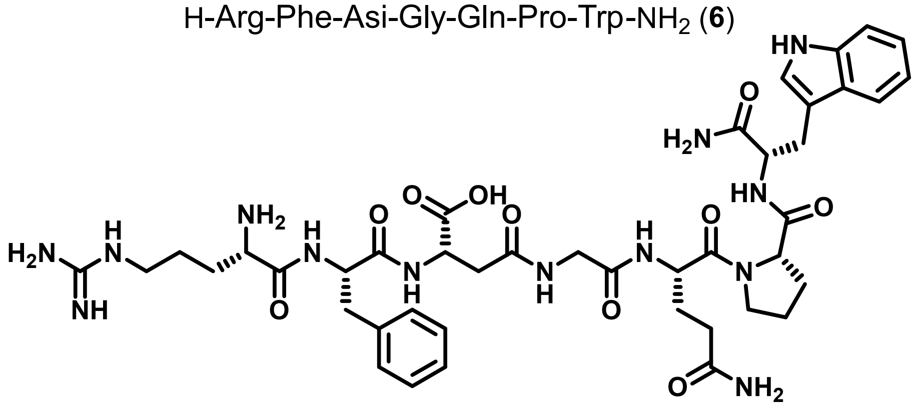

H-Arg-Phe-*iso*Asp-Gly-Gln-Pro-Trp-NH_2 (**7**)

Peptide **7** was synthesized on a Rink amide MBHA polystyrene resin (0.68 mmol/g) following protocol PS1, using Fmoc-Asp-O*t*Bu as building block to introduce the *iso*Asp residue, leading to a mixture of the desired peptide **7** and L-Asi-containing peptide **6** formed as a co-product during the elongation. Cleavage was performed for 2 h following protocol PS5. The crude mixture was purified by preparative HPLC.

6: L-Asi-containing peptide

ESI-MS (*m/z*): [M+H] calcd. for $C_{42}H_{56}N_{13}O_9^+$: 886.4, found: 886.4 (monoisotopic mass).

HPLC analysis: t_R = 3.58 min (Chromolith, gradient: 5-50% B/A over 5 min).

HPLC purification: [5 mg/mL] Nucleosil C18, gradient: 15-45% B/A over 60 min, 8 %.

7: L-*iso*Asp-containing peptide

ESI-MS (*m/z*): [M+H] calcd. for $C_{42}H_{58}N_{13}O_{10}^+$: 904.4, found: 904.4 (monoisotopic mass).

HPLC analysis: t_R = 3.33 min (Chromolith, gradient: 5-50% B/A over 5 min).

HPLC purification: [5 mg/mL] Nucleosil C18, gradient: 15-45% B/A over 60 min, 29%.

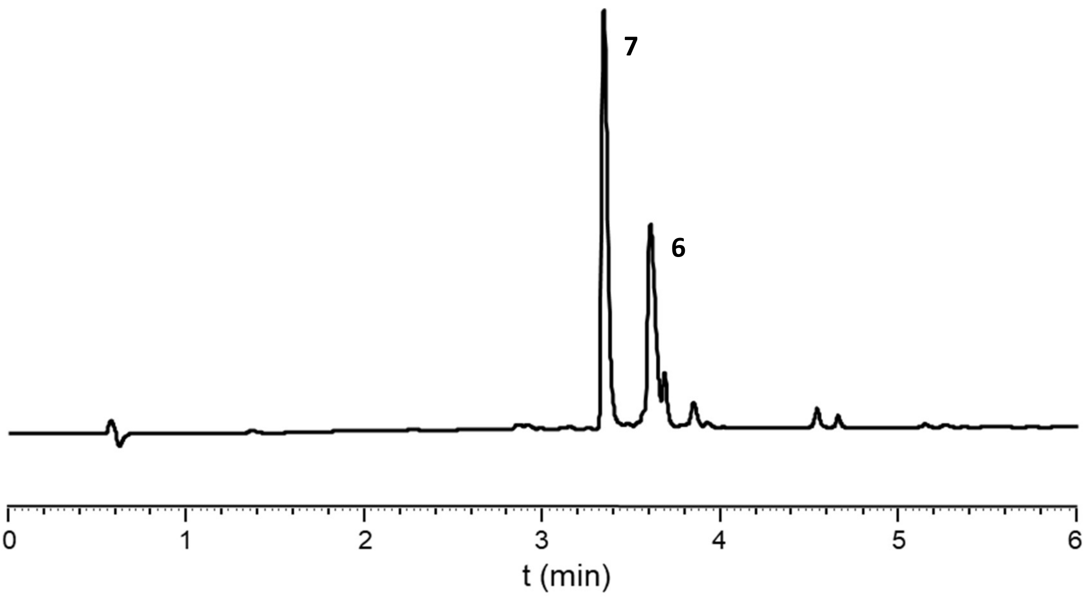

Supplementary figure S06: HPLC trace of crude **6** and **7** (λ = 214 nm).

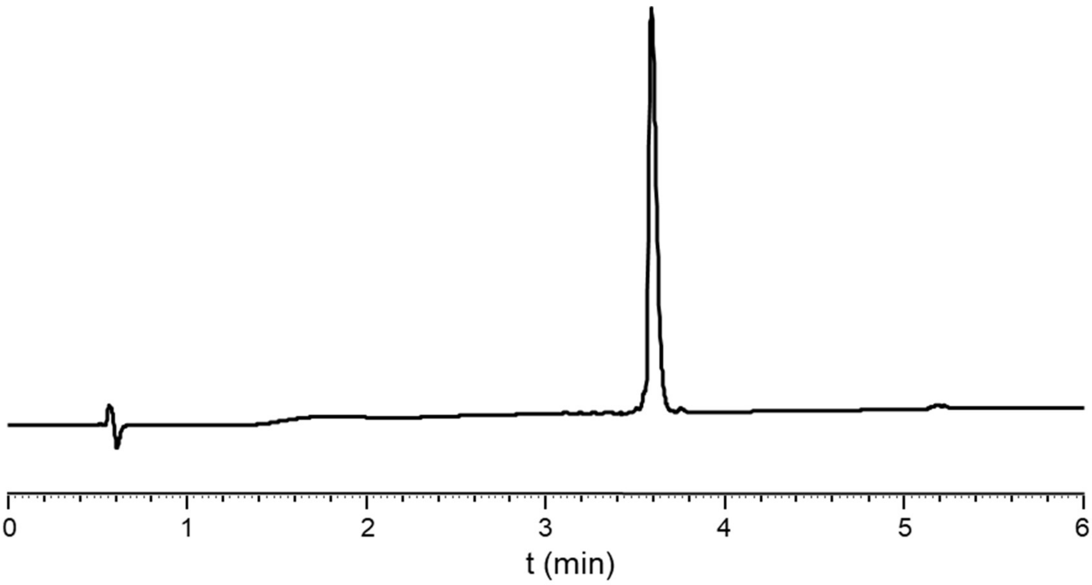

Supplementary figure S07: HPLC trace of purified **6** (λ = 214 nm).

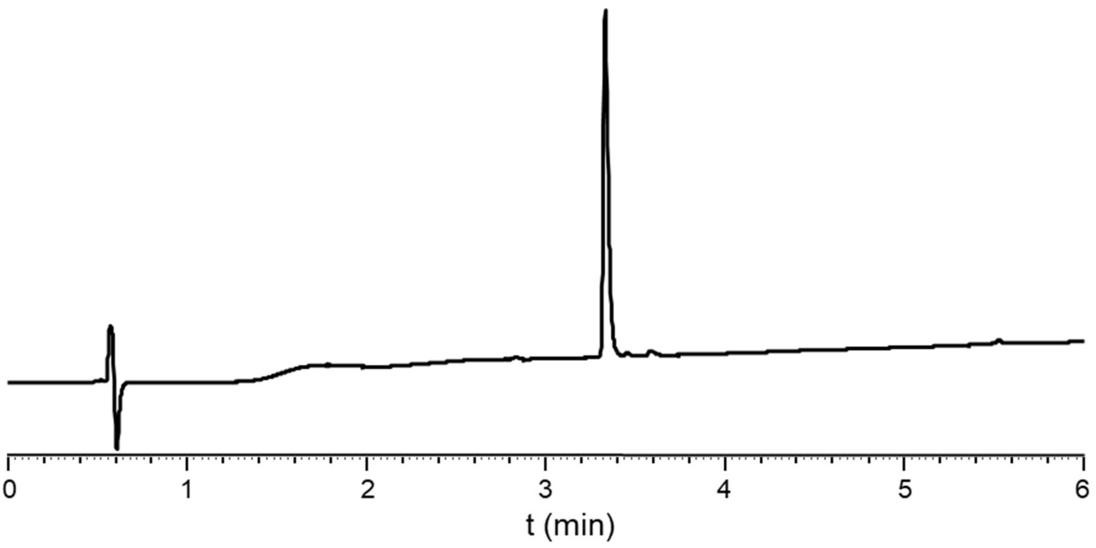

Supplementary figure S08: HPLC trace of purified **7** (λ = 214 nm).

5.3 Synthesis of D-Asp-containing model peptide (8)

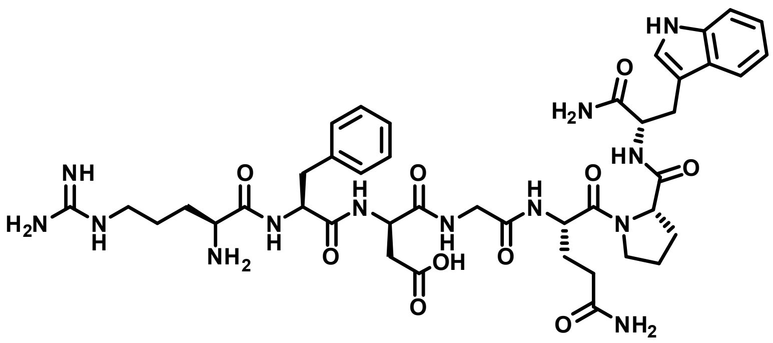

H-Arg-Phe-D-Asp-Gly-Gln-Pro-Trp-NH_2 (**8**)

Peptide **8** was synthesized on a Rink amide MBHA polystyrene resin (0.68 mmol/g) following protocol PS1, using Fmoc-D-Asp(O*t*Bu) as building block to introduce the D-Asp residue. Cleavage was performed for 2 h following protocol PS5. The crude was purified by preparative HPLC.

ESI-MS (*m/z*): [M+H] calcd. for $C_{42}H_{58}N_{13}O_{10}^{+}$: 904.4, found: 904.4 (monoisotopic mass).

HPLC analysis: t_R = 3.39 min (Chromolith, gradient: 5-50% B/A over 5 min).

HPLC purification: [5 mg/mL] Nucleosil C18, gradient: 15-45% B/A over 60 min, 62%.

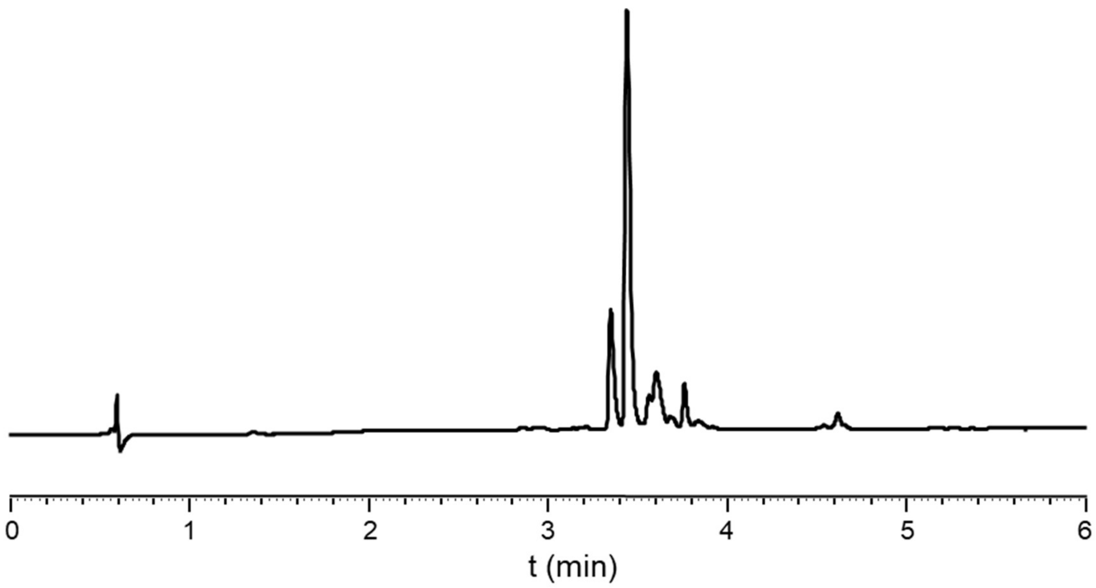

<u>Supplementary figure S09</u>: HPLC trace of crude **8** (λ = 214 nm).

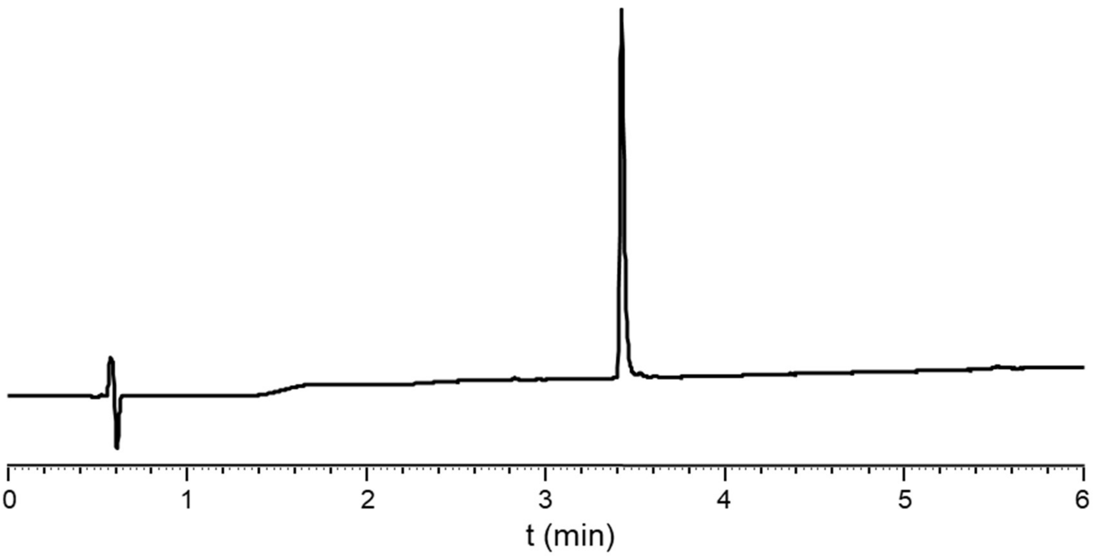

<u>Supplementary figure S09</u>: HPLC trace of purified **8** (λ = 214 nm).

5.4 Synthesis of D-Asi- and D-*iso*Asp-containing peptides (9 & 10)

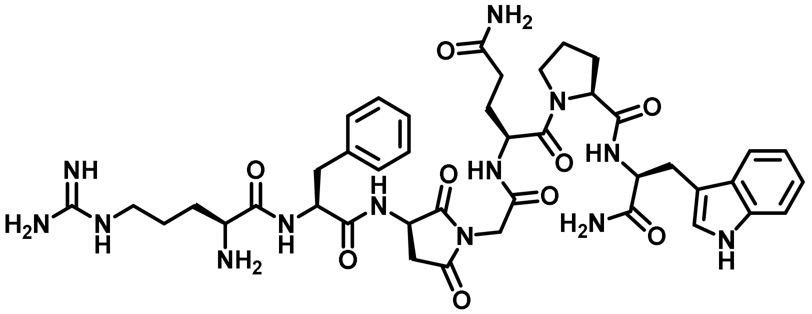

H-Arg-Phe-D-Asi-Gly-Gln-Pro-Trp-NH_2 (**9**)

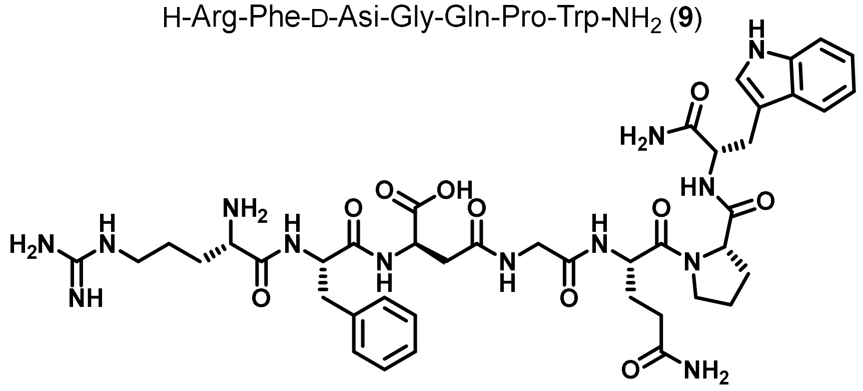

H-Arg-Phe-D-*iso*Asp-Gly-Gln-Pro-Trp-NH_2 (**10**)

Peptide **10** was synthesized on a Rink amide MBHA polystyrene resin (0.68 mmol/g) following protocol PS1, using Fmoc-D-Asp-O*t*Bu as building block to introduce the D-*iso*Asp residue, leading to a mixture of **10** and D-Asi-containing peptide **9** formed as a co-product during the elongation. Cleavage was performed for 2 h following protocol PS5. The crude mixture was purified by preparative HPLC.

9: D-Asi-containing peptide

ESI-MS (*m/z*): [M+H] calcd. for $C_{42}H_{56}N_{13}O_9^+$: 886.4, found: 886.4 (monoisotopic mass).

HPLC analysis: t_R = 3.54 min (Chromolith, gradient: 5-50% B/A over 5 min).

HPLC purification: [5 mg/mL] Nucleosil C18, gradient: 15-45% B/A over 60 min, 17 %.

10: D-*iso*Asp-containing peptide

ESI-MS (*m/z*): [M+H] calcd. for $C_{42}H_{58}N_{13}O_{10}^+$: 904.4, found: 904.4 (monoisotopic mass).

HPLC analysis: t_R = 3.30 min (Chromolith, gradient: 5-50% B/A over 5 min).

HPLC purification: [5 mg/mL] Nucleosil C18, gradient: 15-45% B/A over 60 min, 25%.

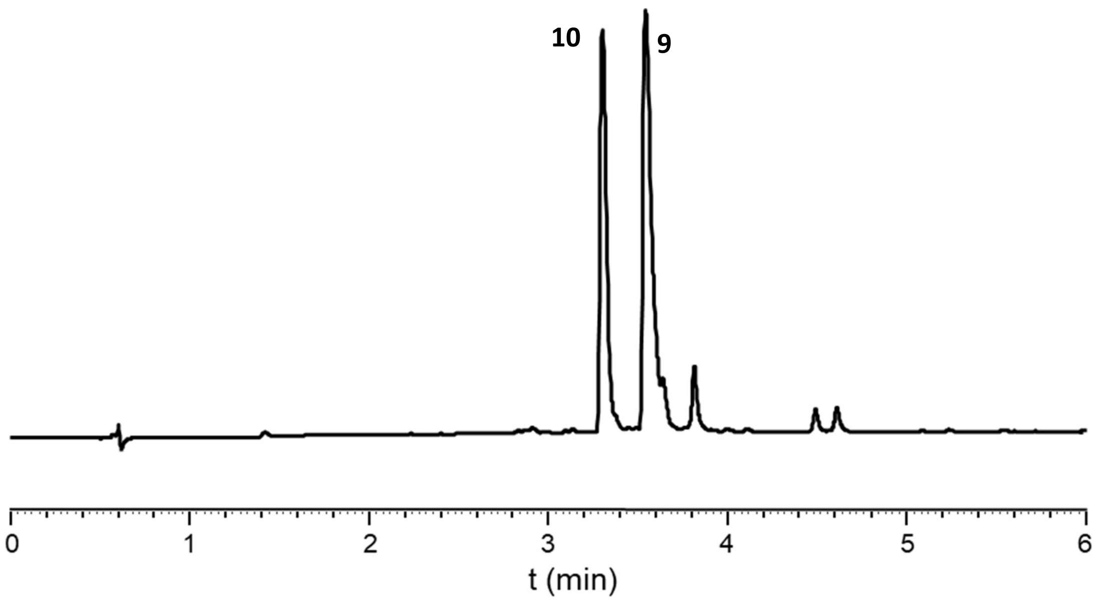

Supplementary figure S10: HPLC trace of crude **9** and **10** (λ = 214 nm).

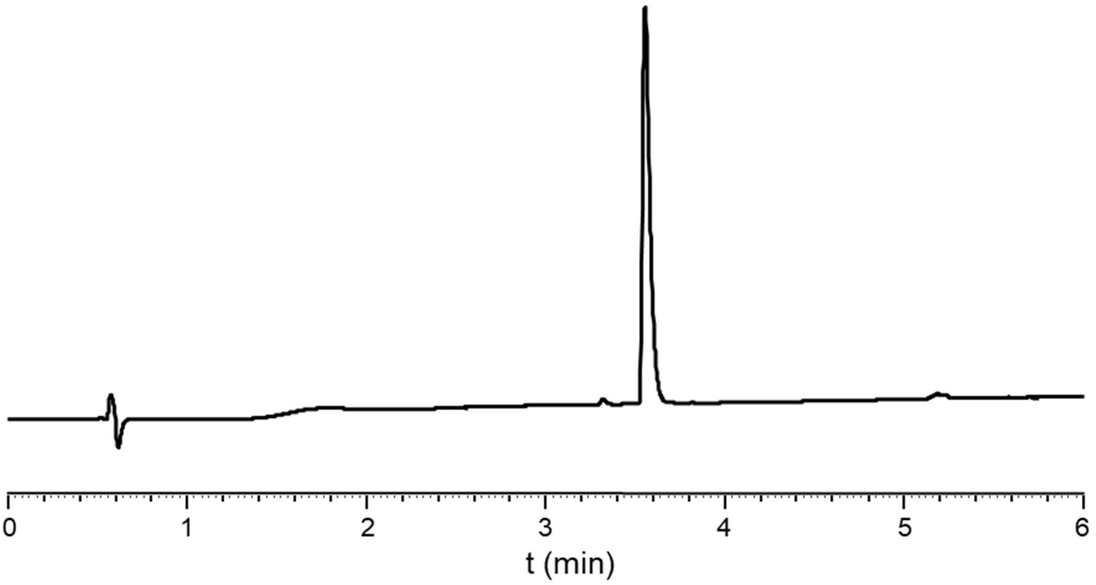

Supplementary figure S11: HPLC trace of purified **9** (λ = 214 nm).

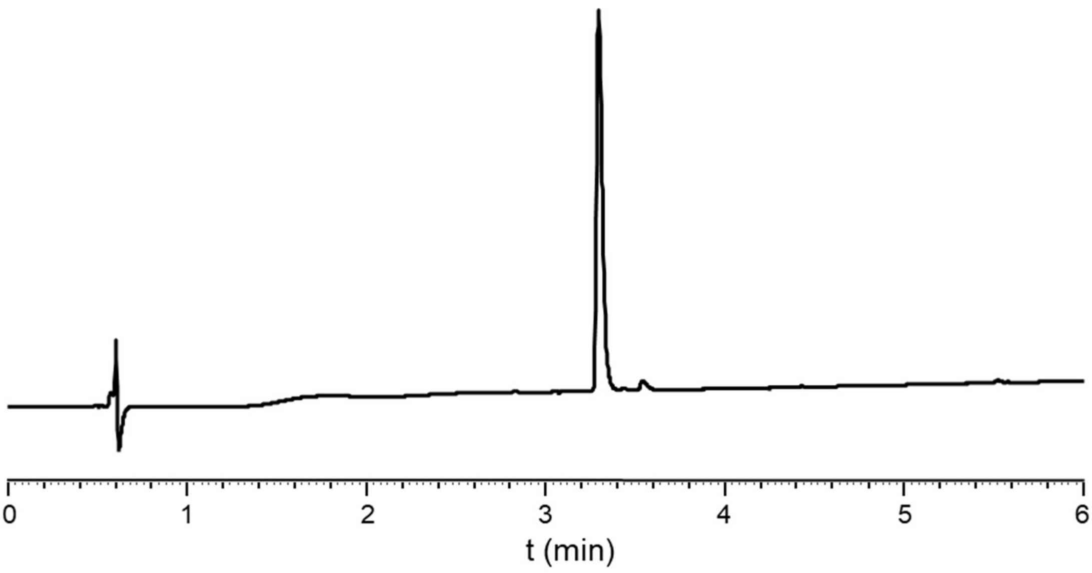

Supplementary figure S12: HPLC trace of purified **10** (λ = 214 nm).

5.5 Synthesis of Asn-containing peptide (S01)

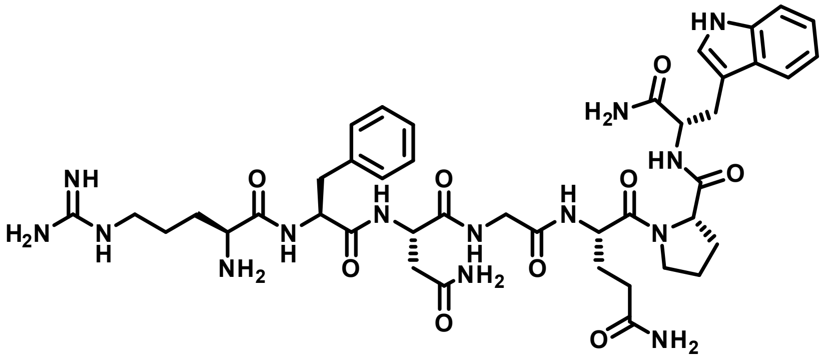

H-Arg-Phe-Asn-Gly-Gln-Pro-Trp-NH_2 (**S01**)

Peptide **S01** was synthesized on a Rink amide MBHA polystyrene resin (0.68 mmol/g) following protocol PS1. Cleavage was performed for 2 h following protocol PS5. The crude was purified by preparative HPLC.

ESI-MS (*m/z*): [M+H] calcd. for $C_{42}H_{59}N_{14}O_9^+$: 903.5, found: 903.5 (monoisotopic mass).

HPLC analysis: t_R = 3.31 min (Chromolith, gradient: 5-50% B/A over 5 min).

HPLC purification: [5 mg/mL] Nucleosil C18, gradient: 15-45% B/A over 60 min, 46%.

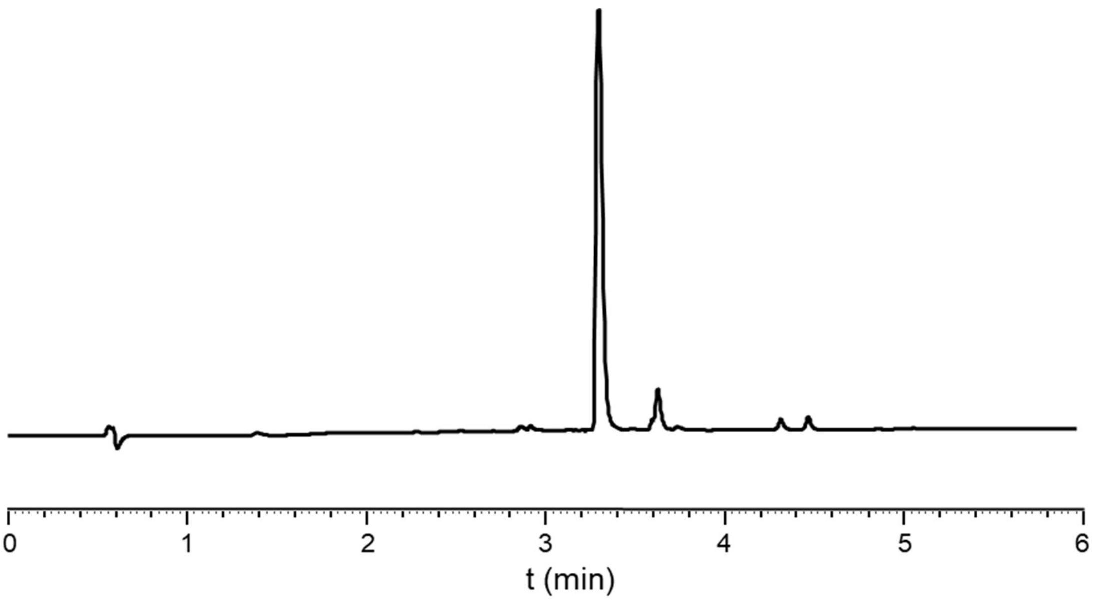

Supplementary figure S13: HPLC trace of crude **S01** (λ = 214 nm).

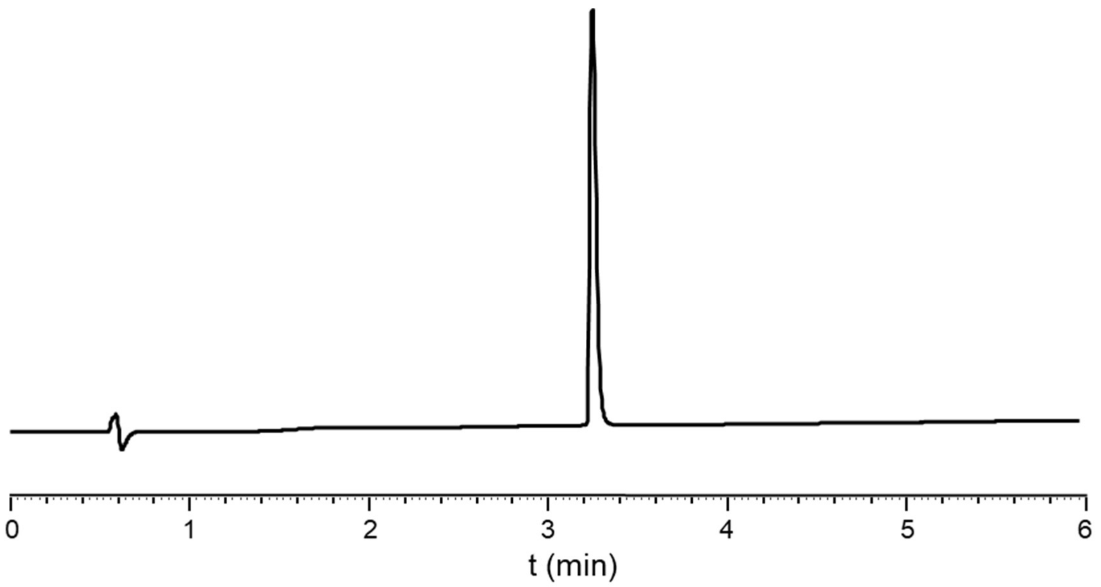

Supplementary figure S14: HPLC trace of purified **S01** (λ = 214 nm).

6 Separation of the model peptide and analytical standards by HPLC.

An equimolar mixture of model peptide **5** and the 5 analytical standards (**6**-**10**) was prepared, and an analytical HPLC gradient was optimized to allow a clear separation of the six compounds (Jupiter C4, 16-21% over 30 min at 20 °C).

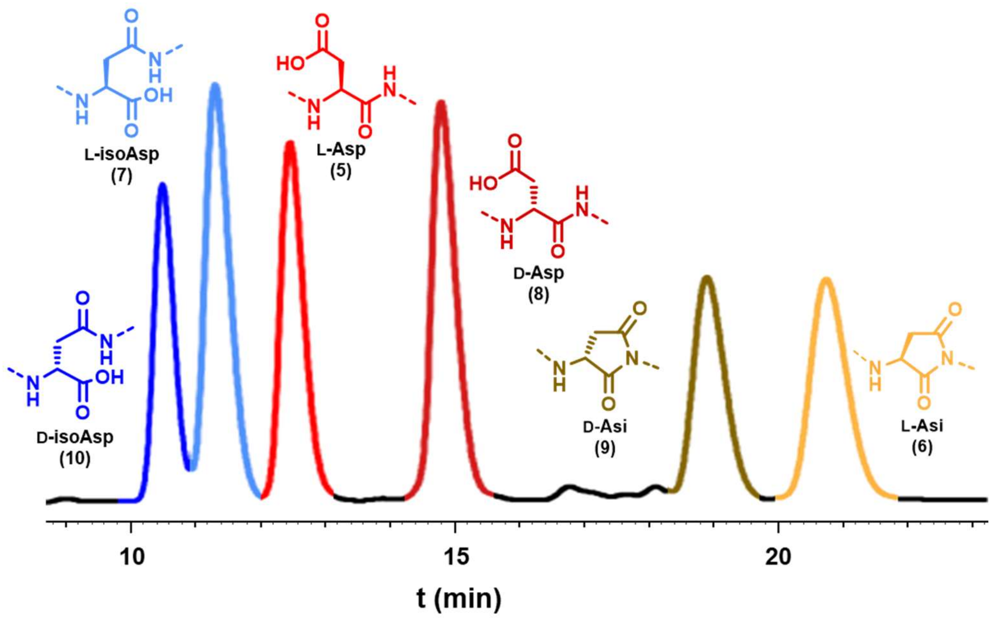

Supplementary figure S15: HPLC trace of separation of a standard mix (λ = 280 nm).

7 Monitoring of Asi hydrolysis and epimerization

To confirm the decrease in the M-18 product suspected to be Asi during the incubation of protein **3** and thereby offer an initial characterization of this byproduct, we decided to incubate L-Asi-containing peptide (**6**), under conditions similar to those used for **3** (PBS buffer during 48 h). The stability of Asi was monitored by HPLC (Supplementary table, entry 1). Subsequently, this experiment was expanded by incubating peptide **6** under NCL conditions (buffer preparation following protocol PS9) following PS11 protocol at 37 °C (Supplementary table entry 2).

Supplementary table S01: Estimation of products obtained from the incubation of Asi containing peptide **6**.[a]

Entry	Buffer	Time (h)	L-Asi (**6**) (%) *(Starting compound)*	D-*iso*Asp (**10**) (%)	L-*iso*Asp (**7**) (%)	L-Asp (**5**) (%)	D-Asp (**8**) (%)	D-Asi (**9**) (%)
**1**	PBS	24	5.0	3.0	56.2	16.4	3.8	0.6
		48	0.9	6.0	69.1	19.5	4.5	-
**2**	NCL	16	63.7	1.5	16.0	7.4	1.8	8.4
		48	31.9	1.9	46.5	15.2	2.4	1.9

a " – " sign means that the compound was either not detected or the peak not possible to integrate precisely due to very small amounts or elution close to a contaminant peak.

8 Monitoring of Asi formation during various NCL condition incubation

8.1 Incubation of model peptide 5 under NCL conditions

Following the procedure PS11, model peptide **5** was incubated in NCL buffer (buffer preparation following protocol PS9) at 50 °C and monitored by HPLC.

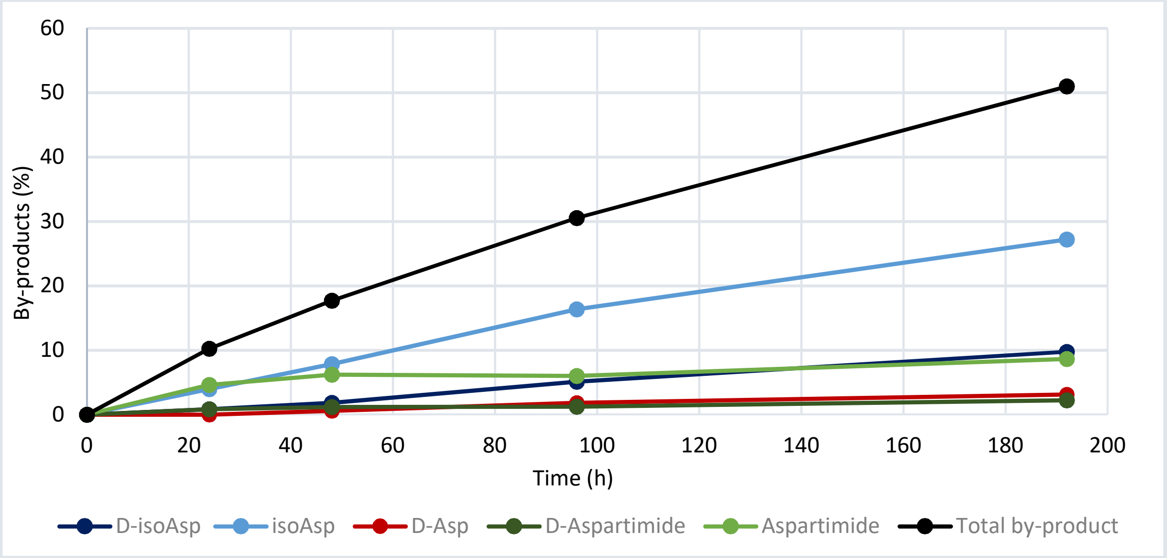

Supplementary figure S16: Kinetic monitoring of Asi (**6**) and related byproducts (**7-10**) formation upon incubation of **5** under NCL conditions at 50 °C.

8.2 Incubation of NG-containing model peptide S01 under NCL conditions

The incubation of containing NG motif peptide **S01** was carried out following the same protocol as for peptide **5**. We also observed the formation of Asi and its related byproducts. However, under the separation conditions used, the isoAsp byproducts **7** had a retention time nearly identical to the one of peptide **S01**. As the opening of Asi typically leads to the formation of isoAsp and Asp in a 3:1 ratio, we assumed this ratio to estimate the rate of isoAsp formation. In the case of L-isoAsp, the estimate was only done for 24 h and 48 h, because for 96 h and 192 h, the L-isoAsp peak was easily distinguishable from the **S01** peak.

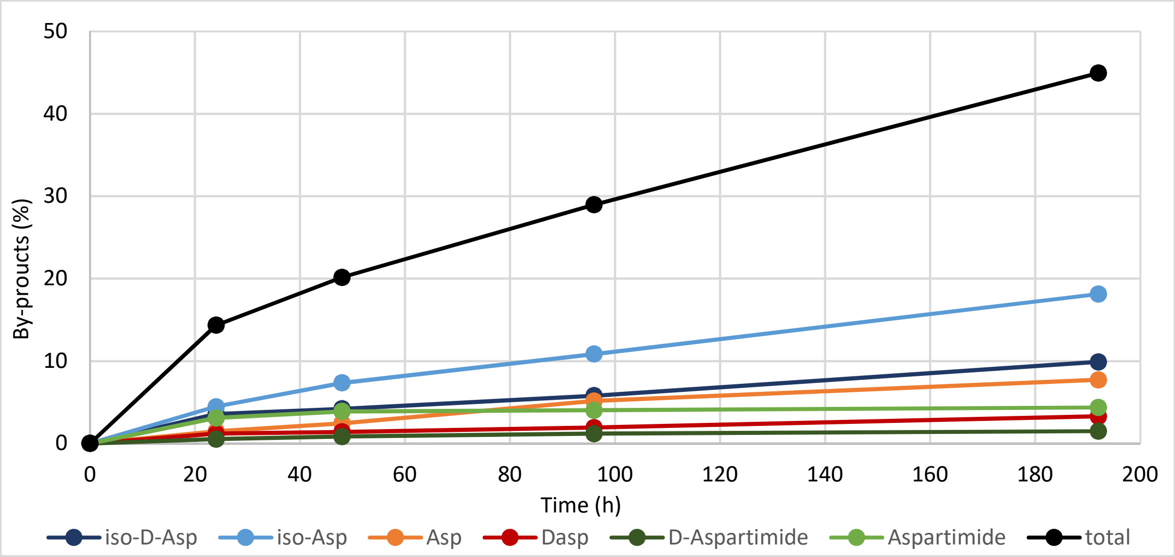

Supplementary figure S17: Kinetic monitoring of Asi (**6**) and related byproducts (**7-10**) formation upon incubation of NG-containing model peptide **S01** under NCL conditions at 50 °C.

8.3 Influence of the buffer on the rate of formation of Asi and related byproducts.

In order to determine if some constituents of the NCL buffer (Gu·HCl, phosphate, MPAA, TCEP) were responsible for an enhanced formation of Asi and related by products, we systematically compared our NCL buffers with ones where one or several components was omitted (or replaced by HEPES in the case of phosphate), following protocol PS11 at 50°C.

a. Buffers preparation

NCL buffer: See general protocol PS9.

Phosphate + Gu·HCl: A solution containing 6 M Gn·HCl and 200 mM sodium phosphate was prepared. This solution was adjusted at apparent pH = 6.5 (corrected pH[3] = 7.2).

Phosphate: A 200 mM sodium phosphate solution was prepared. This solution was adjusted at pH 7.2.

HEPES: A 200 mM HEPES solution was prepared. This solution was adjusted at pH = 7.2.

HEPES + Gu·HCl: A solution containing 6 M Gn·HCl and 200 mM HEPES was prepared. This solution was adjusted at apparent pH = 6.5 (corrected pH[3] = 7.2).

b. Results

Supplementary table S02: Influence of the buffer on Asi **6** and related byproducts **7-10** formation after 192 h at 50 °C.[a]

Entry	Buffer	Time (h)	D-*iso*Asp (**10**) (%)	L-*iso*Asp (**7**) (%)	D-Asp (**8**) (%)	D-Asi (**9**) (%)	L-Asi (**6**) (%)	Total (%)	Asi / Total byproducts (%)
**1**	NCL	24	0.8	4.0	-	0.8	4.6	10.2	53.2
		96	5.1	16.4	1.8	1.2	6.0	30.5	23.6
		192	9.8	27.2	3.1	2.2	8.6	51.0	21.3
**2**	Phosphate + Gn·HCl	24	0.9	5.0	0.2	0.2	1.8	8.1	25.1
		96	4.1	15.9	1.2	0.4	2.3	23.9	11.2
		192	7.9	26.2	3.0	0.8	3.0	40.9	9.2
**3**	Phosphate	24	0.7	6.5	-	-	-	7.2	0
		96	2.4	22.1	0.1	-	-	24.6	0
		192	5.2	34.2	2.2	-	-	41.6	0
**4**	HEPES	24	0.0	4.7	0.0	-	-	4.7	0
		96	1.8	17.7	0.0	-	-	19.4	0
		192	2.4	29.1	2.5	-	-	34.0	0
**5**	HEPES + Gn·HCl	24	-	1.5	-	-	3.0	4.5	67.3
		96	1.4	10.0	-	-	4.3	15.7	27.4
		192	3.2	19.7	0.9	1.3	5.0	30.2	21.0

a " – " sign means that the compound was either not detected or the peak not possible to integrate precisely due to very small amounts or elution close to a contaminant peak.

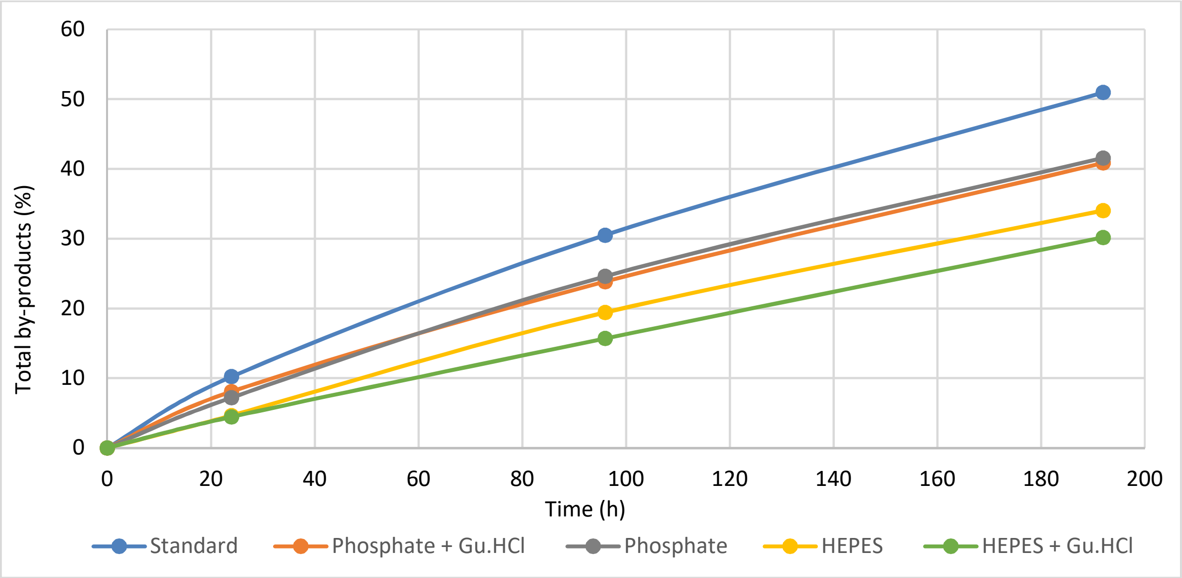

Supplementary figure S18: Influence of the buffer on total byproducts formation (sum of **6**-**10**).

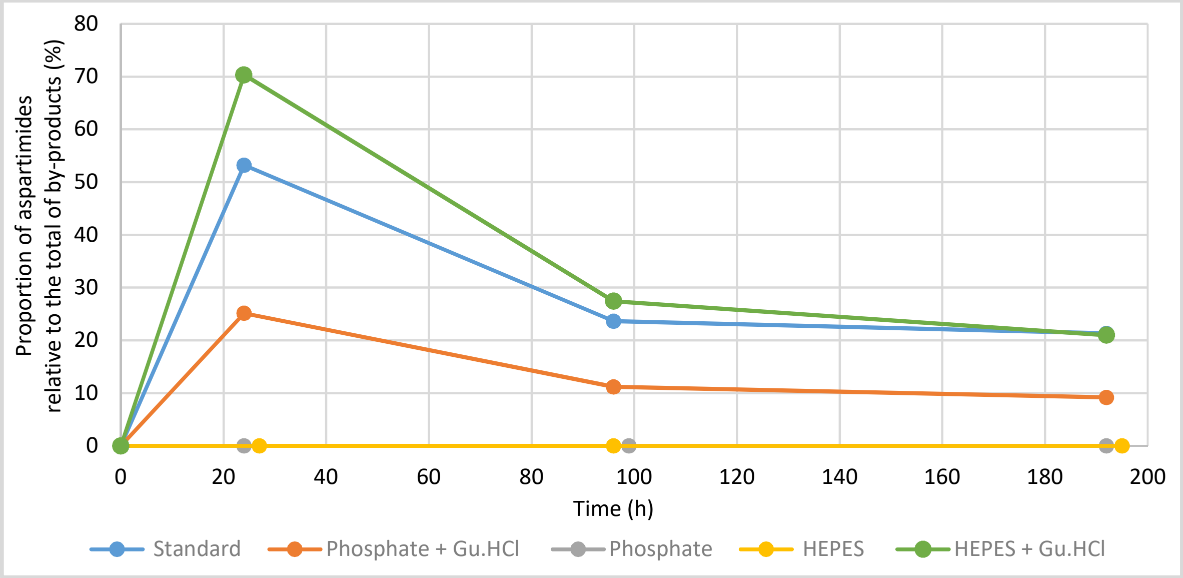

Supplementary figure S19: Influence of the buffer on total Asi formation (ratio between the sum of **6** and **9** on all byproducts).

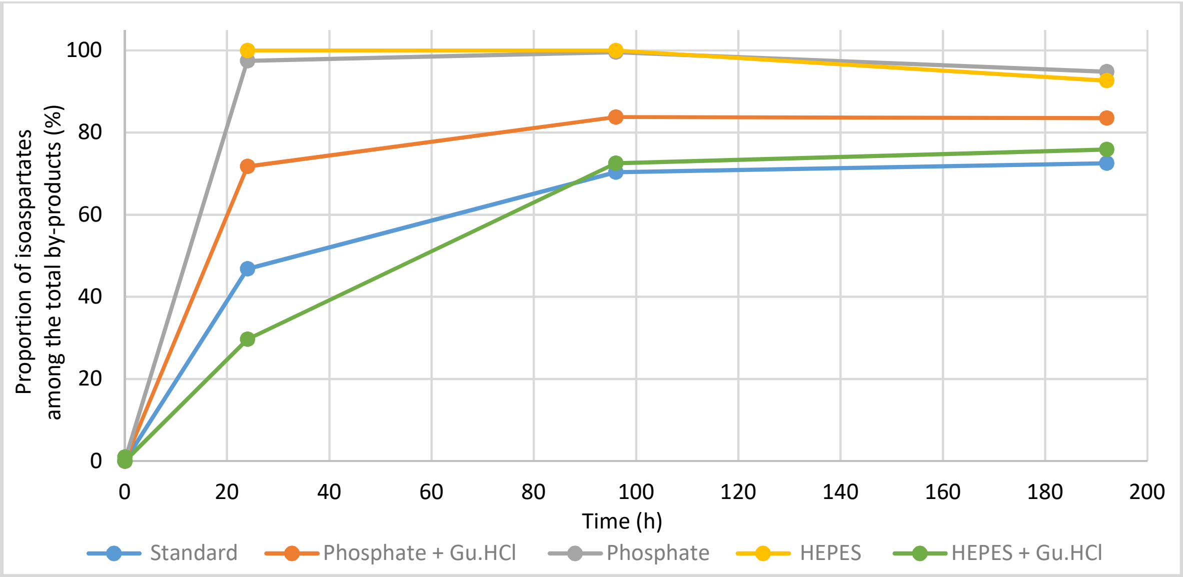

Supplementary figure S20: Influence of the buffer on the total amount of *iso*-Asp-containing byproducts (ratio between the sum of **7** and **10** on all byproducts).

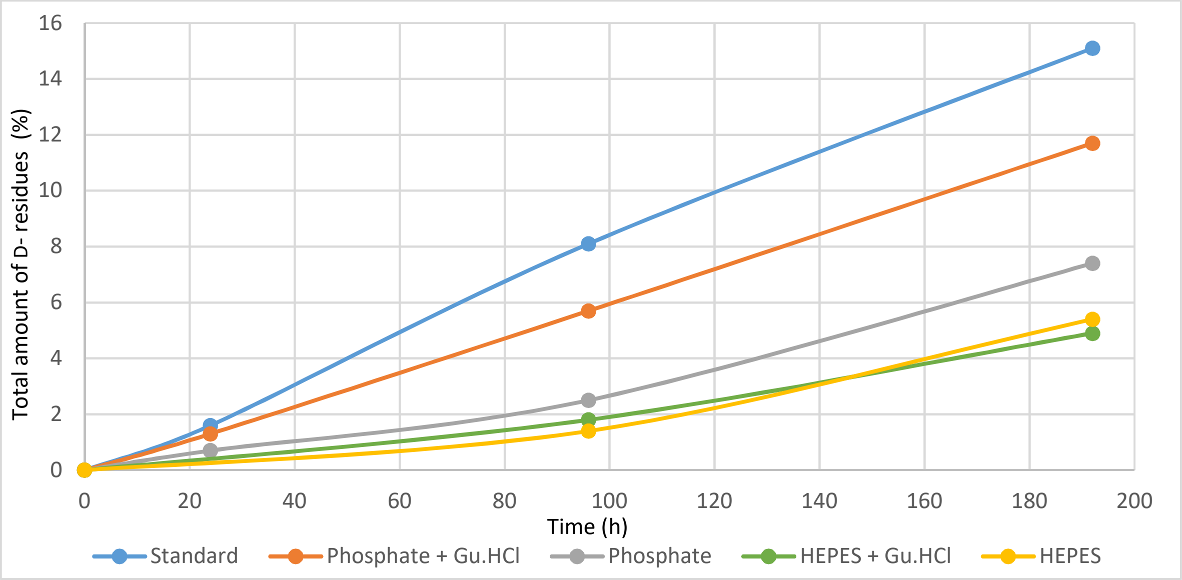

Supplementary figure S21: Influence of the buffer on the total amount of D residues-containing byproducts (**8**, **9** and **10**).

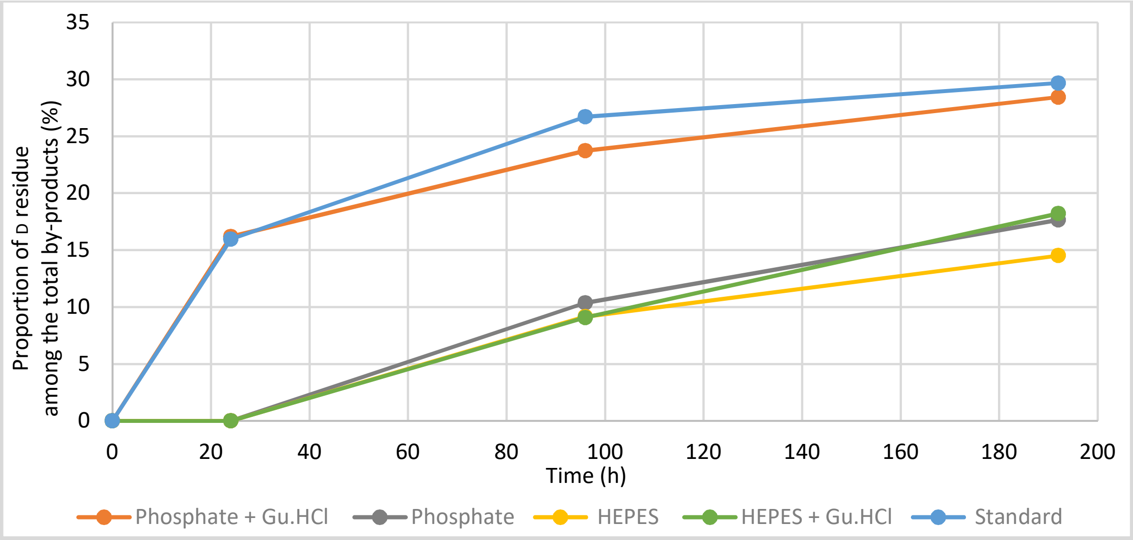

Supplementary figure S22: Influence of the buffer on the relative amount of epimerized byproducts (ratio between the sum of **8**, **9** and **10** on all byproducts).

8.4 Influence of the pH on the rate of formation of Asi and related byproducts.

We screened different pH conditions (6.2, 6.7, 7.2 & 7.4) following the procedure S11 at 50 °C. The buffer chosen for this study was phosphate + Gn·HCl.

a. Buffers preparation

Phosphate + Gn·HCl : A solution containing 6 M Gn·HCl & 200 mM sodium phosphate was prepared. This solution was adjusted at different corrected pH[3] 6.2, 6.7, 7.2 & 7.7.

b. Results:

Supplementary table S03: Influence of the pH on Asi **6** and related byproducts **7-10** formation.

Entry	pH	Time (h)	D-*iso*Asp (**10**) (%)	L-*iso*Asp (**7**) (%)	D-Asp (**8**) (%)	D-Asi (**9**) (%)	L-Asi (**6**) (%)	Total (%)	Asi / Total byproducts (%)
**1**	6.2	24	0.18	2.21	-	0.65	9.70	12.7	81.2
		96	1.97	12.80	0.96	3.12	20.03	38.9	59.6
		192	6.55	26.15	2.65	5.33	20.33	61.0	42.1
**2**	6.7	24	0.40	3.03	0.08	0.45	4.32	8.3	57.6
		96	3.39	15.34	1.52	1.09	5.65	27.0	25.0
		192	7.64	28.89	3.21	1.35	5.04	46.1	13.8
**3**	7.2	24	0.9	5.0	0.2	0.2	1.8	8.1	25.1
		96	4.1	15.9	1.2	0.4	2.3	23.9	11.2
		192	7.9	26.2	3.0	0.8	3.0	40.9	9.2
**4**	7.7	24	0.66	3.58	0.30	0.29	2.98	7.8	41.9
		96	3.20	14.94	1.28	0.46	2.20	22.1	12.0
		192	6.80	26.82	2.54	0.43	1.75	38.3	5.7

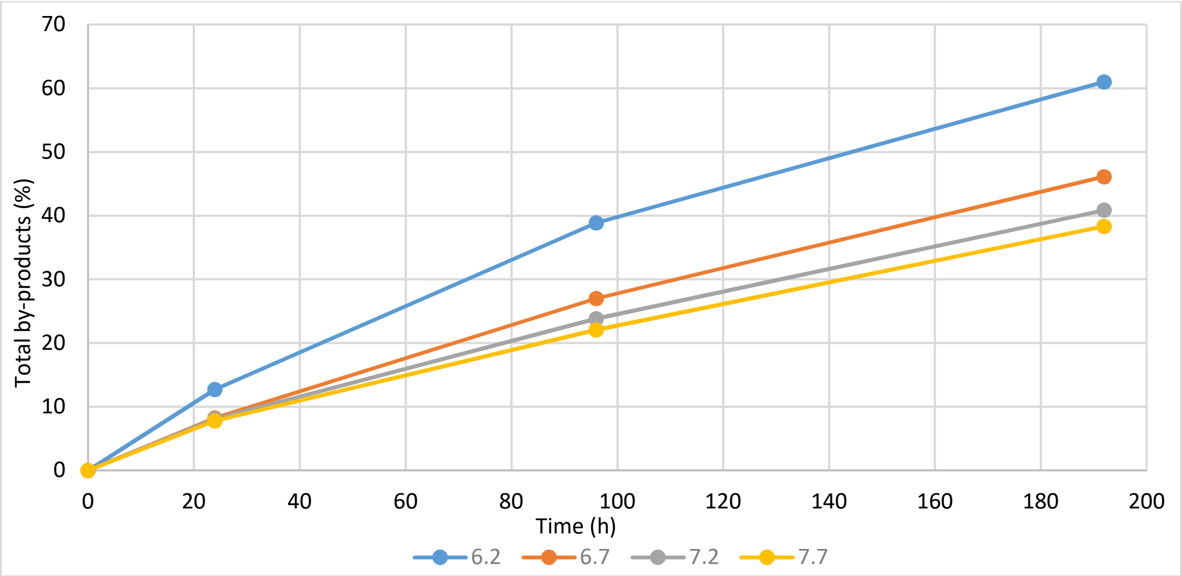

Supplementary figure S23: Influence of the pH on Asi and related byproducts formation (sum of **6**-**10**).

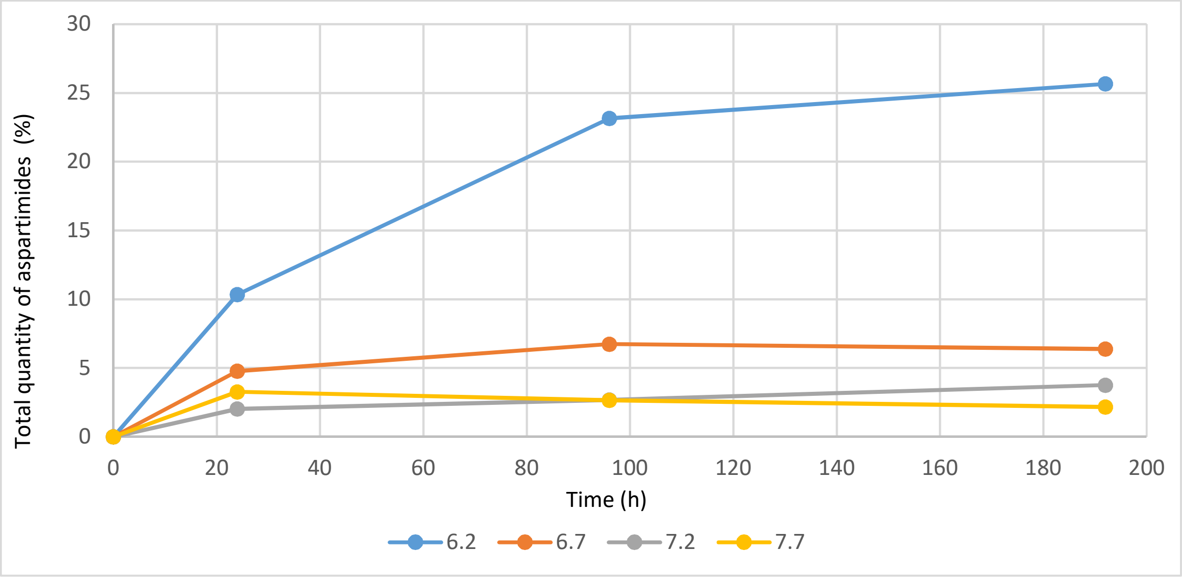

<u>Supplementary figure S24</u>: Influence of the pH on total Asi formation (sum of **6** and **9**).

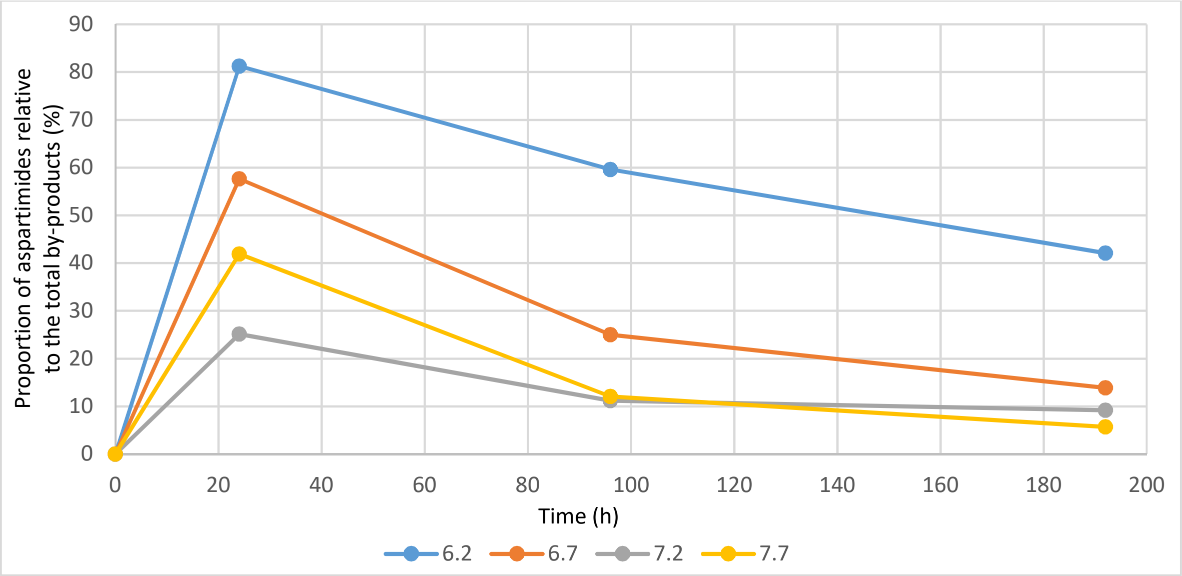

<u>Supplementary figure S25</u>: Influence of the pH on Asi stability (ratio between the sum of **6** and **9** on all byproducts).

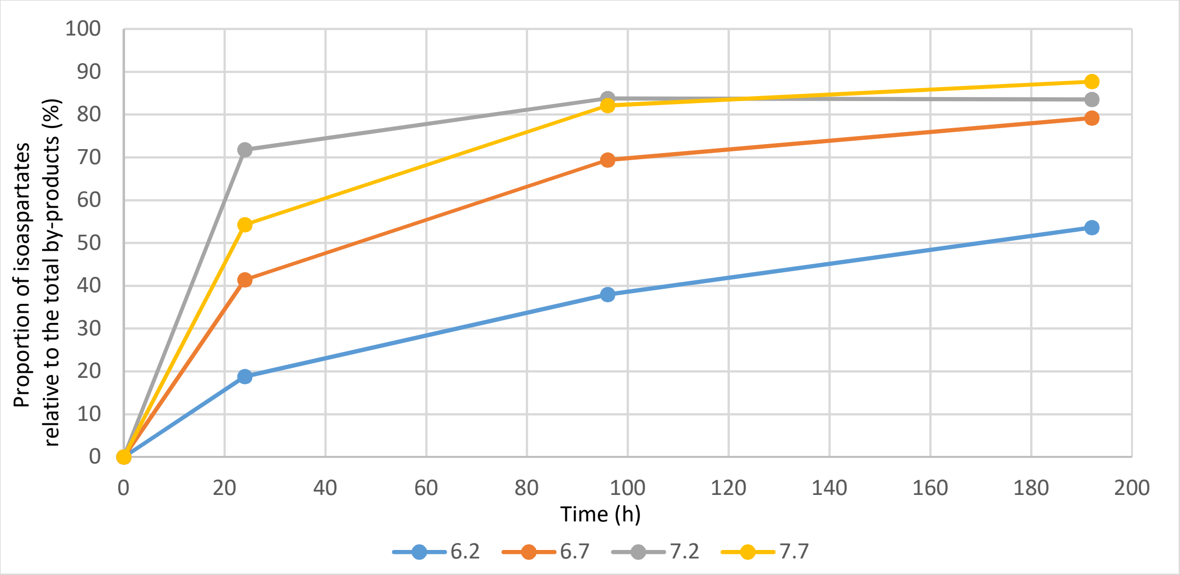

<u>Supplementary figure S26</u>: Influence of the pH on *iso*-residues formation (ratio between **7** and **10** on all byproducts).

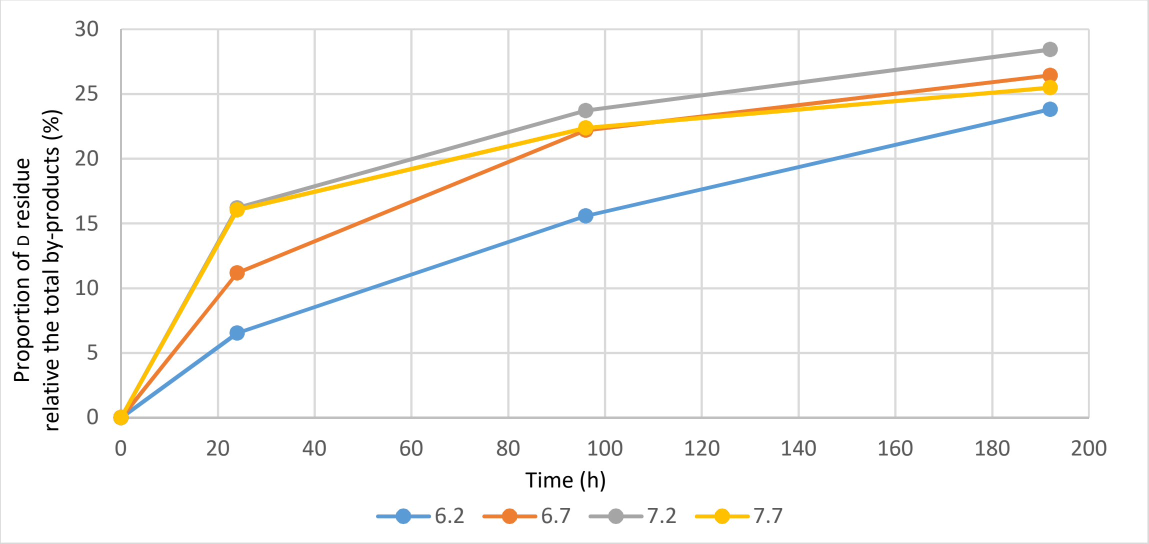

<u>Supplementary figure S27</u>: Influence of the pH on D-residues byproduct formation (ratio between **8, 9** and **10** on all byproducts).

8.5 Influence of the temperature on the rate of formation of Asi and related byproducts.

In order to observe temperature influence of the NCL in Asi and related byproducts. We screened different temperature (50, 37 and 20 °C), using a NCL buffer prepared following the general procedure S9.

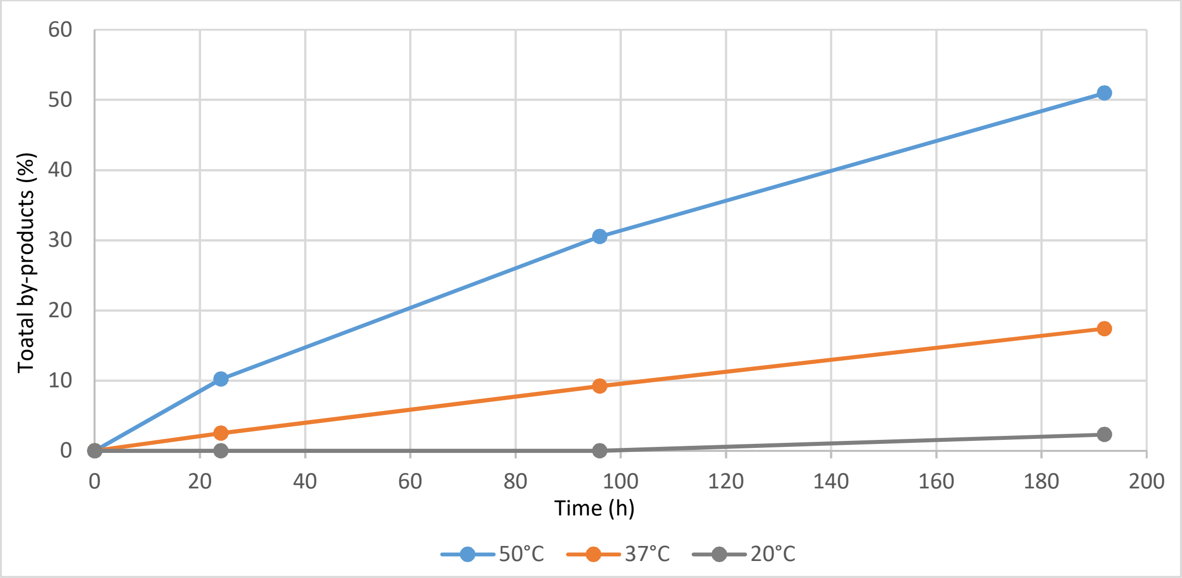

Supplementary figure S28: Influence of the temperature on total byproducts formation (sum of **6**-**10**).

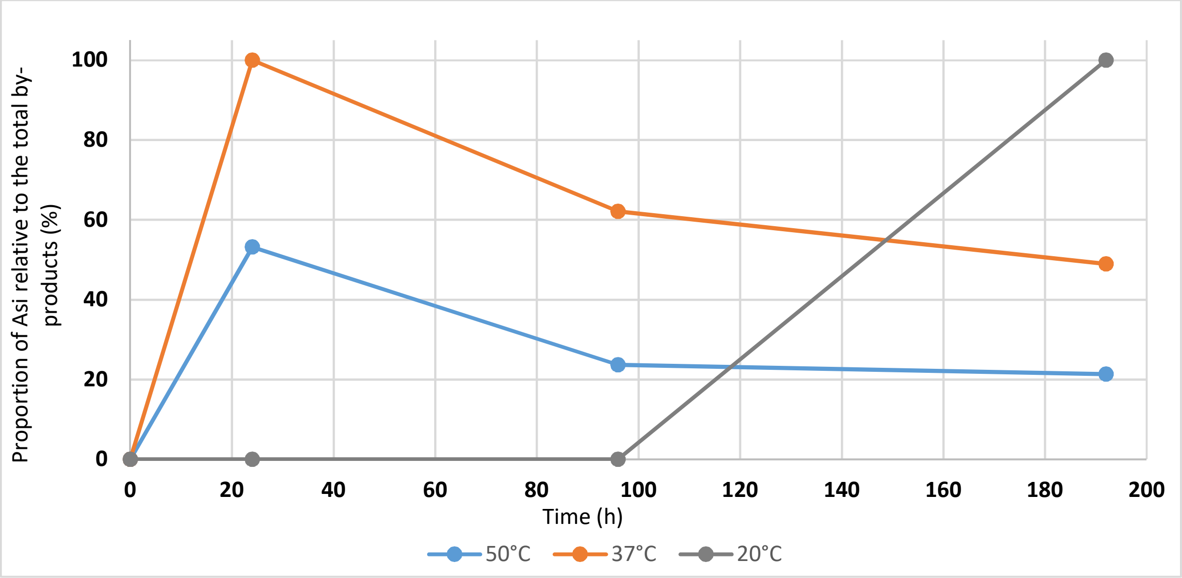

Supplementary figure S29: Influence of the temperature on Asi stability (ratio between the sum of **6** and **9** on all byproducts).

.

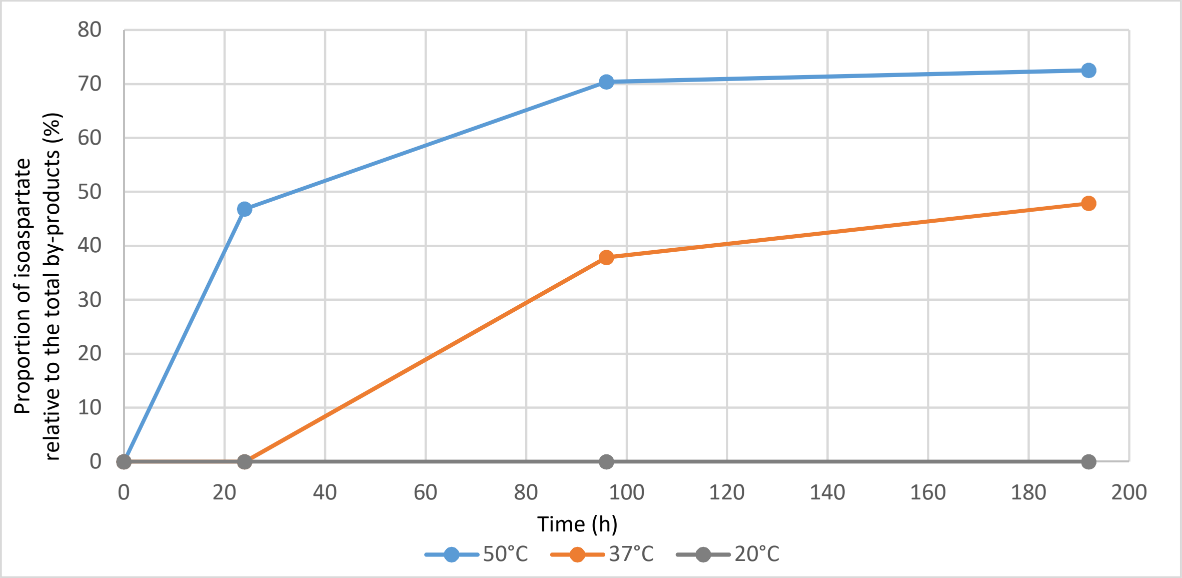

Supplementary figure S30: Influence of the temperature on *iso*-residues formation (ratio between the sum of **7** and **10** on all byproducts).

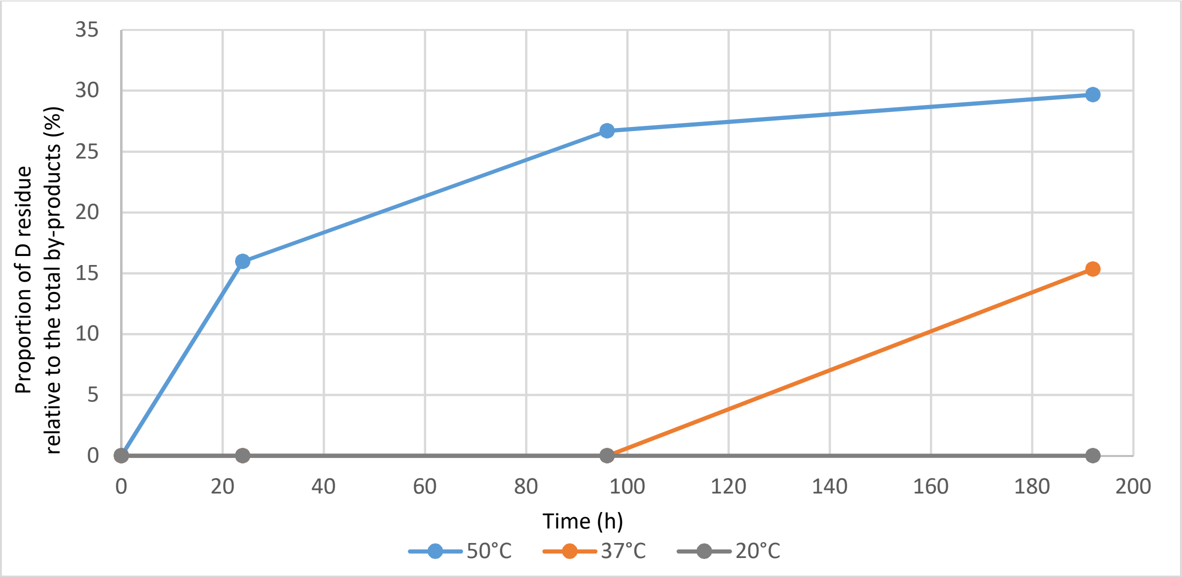

Supplementary figure S31: Influence of the temperature on D-residues product formation (ratio between the sum of **8, 9** and **10** on all byproducts).

9 Estimation of the cumulated amount of aspartimide and related byproducts formed through the assembly of a protein by multiple successive NCLs

This estimation of the total amount of Asi and Asi-derived byproducts that would be formed during the assembly of a protein through consecutive ligations of multiple segments has been calculated by extrapolating that, during each ligation, an equal amount of byproducts as observed in a single ligation would be generated on each Asi-prone hotspots. This probably would somewhat differ from a real situation when tendency for Asi/related byproducts formation would be expected to vary upon each hotspot and probably upon the length and sequence of the intermediate where a given hotspot is located in. In the case of multiple Asi-hotspots-containing segments, we did not attempt to evaluate the generation of multiple byproducts on distinct sites. "% total byproduct-containing product" thus refers to the global number of singly- or multiply-modified NCL products. For ease of calculation in line with the rough estimation didactic purpose of this study, we also neglected the amount of byproducts converted back into a native L-Asp (Asi->Asp, isoAsp->Asi->Asp, D-Asi->Asi->Asp etc.), which contribution is expected to be far below the above-mentioned expected variations.

a. Typical case 1 – Only one single Asi hotspot-containing peptide segment

Supplementary table S04: Estimation of Asi and related byproducts formation through successive NCL, when only one Asi hotspot is present in the whole protein sequence, and is located in one of the two segments of the first ligation of the protein assembly scheme. 2% byproduct formation at the hotspot during each ligation.

NCL #	NCL Product sequence	NCL segment1				NCL segment2				% byproduct-containing product formed from byproduct-free segments during the NCL	% total byproduct-containing product after the NCL
		Sequence[a]	Nb of Asi hotspots	% byproduct-free segment	% byproducts formed on segment1 from byproduct-free segment1 during the NCL	Sequence[a]	Nb of Asi hotspots	% byproduct-free segment	% byproduct formed on segment2 from byproduct-free segment1 during the NCL		
1	AB	A	1	100	2	B	0	100	0	2.00	2.0
2	ABC	AB	1	98.00	1.96	C	0	100	0	1.96	4.0
3	ABCD	ABC	1	96.04	1.92	D	0	100	0	1.92	5.9
4	ABCDE	ABCD	1	94.12	1.88	E	0	100	0	1.88	7.8
5	ABCDEF	ABCDE	1	92.24	1.84	F	0	100	0	1.84	9.6
6	ABCDEFG	ABCDEF	1	90.39	1.81	G	0	100	0	1.81	11.4
7	ABCDEFGH	ABCDEFG	1	88.58	1.77	H	0	100	0	1.77	13.2

a: A is the sole Asi-formation hotspot-containing segment

Supplementary table S05: Estimation of Asi and related byproducts formation through successive NCL, when only one Asi hotspot is present in the whole protein sequence, and is located in one of the two segments of the first ligation of the protein assembly scheme. 5% byproduct formation at the hotspot during each ligation.

NCL #	NCL Product sequence	NCL segment1				NCL segment2				% byproduct-containing product formed from byproduct-free segments during the NCL	% total byproduct-containing product after the NCL
		Sequence[a]	Nb of Asi hotspots	% byproduct-free segment	% byproducts formed on segment1 from byproduct-free segment1 during the NCL	Sequence[a]	Nb of Asi hotspots	% byproduct-free segment	% byproduct formed on segment2 from byproduct-free segment1 during the NCL		
1	AB	A	1	100	5	B	0	100	0	5.00	5.0
2	ABC	AB	1	95.00	4.75	C	0	100	0	4.75	9.8
3	ABCD	ABC	1	90.25	4.51	D	0	100	0	4.51	14.3
4	ABCDE	ABCD	1	85.74	4.29	E	0	100	0	4.29	18.5
5	ABCDEF	ABCDE	1	81.45	4.07	F	0	100	0	4.07	22.6
6	ABCDEFG	ABCDEF	1	77.38	3.87	G	0	100	0	3.87	26.5
7	ABCDEFGH	ABCDEFG	1	73.51	3.68	H	0	100	0	3.68	30.2

a: A is the sole Asi-formation hotspot-containing segment

Supplementary table S06: Estimation of Asi and related byproducts formation through successive NCL, when only one Asi hotspot is present in the whole protein sequence, and is located in one of the two segments of the first ligation of the protein assembly scheme. 10% byproduct formation at the hotspot during each ligation.

NCL #	NCL Product sequence	NCL segment1				NCL segment2				% byproduct-containing product formed from byproduct-free segments during the NCL	% total byproduct-containing product after the NCL
		Sequence[a]	Nb of Asi hotspots	% byproduct-free segment	% byproducts formed on segment1 from byproduct-free segment1 during the NCL	Sequence[a]	Nb of Asi hotspots	% byproduct-free segment	% byproduct formed on segment2 from byproduct-free segment1 during the NCL		
1	AB	A	1	100	10	B	0	100	0	10.00	10.0
2	ABC	AB	1	90.00	9.00	C	0	100	0	9.00	19.0
3	ABCD	ABC	1	81.00	8.10	D	0	100	0	8.10	27.1
4	ABCDE	ABCD	1	72.90	7.29	E	0	100	0	7.29	34.4
5	ABCDEF	ABCDE	1	65.61	6.56	F	0	100	0	6.56	41.0
6	ABCDEFG	ABCDEF	1	59.05	5.90	G	0	100	0	5.90	46.9
7	ABCDEFGH	ABCDEFG	1	53.14	5.31	H	0	100	0	5.31	52.2

a: A is the sole Asi-formation hotspot-containing segment

b. Typical case 2 - One Asi hotspot in each of the segments

In the context of oligoSUMO-2 synthesis through consecutive NCL reactions, each segment used contains an Asi hotspot DG motif. To estimate the total amount of byproducts formation, we followed the same approach as outlined earlier.

Supplementary table S07: Estimation of Asi and related byproducts formation through successive NCL, when one Asi hotspot is present in each of the segments used to assemble the target protein. Exemplified with the oligoSUMO-2 case, expected to cope with such a model of multiple Asi hostspots with similar propension to byproduct formation. 1% byproduct formation at the hotspot during each ligation.

NCL #	NCL product		segment1				segment2				% byproduct-containing product formed from byproduct-free segments during the NCL	% total byproduct-containing product after the NCL
	Name	Sequence[a]	Sequence[a]	Nb of Asi hotspots	% byproduct-free segment	% byproducts formed on segment2 during the NCL with the byproduct-free portion of segment1	Sequence[a]	Nb of Asi hotspots	% byproduct-free segment	% byproducts formed on segment1 from byproduct-free segment1, during the NCL		
1	SUMO-2	NC	C	1	100	1	N	1	100	1	1.99	1.99
2	intermediate	CNC	NC	2	98.01	1.96	C	1	100	1	2.94	4.93
3	$(SUMO2)_2$	NCNC	CNC	3	95.1	2.85	N	1	100	1	3.82	8.75
4	intermediate	CNCNC	NCNC	4	91.2	3.65	C	1	100	1	4.61	13.37
5	$(SUMO2)_3$	NCNCNC	CNCNC	5	86.6	4.33	N	1	100	1	5.29	18.66
6	intermediate	CNCNCNC	NCNCNC	6	81.3	4.88	C	1	100	1	5.83	24.49
7	$(SUMO2)_4$	NCNCNCNC	CNCNCNC	7	75.5	5.29	N	1	100	1	6.23	30.72

a: N refers to SUMO2[1-47] N-terminal segment, C refers to SUMO2[48-93] C-terminal segment

Supplementary table S08: Estimation of Asi and related byproducts formation through successive NCL, when one Asi hotspot is present in each of the segments used to assemble the target protein. Exemplified with the oligoSUMO-2 case, expected to cope with such a model of multiple Asi hostspots with similar propension to byproduct formation. 2% byproduct formation at the hotspot during each ligation.

NCL #	NCL product		segment1				segment2				% byproduct-containing product formed from byproduct-free segments during the NCL	% total byproduct-containing product after the NCL
	Name	Sequence[a]	Sequence[a]	Nb of Asi hotspots	% byproduct-free segment	% byproducts formed on segment2 during the NCL with the byproduct-free portion of segment1	Sequence[a]	Nb of Asi hotspots	% byproduct-free segment	% byproducts formed on segment1 from byproduct-free segment1, during the NCL		
1	SUMO-2	NC	C	1	100	2	N	1	100	2	3.96	3.96
2	intermediate	CNC	NC	2	96.04	3.84	C	1	100	2	5.76	9.72
3	$(SUMO2)_2$	NCNC	CNC	3	90.3	5.42	N	1	100	2	7.31	17.03
4	intermediate	CNCNC	NCNC	4	83.0	6.64	C	1	100	2	8.50	25.54
5	$(SUMO2)_3$	NCNCNC	CNCNC	5	74.5	7.45	N	1	100	2	9.30	34.83
6	intermediate	CNCNCNC	NCNCNC	6	65.2	7.82	C	1	100	2	9.66	44.50
7	$(SUMO2)_4$	NCNCNCNC	CNCNCNC	7	55.5	7.77	N	1	100	2	9.61	54.11

a: N refers to SUMO2[1-47] N-terminal segment, C refers to SUMO2[48-93] C-terminal segment

Supplementary table S09: Estimation of Asi and related byproducts formation through successive NCL, when one Asi hotspot is present in each of the segments used to assemble the target protein. Exemplified with the oligoSUMO-2 case, expected to cope with such a model of multiple Asi hostspots with similar propension to byproduct formation. 5% byproduct formation at the hotspot during each ligation.

NCL #	NCL product		segment1				segment2				% byproduct-containing product formed from byproduct-free segments during the NCL	% total byproduct-containing product after the NCL
	Name	Sequence[a]	Sequence[a]	Nb of Asi hotspots	% byproduct-free segment	% byproducts formed on segment2 during the NCL with the byproduct-free portion of segment1	Sequence[a]	Nb of Asi hotspots	% byproduct-free segment	% byproducts formed on segment1 from byproduct-free segment1, during the NCL		
1	SUMO-2	NC	C	1	100	5	N	1	100	5	9.75	9.75
2	intermediate	CNC	NC	2	90.25	9.03	C	1	100	5	13.57	23.32
3	(SUMO2)$_2$	NCNC	CNC	3	76.7	11.50	N	1	100	5	15.93	39.25
4	intermediate	CNCNC	NCNC	4	60.7	12.15	C	1	100	5	16.54	55.79
5	(SUMO2)$_3$	NCNCNC	CNCNC	5	44.2	11.05	N	1	100	5	15.50	71.29
6	intermediate	CNCNCNC	NCNCNC	6	28.7	8.61	C	1	100	5	13.18	84.47
7	(SUMO2)$_4$	NCNCNCNC	CNCNCNC	7	15.5	5.43	N	1	100	5	10.16	94.64

a: N refers to SUMO2[1-47] N-terminal segment, C refers to SUMO2[48-93] C-terminal segment

10 Development of the Hmb strategy

10.1 Acetylation conditions

To quantify Hmb incorporation through reductive amination using RP-HPLC analysis, we carried out an acetylation treatment to preserve Hmb during TFA cleavage. Full acetylation was confirmed on the peptide SUMO-2[61-66] **S02**, where the Fmoc-Asp(O*t*Bu)-(Hmb)Gly-OH building block was utilized to readily obtain an Hmb-equipped segment. We observed complete acetylation by treating the peptidyl-resin with an NMP solution containing 0.1 M Ac_2O and 0.5 M DIEA for 1.5 h, followed by TFA cleavage (compound **S03**). These conditions were inspired by the acetylation method described by Abdel-Aal, A. M. *et al.*[5] and wered defined as the general protocols PS7 Extending the TFA cleavage treatment from 1.5 to 4 h did not affect the acetylated Hmb levels.

10.2 Hmb incorporation through solid-supported reductive amination

a. Optimization on a short peptide sequence

The Hmb incorporation tests, carried out in two distinct steps (imination and reduction), using 2-hydroxy-4-methoxybenzaldehyde (**11**), were performed during the synthesis of model peptide **S02** and monitored by RP-HPLC following the general protocol PS7. Cleavage was performed for 2 h following protocol PS5.

Supplementary table S10: Different other reductive amination conditions tested[a]

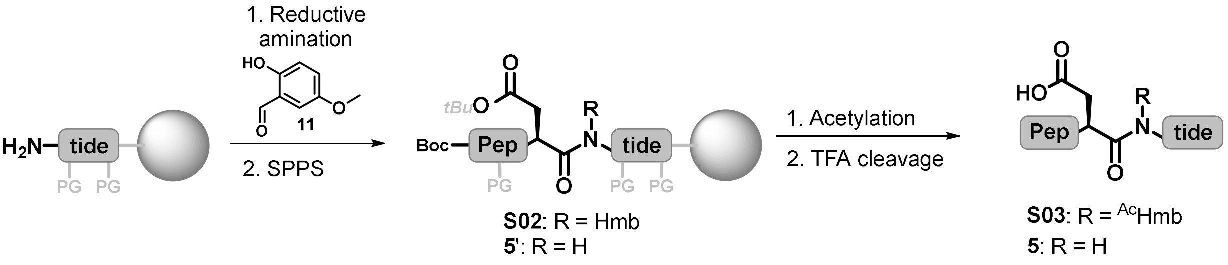

Peptide sequence: SUMO-2[61-67]W (RFDGQPW-NH2)

Entry	Imination solvent[b]	Reducing agent[c]	Reduction solvent	Reduction time (min)	Hmb incorporation (%)[d]
1	DMF/MeOH/AcOH (44.5:44.5:1)	$NaCNBH_3$	DMF/MeOH/AcOH (44.5:44.5:1)	60	15
2	DMF/MeOH/AcOH (44.5:44.5:1)	$NaBH_4$	DMF	20	>99
3	DMF/MeOH/AcOH (44.5:44.5:1)	$NaBH_4$	DMF/MeOH (3:1)	20	>99
4	DMF/AcOH (99:1)	$NaBH_4$	DMF	20	>99

[a]: all reactions were conducted at room temperature at a 12.5 mM peptidyl resin concentration; [b]: imination solvent contained 10 equiv. of aldehyde **11**, reaction was stirred during 1 h, followed by a wash step with DMF/MeOH then DMF; [c]: reducing solvent contained 20 equiv. of the reducing agent; [d]: relatives amounts of **S03** and **5** determined by HPLC peak integration at λ= 280 nm, neglecting the contribution of AcHmb on the molar absorption coefficient of **S03**.

64Gly-*N*-AcHmb-SUMO-2[61-66]W-NH_2 (**S03**)

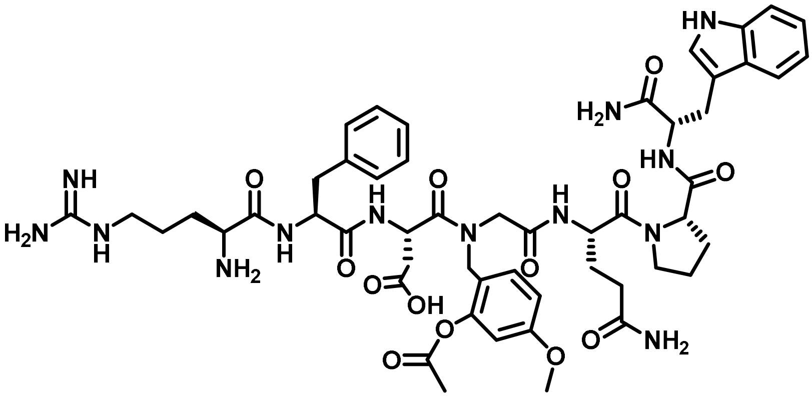

H-Arg-Phe-Asp-(AcHmb)Gly-Gln-Pro-Trp-NH_2 (**19**)

ESI-MS (*m/z*): [M+H] calcd. for $C_{52}H_{67}N_{13}O_{13}^+$: 1082.5, found: 1082.4 (monoisotopic mass).

HPLC analysis: t_R = 4.22 min (Chromolith, gradient: 5-50% B/A over 5 min).

b. Optimization on a long peptide sequence

To improve the introduction of Hmb into a long peptide sequence we used SUMO-2[47-93]-OH segment and we quantified by RP-HPLC the relative amounts of 64Gly-*N*-AcHmb Cys(K_6-Ades)-SUMO-2[48-93]-OH segment (**S04**) and the one not incorporating an Hmb (Cys(K_6-Ades)-SUMO-2[48-93]-OH, **S05**). Fmoc SPPS was performed using the conditions described for 64Gly-*N*-Hmb Cys(GABA-K_6-Ades)-SUMO-2[48-93]-OH cysteinyl peptide (**14**), detailed synthesis p S47, with the exception of the last Lys. This residu was incorporated using Boc-Lys(Boc)-OH.

Supplementary table S11: Different others reductive amination conditions tested[a]

Entry	Imination time (min)	Reduction time (min)	Hmb incorporation (%)[b]
1	5	20	20
2	60	20	76
3	2 x 120[c]	60	>99
4	2 x 120[c]	20	>99

[a]: all reactions were conducted at room temperature at a 12.5 mM peptidyl resin concentration using 10 equiv. of aldehyde **11** solubilized in DMF/MeOH/AcOH (44.5:44.5:1) imination solvent, and using 20 equiv. of $NaBH_4$ in DMF for the reduction step; [b]: relatives amounts of **S04** and **S05** determined by HPLC peak integration at λ= 280 nm, neglecting the contribution of ^{Ac}Hmb on the molar absorption coefficient of **S04**; [c]: two successive 2 h imination.

The conditions developed in entry 4 were used for the Protocol PS3 –Optimized reductive amination: introduction of the Hmb group.

^{64}Gly-*N*-^{Ac}Hmb Cys(K_6-Ades)-SUMO-2[49-93]-OH (**S04**)

ESI-MS (*m/z*): [M] calcd. for $C_{275}H_{445}N_{79}O_{89}S_2$: 6346.1, found: 6345.1 (average mass, deconvoluted).

HPLC analysis: t_R = 7.94 min (Aeris Widepore XB-C18 2, gradient: 3-50% B/A over 11 min at 60°C).

Cys(K_6-Ades)-SUMO-2[49-93]-OH (**S05**)

ESI-MS (*m/z*): [M] calcd. for $C_{265}H_{435}N_{79}O_{86}S_2$: 6167.9, found: 6166.9 (average mass, deconvoluted).

HPLC analysis: t_R = 7.37 min (Aeris Widepore XB-C18 2, gradient: 3-50% B/A over 11 min at 60°C).

10.3 Solid phase *O*-GABAylation of Hmb

10.3.1 Synthesis of *N*-Boc-γ-aminobutanoic anhydride (12)

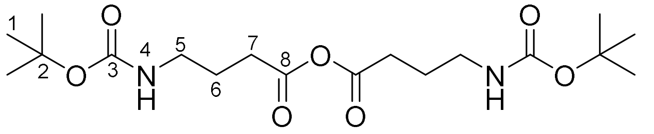

[CAS N°: 89231-63-0]

N-Boc-γ-aminobutyric acid (10 g, 49.2 mmol, 1 equiv.) was dissolved in CH_2Cl_2 (80 mL) in a round bottom flask and then a solution of DCC (5 g, 24.6 mmol, 0.5 equiv.) in CH_2Cl_2 (20 mL) was added. A white precipitate (DCU) was formed and stirring was continued for 1 h at RT. After 2 h cooling at – 20 °C, the mixture was filtered to remove DCU, which was washed with cold (-20 °C) CH_2Cl_2. The filtrate and the washings were concentrated under reduced pressure, to afford *N*-Boc-γ-aminobutyric anhydride (**12**) as a white solid, which was used without further purification. (100 % isolated yield)

ESI-HRMS (*m/z*): $[M+Na]^+$ calcd. for $C_{18}H_{32}N_2NaO_7$: 411.2107 found: 411.2100.

^{1}H NMR (600 MHz, $CDCl_3$): δ 4.69 (bt, *J* = 6.4 Hz, 2H, NH), 3.14-3.22 (m, 4H, H_5), 2.51 (t, *J* = 7.2 Hz, 4H, 2 H_7), 1.85 (tt, *J* = 7.0 Hz, 7.0 Hz, 4H, H_6), 1.43 (s, 18H, H_{tBu}).

^{13}C NMR (151 MHz, $CDCl_3$): δ 169.1 (C_{co}), 156.2 (C_{co}), 79.5 (C_{tBu}); 39.6 (C_5), 32.5 (C_7), 28.5 ($CH_{3\ tBu}$), 24.8 (C_6).

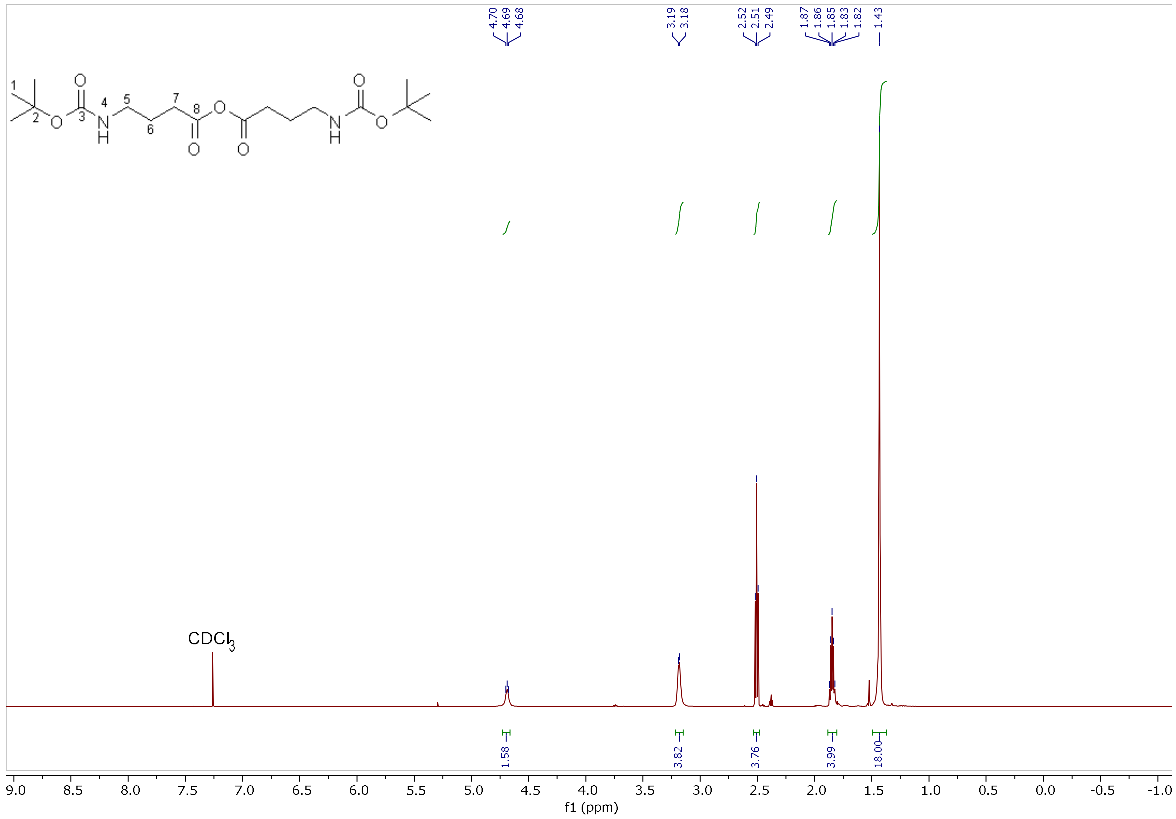

Supplementary figure S34: Copy of the ^{1}H NMR spectrum of **14** (600 MHz, $CDCl_3$).

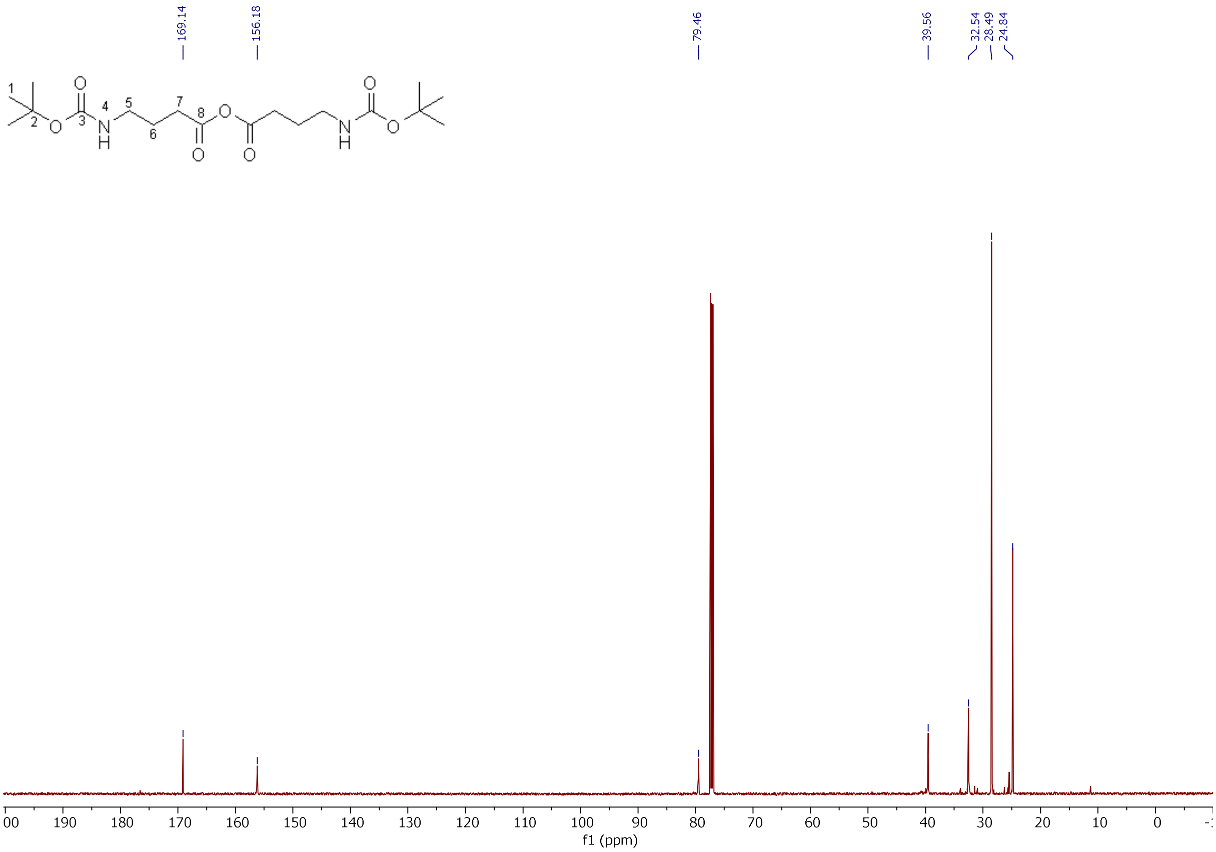

Supplementary figure S35: Copy of the ^{13}C NMR spectrum of **14** (151 MHz, $CDCl_3$).

Supplementary table S12: Different GABAylation conditions tested[a]

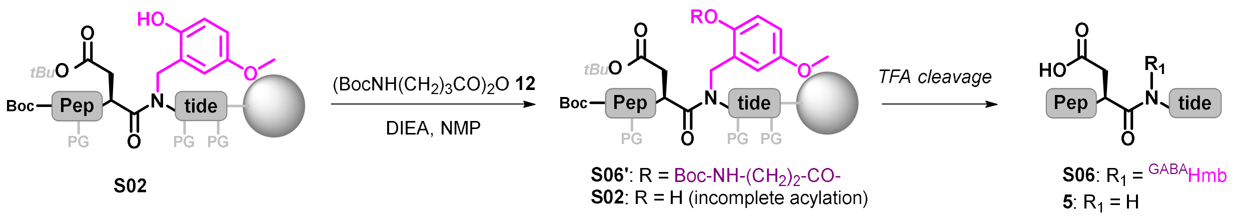

Peptide sequence: SUMO-2[61-66]W (RFDGQPW-NH2)

Entry	Conditions	Hmb GABAylation (%)[b]			
		15 min	30 min	2 h	18 h
1	No additives	29	42	73	> 99
2	DBU instead of DIEA	> 99	> 99	> 99	> 99
3	20% (*v*/*v*) pyridine	33	47	76	> 99
4	1 mol% DMAP	> 99	> 99	> 99	> 99

[a]: all reactions were conducted at room temperature at a 0.5 M *N*-Boc-γ-aminobutyric anhydride **12** concentration and 0.5 M of base in NMP, then the reaction mixture was washed with NMP, before the TFA-mediated cleavage step; [b]: relatives amounts of **S06** and **5** determined by HPLC peak integration at λ= 280 nm, neglecting the contribution of GABAHmb on the molar absorption coefficient of **S06**.

The conditions developed in **entry 4** were used for protocol PS6 –Solid phase Hmb *O*-GABAylation.

64Gly-*N*-GABAHmb-SUMO-2[61-66]W-NH_2 (**S06**)

ESI-MS (*m/z*): [M+H] calcd. for $C_{54}H_{73}N_{14}O_{13}^{+}$: 1126.4, found: 1126.4(monoisotopic mass).

HPLC analysis: t_R = 2.53 min (Chromolith, gradient: 18-28% B/A over 5 min).

10.4 Monitoring of the self-immolation of the GABA group on the Hmb

The spontaneous removal of the GABA group of peptide **S06** was monitored by RP-HPLC. The peptide was solubilized at 1 mg/mL in a PBS (pH = 7.4)/MeCN 8:2 mixture. A complete loss of the GABA was observed within 30 min, and a half-life of 1.3 min was estimated. Moreover, we compared the GABA cyclization (GABAHmb compound **S06**) to acetate hydrolysis (AcHmb compound **S03**) under the same conditions.

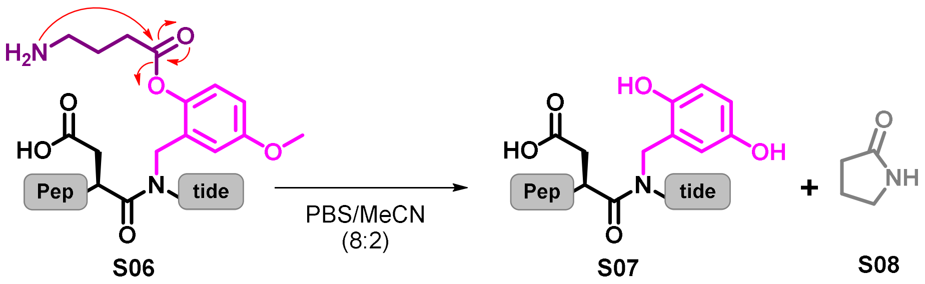

Peptide sequence: SUMO-2[61-66]W (RFDGQPW-NH2)

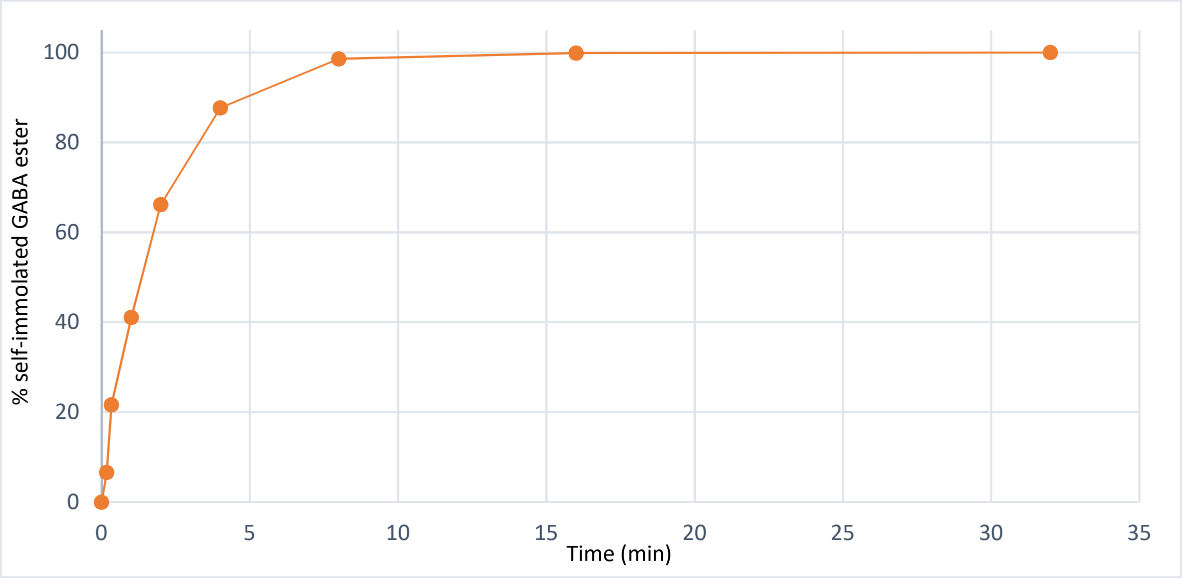

[a]: incubation was performed incubating peptide at 1 mM in a PBS/MeCN 8:2 mixture. GABA self-immolation rate corresponds to the relatives amounts of **S07** and **S06** determined by HPLC peak integration at λ= 280 nm, neglecting the contribution of Hmb and ^{GABA}Hmb on the molar absorption coefficients of **S06** and **S07**, respectively.

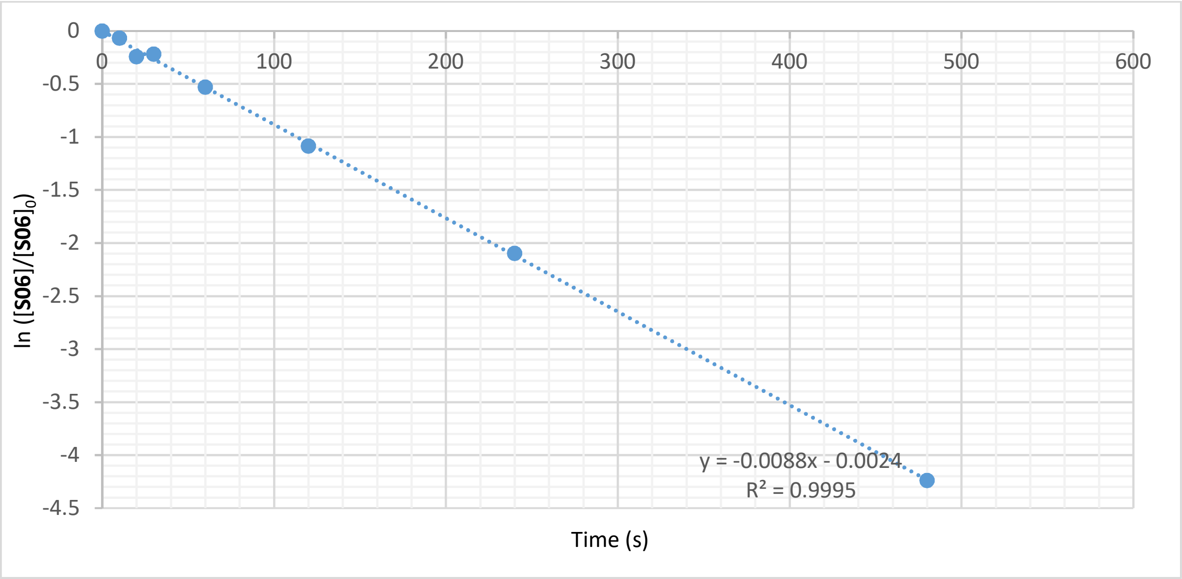

[b]: determination of the first order kinetic constant of the ^{GABA}Hmb self-immolation (k = 0.0088 s^{-1})

<u>Supplementary figure S36</u>: Monitoring of ^{GABA}Hmb self-immolation and first-order kinetic constant determination

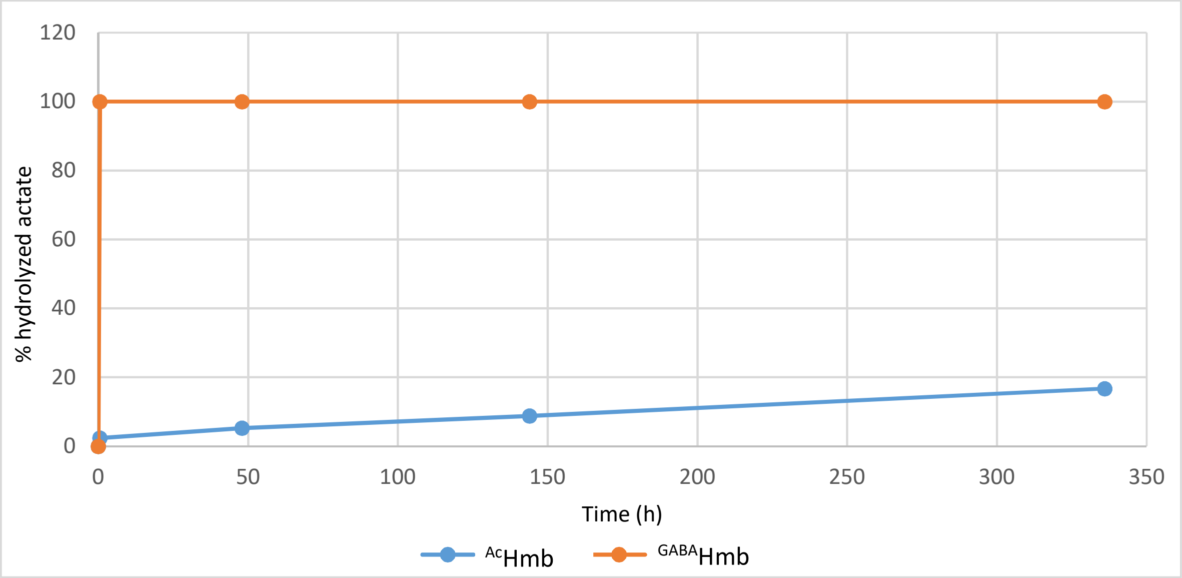

[a]: incubation was performed incubating peptide at 1 mM in a PBS/MeCN 8:2 mixture. Acetate hydrolysis rate corresponds to the relatives amounts of **S07** and **S03** determined by HPLC peak integration at λ= 280 nm, neglecting the contribution of Hmb and ^{Ac}Hmb on the molar absorption coefficients of **S06** and **S03**, respectively.

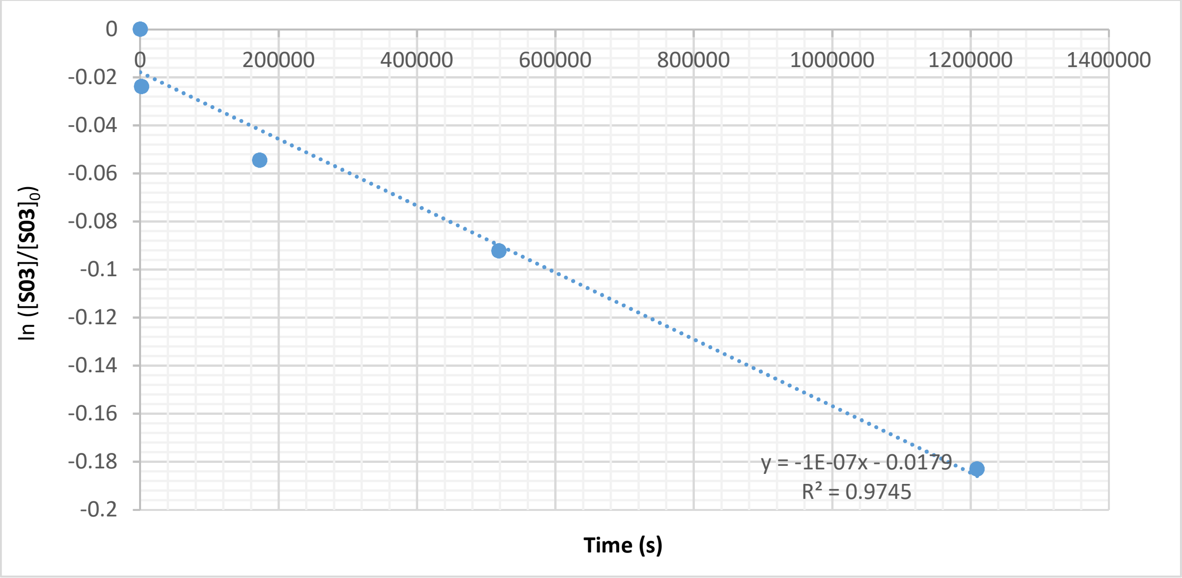

[b]: determination of the first order kinetic constant of the ^{Ac}Hmb hydrolysis (k = 0.0000001 s^{-1}).

Supplementary figure S37: Monitoring of ^{Ac}Hmb self-immolation and comparison with ^{GABA}Hmb self-immolation, and first-order kinetic constant determination.

10.5 Synthesis of ^{64}Gly-*N*-^{Ac}Hmb-SUMO-2[61-66]W-NH$_2$ (S07)

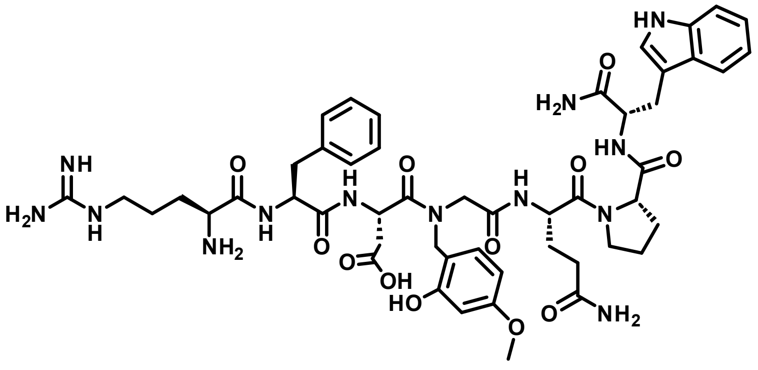

The peptide was synthesized on Rink amide MBHA polystyrene resin (0.68 mmol/g) using protocol PS1. Hmb was incorporated according to protocol PS3 and the last Arg residue with Boc-Arg(Pbf)-OH building-block. Prior to resin cleavage, Hmb was GABAylated using protocol PS6. Cleavage was performed for 2 h following protocol PS5. The crude cleaved peptide was solubilized in PBS/MeCN according to protocol PS8 to remove the GABA group prior to semi-preparative HPLC purification.

ESI-MS (*m/z*): $[M+H]^+$ calcd. for $C_{50}H_{66}N_{13}O_{12}$: 1040.5, found: 1040.5 (monoisotopic mass).

HPLC analysis: t_R = 3.95 min (Chromolith, gradient: 5-50% B/A over 5 min).

HPLC purification: [5 mg/mL] Nucleosil C18, gradient: 15-45% B/A over 60 min. (47%)

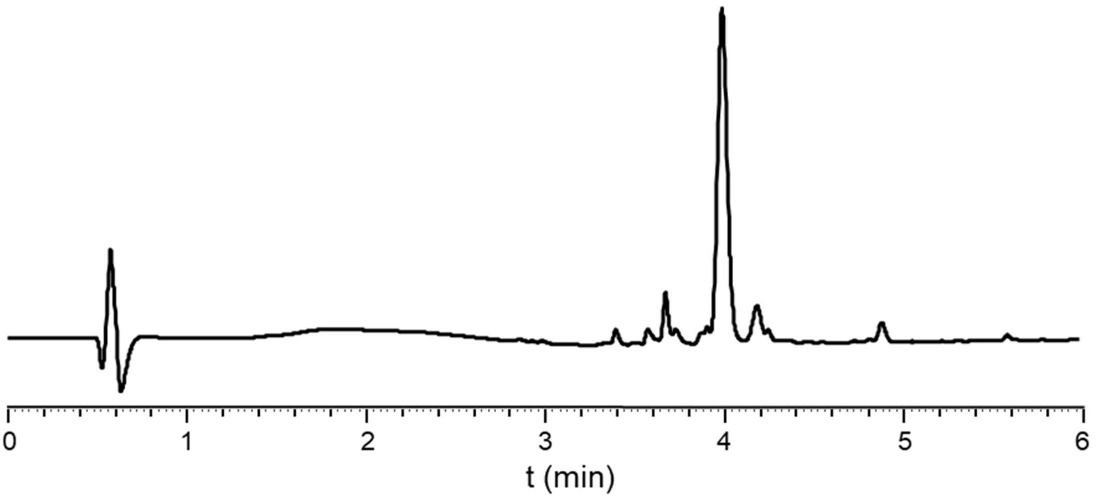

Supplementary figure S37: HPLC trace of crude **S07** (λ = 214 nm).

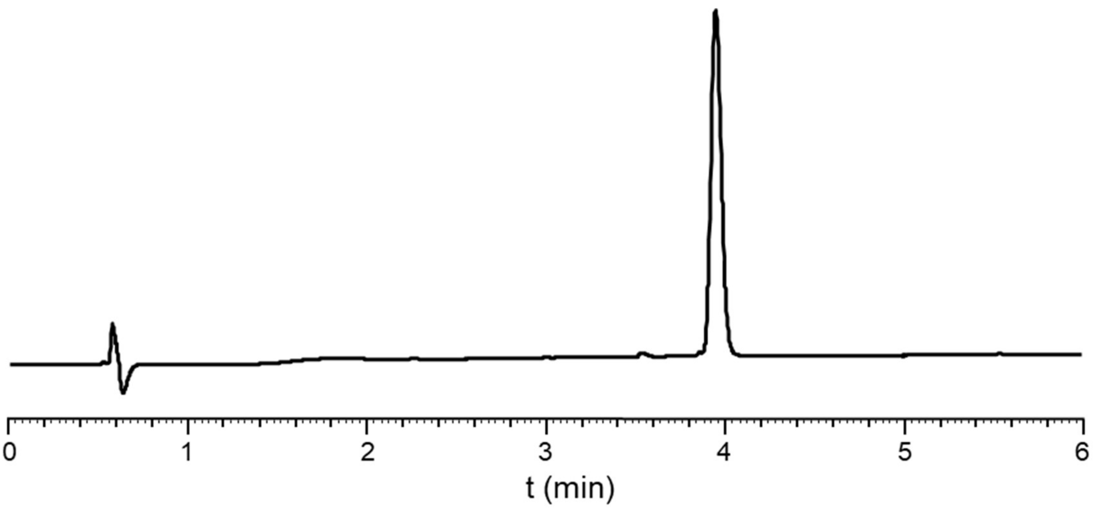

Supplementary figure S38: HPLC trace of purified **S07** (λ = 214 nm).

10.6 Synthesis of ^{64}Gly-*N*-^{Ac}Hmb-SUMO-2[61-66]W-NH$_2$ D63N (S09) under optimized conditions

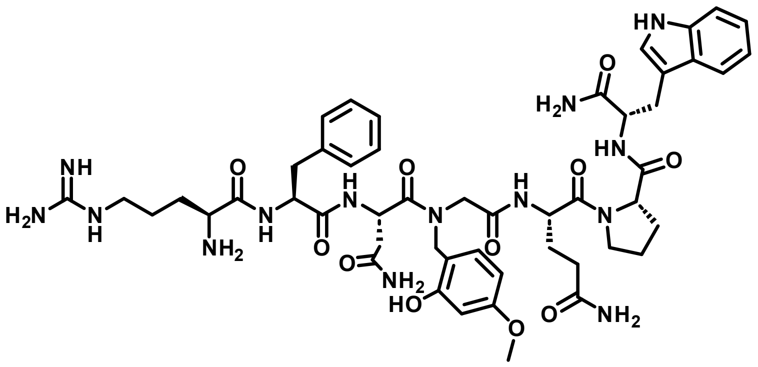

The peptide was synthesized on Rink amide MBHA polystyrene resin (0.68 mmol/g) using protocol PS1. Hmb was incorporated according to protocol PS3 and the last Arg residue with Boc-Arg(Pbf)-OH building-block. Prior to resin cleavage, Hmb was GABAylated using protocol PS6. Cleavage was performed for 2 h following protocol PS5. The crude cleaved peptide was solubilized in PBS/MeCN according to protocol PS8 to remove the GABA group. No further purification has been performed.

ESI-MS (*m/z*): $[M+H]^+$ calcd. for $C_{50}H_{66}N_{13}O_{12}$: 1039.5, found: 1039.5 (monoisotopic mass).

HPLC analysis: t_R = 4.16 min (Chromolith, gradient: 5-50% B/A over 5 min).

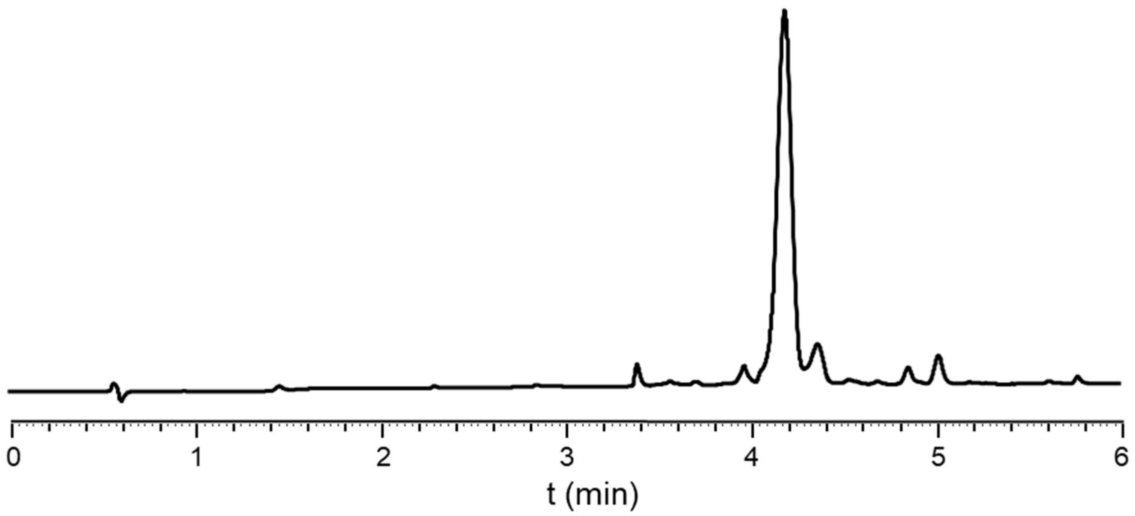

Supplementary figure S39: HPLC trace of crude **S09** (λ = 214 nm).

10.7 Estimation of the molar extinction coefficient of the Hmb group at λ = 280 nm

For the purpose of UV spectrophotometry-based quantification of SUMO-2 segments, we measured the molar absorption coefficient of Hmb at λ = 280 nm by synthesizing a Hmb-containing model compound (**S10**).

- **Synthesis of the *N*-GABA-aspartidyl-*N*-Hmb-glycinamide (S10)**

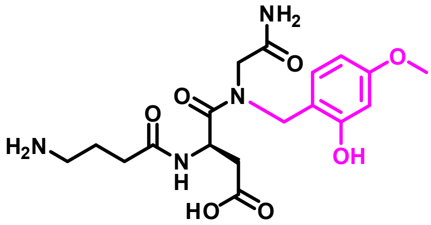

The dipeptide was synthesized on Rink amide MBHA polystyrene resin (0.68 mmol/g) following protocol PS1 and using Fmoc-Asp(O*t*Bu)-(Hmb)Gly-OH. Hmb was GABAylated using protocol PS6. Cleavage was performed for 2 h following protocol PS5. The crude cleaved peptide was solubilized in PBS/MeCN according to protocol PS8 to remove the GABA group prior to semi-preparative HPLC purification.

ESI-MS (*m/z*): [M+H] calcd. for $C_{18}H_{27}N_4O_7$: 411.4, found: 411.0 (monoisotopic mass).

HPLC analysis: t_R = 3.16 min (Chromolith, gradient: 02-30% B/A over 5 min).

HPLC purification: [5 mg/mL] Nucleosil C18, gradient: 5-7 % B/A over 15 min affording a white solid after lyophilisation (74%).

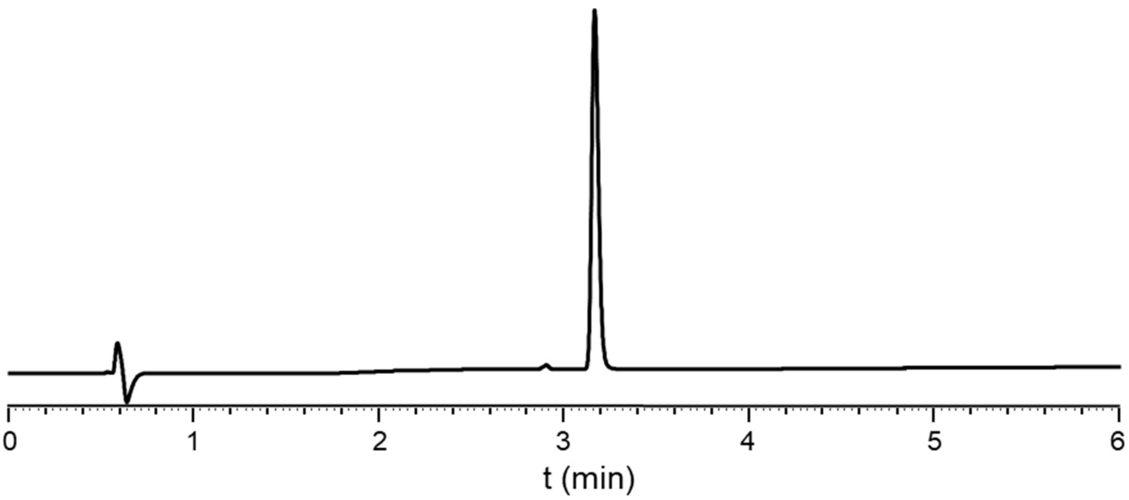

Supplementary figure S40: HPLC trace of purified **S10** (λ = 214 nm).

- **Determination of the molar extinction coefficient (ε):**

S10 was solubilized in water at a 1.57 mM concentration (determined by weight, considering one trifluoroacetate counter-ion) and an UV absorption spectrum (215-400 nm) was acquired, allowing to determine the molar extinction coefficient of the Hmb group at 280 nm using the Beer-Lambert law.

ε_{280nm} = 1607 L.mol-1.cm−1.

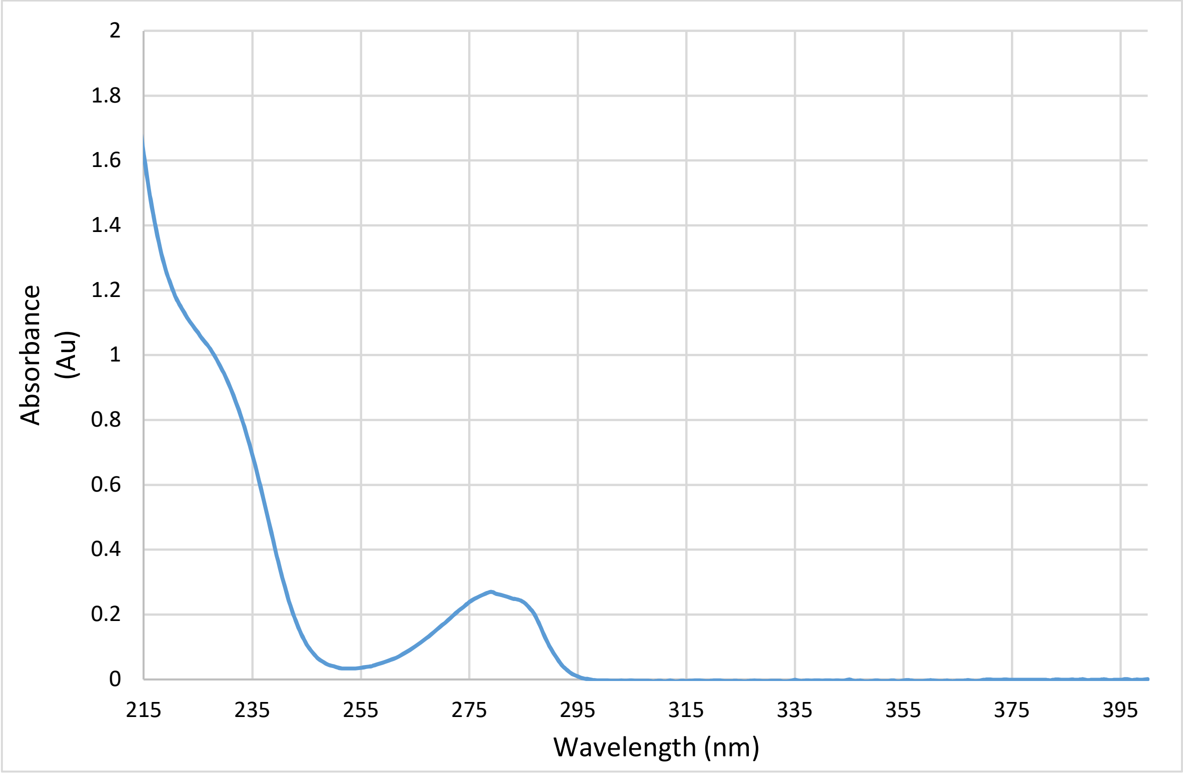

Supplementary figure S41: Absorption spectrum of **S10.**

11 Synthesis and purification of SUMO-2 derived peptide segments

11.1 ^{27}Gly-*N*-Hmb SUMO-2[1-47]-(Hnb)Cys(S*t*Bu)-Gly-NH$_2$ crypto-thioester (13a)

Sequence:

H-XADEKPKEGVKTENNDHINLKVAGQD(Hmb)GSVVQFKIKRHTPLSKLXKAY-(Hnb)C(S*t*Bu)-G-NH$_2$

(X= Norleucine)

The peptide was synthesized on Rink amide ChemMatrix® resin (0.37 mmol/g) using protocol PS1 and the last Nle residue was incorporated using Boc-Nle-OH. Hnb was introduced using protocol PS2. Hmb was incorporated according to protocol PS3 to prevent Asi formation during SPPS and NCL. To allow UV titration of the amount of cleaved Fmoc and determine an SPPS elongation yield, peptidyl resin was treated according to protocol PS4 before the final Fmoc deprotection of Fmoc-Ala2. Prior to resin cleavage, Hmb was GABAylated using protocol PS6. Cleavage was performed for 4 h following protocol PS5. The crude cleaved peptide was solubilized in PBS/MeCN according to protocol PS8 to remove the GABA group prior to semi-preparative HPLC purification.

Elongation yield: 38%. Determined by the ratio between the quantity of fluorenylpiperidine released during final Fmoc deprotection (Protocol PS6) and the quantity released during the Fmoc deprotection of the C-terminal Gly residue (UV titration at 301 nm, ε = 7800 L.mol^{-1}.cm^{-1}).

ESI-MS (*m/z*): [M] calcd. for $C_{258}H_{417}N_{71}O_{76}S_2$: 5793.6, found : 5792.6 (average mass, deconvoluted).

HPLC analysis: t_R = 3.07 min (Chromolith, gradient: 30-40% B/A over 5 min).

HPLC purification: [5 mg/mL] Nucleosil C18, gradient: 15-40% B/A over 60 min affording a white solid after lyophilisation (27%).

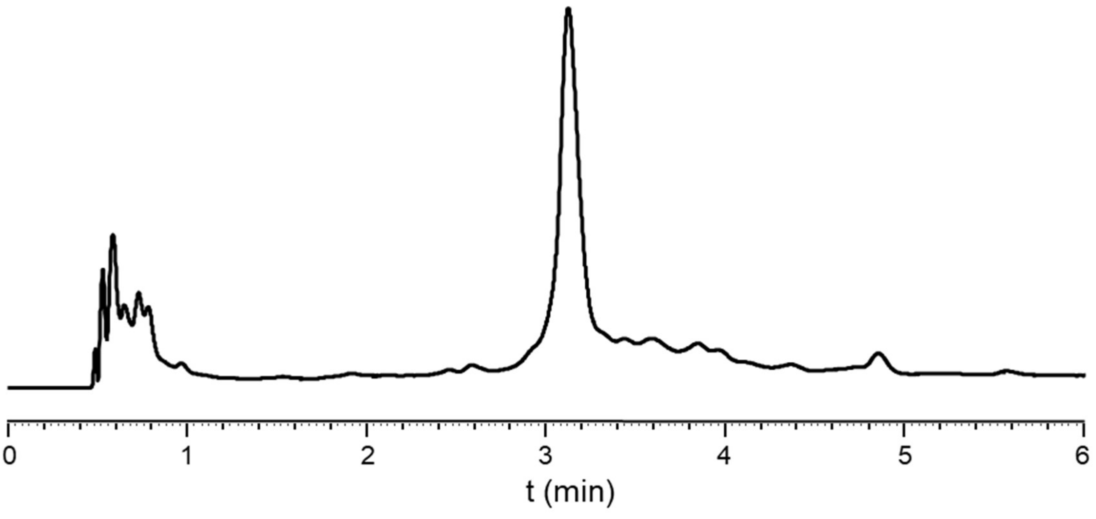

Supplementary figure S42: HPLC trace of crude **13a** (λ = 214 nm).

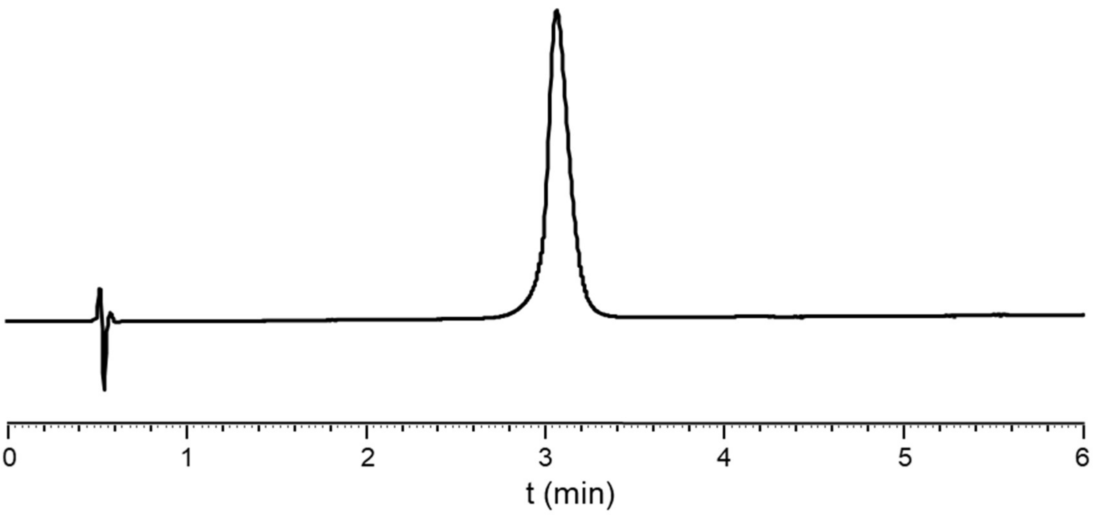

Supplementary figure S43: HPLC trace of purified **13a** (λ = 214 nm).

11.2 Synthesis of the SUMO-2 C-ter segments 14a and 20a

11.2.1 Synthesis of Boc-Cys(Fmoc-Ades)-OH (17)

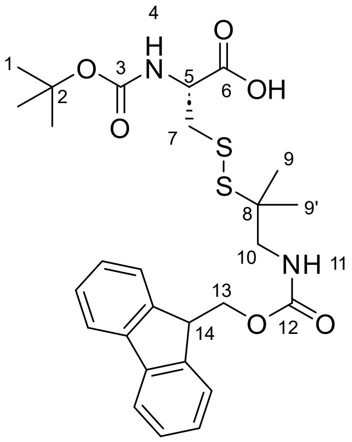

Boc-Cys(Npys)-OH (500 mg, 1.33 mmol, 1 equiv.) was dissolved in DMF (6.65 mL, 0.2 M) in a round bottom flask and then 1-amino-2methylpropane-2-thiol hydrochloride (158 mg, 1.5 mmol, 1.1 equiv.) was added. The reaction mixture was stirred during 3 h and was monitored by TLC and LC-MS. Then, water (0.67 mL), Fmoc-OSu (897.3 mg, 2.66 mmol, 2 equiv.) and DIEA (698 µL, 4 mmol, 3 equiv.) were added. After 4 h of stirring at RT, the reaction was diluted with AcOEt (30 mL). The organic phase was washed 3 times with a 1 M aqueous solution of HCl (20 mL), then 4 times with brine (20 mL). The organic extract was dried over $MgSO_4$, evaporated and the crude was purified by flash chromatography (100% DCM, then from 97:3:0.5 to 70:30:0.5 DCM/AcOEt/TFA). In order to prevent TFA-mediated Boc deprotection during concentration, the pooled pure fractions were diluted with an equal volume of toluene, then slowly evaporated under reduced pressure at 20 °C to give the desired product as a beige foam. (75 % isolated yield)

ESI-HRMS (*m/z*): $[M+H]^+$ calcd. for $C_{27}H_{35}N_2O_6S_2^+$: 547.1931 found: 547.1929.

HPLC analysis: t_R = 3.14 min (Chromolith, gradient: 50-70% B over 9 min).

Pure **17** has been analyzed by ^{1}H NMR in DMSO-*d6*. Two sets of chemical shifts corresponding to a 9:1 mixture of conformers are observed, presumably *trans* and *cis* rotamers of a carbamate. The equilibrium between the two forms was confirmed by a 2D NMR exchange spectrometry experiment (EXSY).

Major conformer**:**

^{1}H NMR (600 MHz, DMSO-d_6): δ 12.92 (bs, 1H, COOH) 7.88 (d, *J* = 7.6 Hz, 2H, H_{Ar}), 7.72 (d, *J* = 7.5 Hz; 2H, H_{Ar}), 7.50 (bt, 1H, H_{11}), 7.41 (t, *J* = 7.5 Hz, 2H, H_{Ar}), 7.32 (t, *J* = 7.5 Hz, 2H, H_{Ar}), 7.10 (bd, 1H, H_4), 4.30 (d, *J* = 7.0 Hz, 2H, H_{13}), 4.22 (t, *J* = 7.1 Hz, 1H, H_5), 4.19 – 4.13 (m, 1H, H_{14}), 3.21 – 3.15 (m, 2H, H_{10}), 3.06 (dd, *J* = 13.2, 4.2 Hz, 1H, H_{7a}), 3.02 – 2.96 (m, 1H, H_{7b}), 1.38 (s, 9H, H_1) , 1.21 (s, 3H, H_9), 1.19 (s, 3H, $H_{9'}$).

^{13}C NMR (151 MHz, DMSO-d_6)**:** δ 172.4 (C_6), 156.6, 155.3 (C_{12} and C_3), 143.9 (C_{Ar}), 140.7 (C_{Ar}), 127.6 (CH_{Ar}), 127.1 (CH_{Ar}), 125.3 (CH_{Ar}), 120.1 (CH_{Ar}), 78.2 (C_2), 65.5 (C_{13}), 53.2 (C_{14}), 51.3 (C_8), 49.4 (C_{10}), 46.8 (C_5), 28.2 (C_1), 25.1(C_9) , 25.0 ($C_{9'}$).

Minor isomer (selected peaks):

^{1}H NMR (600 MHz, DMSO-d_6)**:** δ 7.83 (d, *J* = 7.6 Hz, 2H, H_{Ar}), 7.66 (d, *J* = 7.6 Hz, 2H, H_{Ar}), 6.92 (bs, 1H, NH), 6.76 (bs, 1H, NH), 2.91 (bs, 2H, H_{10}), 0.96 (s, 3H, H_9), 0.94 (s, 3H, $H_{9'}$).

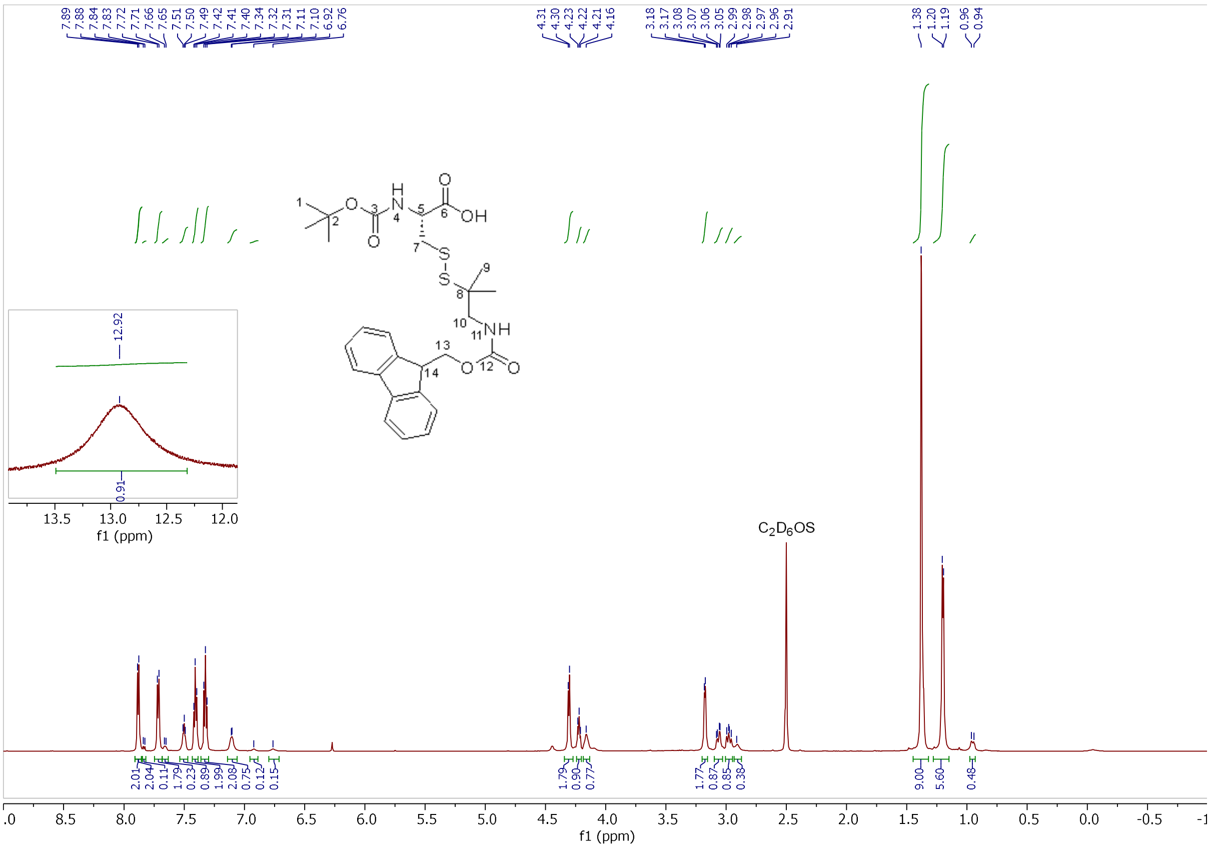

Supplementary figure S44: Copy of the ^{1}H NMR spectrum of **17** (600 MHz, DMSO-d_6).

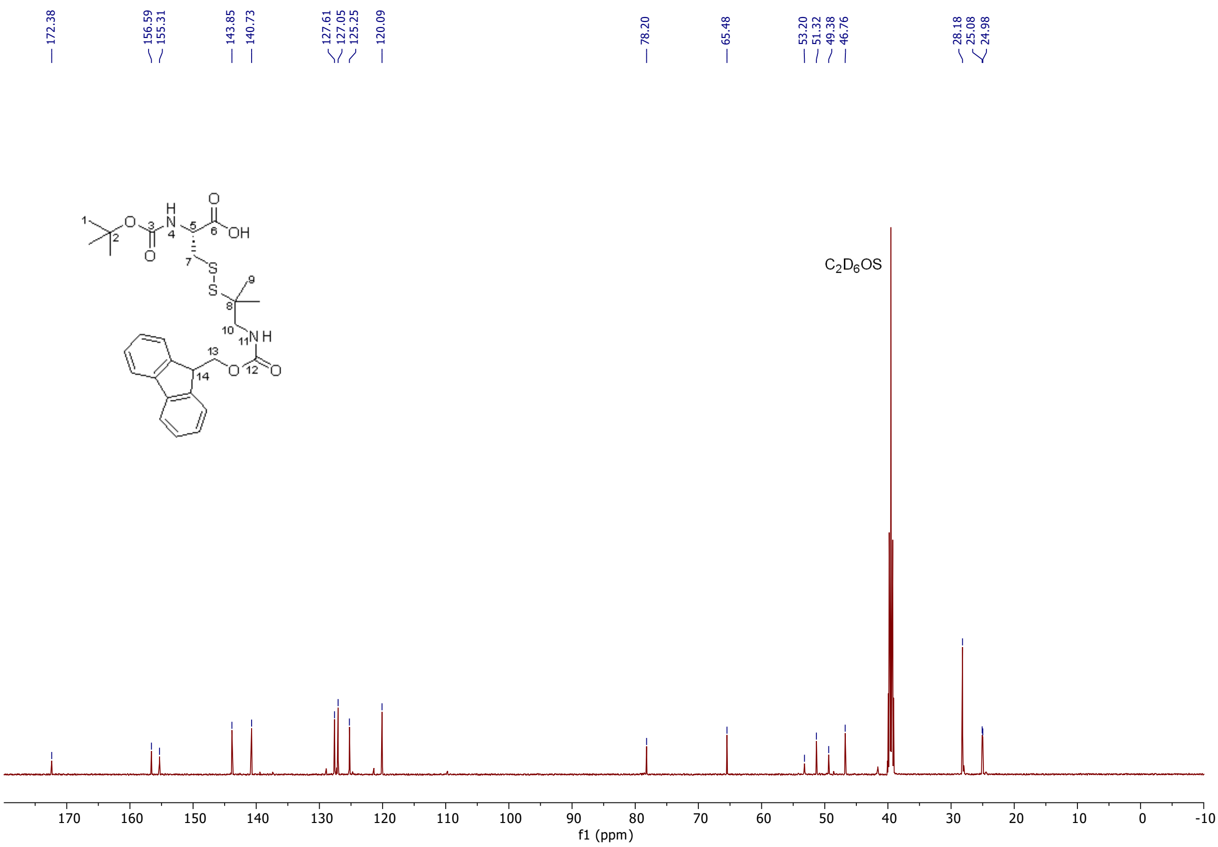

Supplementary figure S45: Copy of the ^{13}C NMR spectrum of **17** (151 MHz, DMSO-d_6).

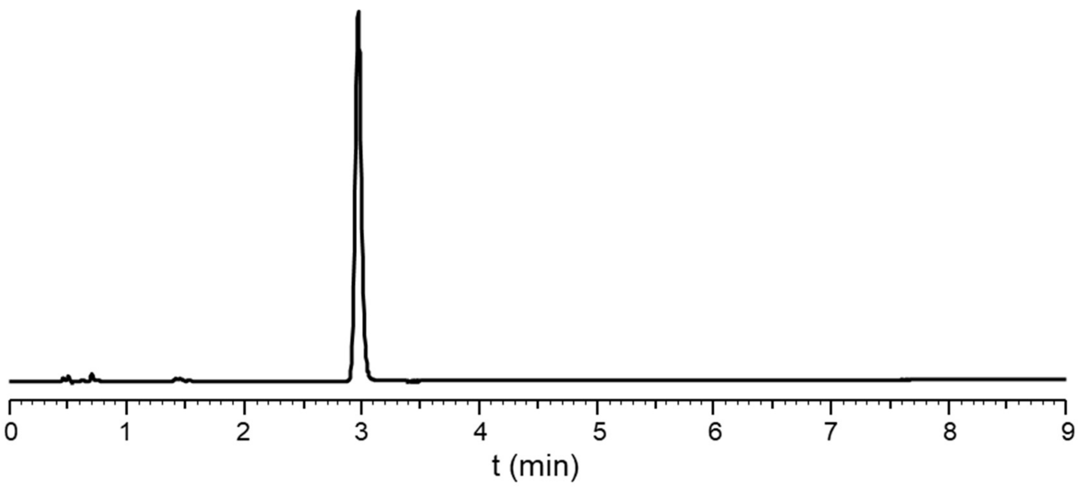

Supplementary figure S46: HPLC trace of purified **17** (λ = 254 nm).

11.2.2 [64]Gly-*N*-Hmb Cys(K_6-Ades)-SUMO-2[49-93]-OH (14a)

Sequence:

H-C(GABA-KKKKKK-Ades)ERQGLSXRQIRFRFD(Hmb)GQPINETDTPAQLEXEDEDTIDVFQQQTGG-OH

(X= Norleucine)

The peptide was synthesized on a preloaded Gly-trityl-Tentagel® resin (0.18 mmol/g) using protocol PS1. Hmb was incorporated according to protocol PS3 to prevent Asi formation during SPPS and NCL. Q88 and Q89 were subjected to double couplings. Boc-Cys(Fmoc-Ades)-OH (**17**) was incorporated using 3 equiv., with 2.95 equiv. of HATU and 6 equiv. of DIEA during 3 h. To allow UV titration of the amount of cleaved Fmoc and determine an SPPS elongation yield, peptidyl resin was treated according to protocol PS4 before the final deprotection. Prior to peptide deprotection and cleavage from the resin, Hmb was GABAylated using protocol PS6. Cleavage was performed for 4 h following protocol PS5. The crude cleaved peptide was solubilized in PBS/MeCN according to protocol PS8 to remove the GABA group prior to semi-preparative HPLC purification.

Elongation yield: 16%. Determined by the ratio between the quantity of fluorenylpiperidine released during final Fmoc deprotection and the quantity released during the Fmoc deprotection of the C-terminal Gly residue (UV titration at 301 nm, ε = 7800 $L.mol^{-1}.cm^{-1}$).

ESI-MS (*m/z*): [M] calcd. for $C_{277}H_{451}N_{80}O_{89}S_2$: 6389.2, found: 6388.4 (average mass, deconvoluted).

HPLC purification: [1 mg/mL] Nucleosil C18, gradient: 27-34 % B/A over 30 min affording a white solid after lyophilisation (22%).

HPLC analysis: t_R = 3.07 min (Chromolith, gradient: 25-35% B/A over 5 min).

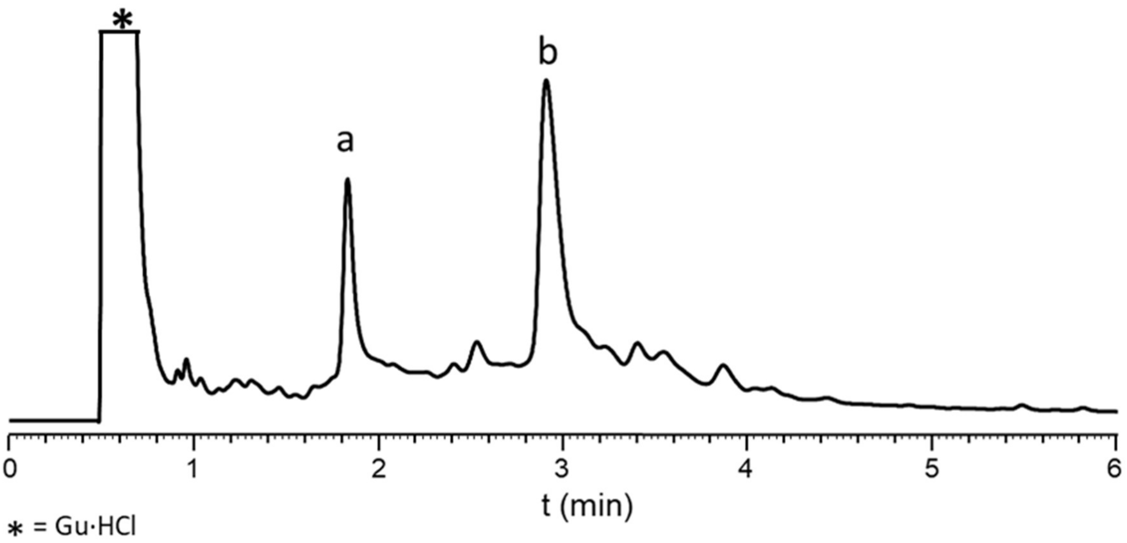

Peak (t_R (min))	[M] calcd.	[M] found	Attributed to
a (2.1 min)	2079.1	2078.6	Ac-SUMO-2[76-93]-OH
b (3.4 min)	6389.2	6388.1	**14a**

Supplementary figure S47: HPLC trace and MS analysis of crude **14a** (λ = 214 nm).

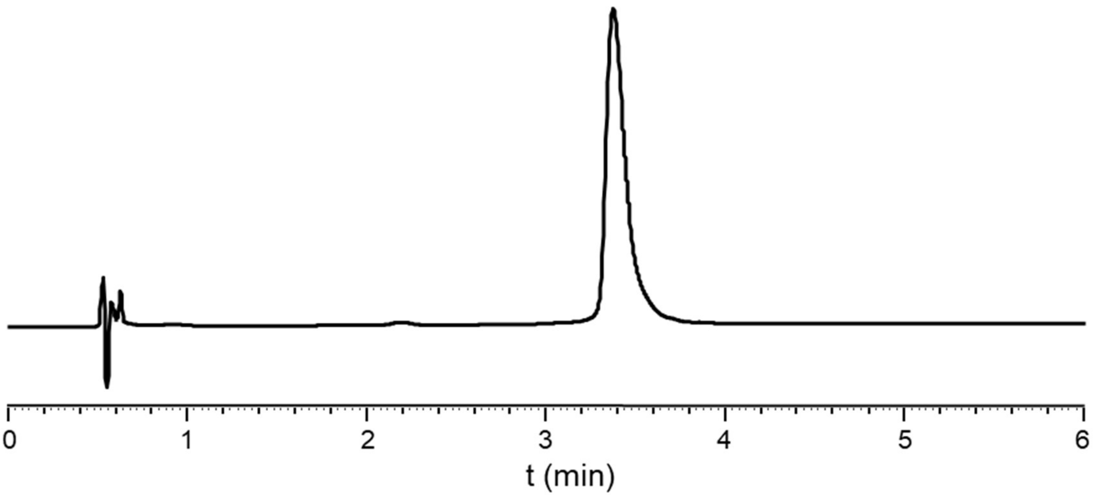

Supplementary figure S48: HPLC trace of purified **14a** (λ = 214 nm).

11.2.3 [64]Gly-*N*-Hmb Cys(K_6-Ades)-SUMO-2[49-92] propargylamide (20a)

Sequence:

H-C(GABA-KKKKKK-Ades)ERQGLSXRQIRFRFD(Hmb)GQPINETDTPAQLEXEDEDTIDVFQQQTG-NH-CH_2-C≡CH

(X= Norleucine)

BAL linker was loaded on Rink amide ChemMatrix® resin (1.25 equiv. BAL linker, 1.25 equiv. HATU, 5 equiv. DIEA in NMP for 2 h). Then the resin was treated with 9:9:2 DMF/MeOH/AcOH mixture (3 mL, 5 min). Next, the resin was washed and propargylamine (5 equiv.) and $NaBH_3CN$ (5 equiv.) were added and the mixture was stirred for 12 h. Then, [92]Gly was coupled manually (tenfold excess, 18h coupling) and the rest of the peptide was synthesized following protocol PS1. The peptide was synthesized using protocol PS1. Hmb was incorporated according to protocol PS3 to prevent Asi formation during SPPS and NCL. Q88 and Q89 were subjected to double couplings. Boc-Cys(Fmoc-Ades)-OH (**17**) was incorporated using 3 equiv., with 2.95 equiv. of HATU and 6 equiv. of DIEA during 3 h. To allow UV titration of the amount of cleaved Fmoc and determine an SPPS elongation yield, peptidyl resin was treated according to protocol PS4 before the final deprotection. Prior to peptide deprotection and cleavage from the resin, Hmb was GABAylated using protocol PS6. Cleavage was performed for 4 h following protocol PS5. The crude cleaved peptide was solubilized in PBS/MeCN according to protocol PS8 to remove the GABA group prior to semi-preparative HPLC purification.

Elongation yield: 14 %. Determined by the ratio between the quantity of fluorenylpiperidine released during final Fmoc deprotection and the quantity released during the Fmoc deprotection of the C-terminal Gly residue (UV titration at 302 nm, ε = 7800 $L.mol^{-1}.cm^{-1}$).

ESI-MS (*m/z*): [M] calcd. for $C_{274}H_{444}N_{79}O_{86}S_2$: 6369.2, found: 6367.9 (average mass, deconvoluted).

HPLC analysis: t_R = 3.37 (Chromolith, gradient: 25-35% B/A over 5 min).

HPLC purification: [1 mg/mL] Nucleosil C18, gradient: 27-34% B/A over 30 min affording a white solid after lyophilisation (24 %).

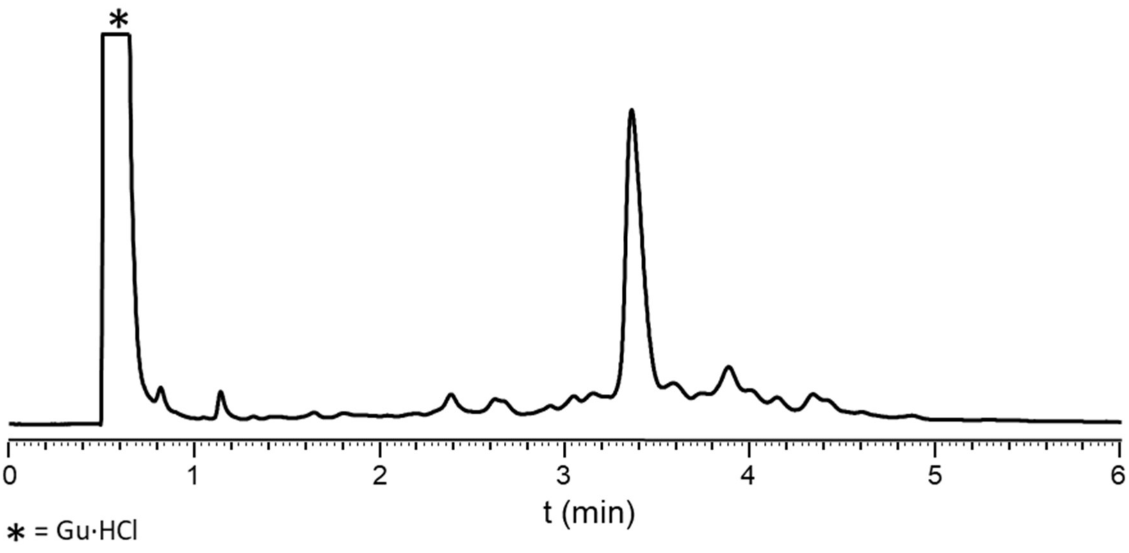

Supplementary figure S49: HPLC trace of crude **20a** (λ = 214 nm).

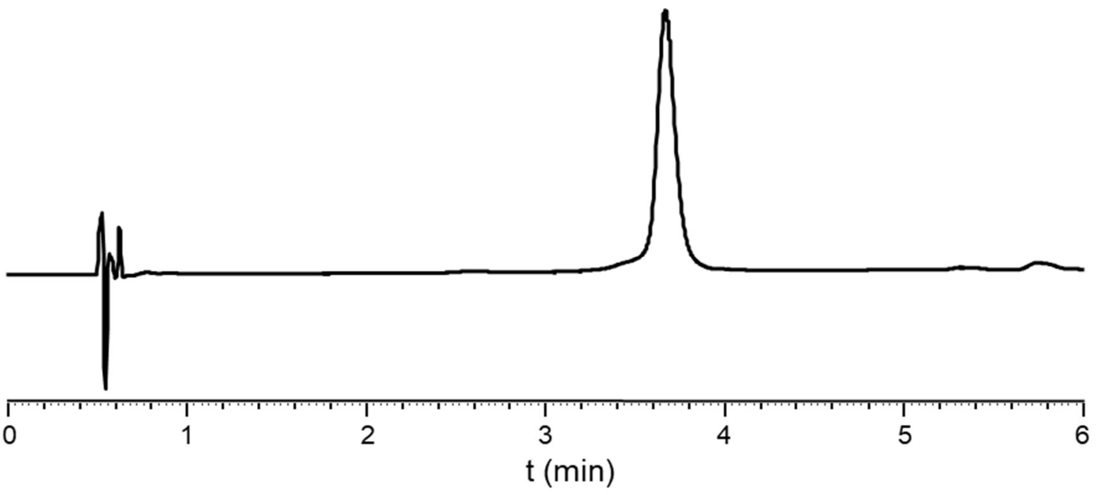

Supplementary figure S50: HPLC trace of purified **20a** (λ = 214 nm).

12 Native chemical ligation

12.1 Aspartimide-free synthesis of SUMO-2 (3)

Crypto-thioester **13a** (6.5 mg, 0.880 µmol, 1.1 equiv.) and cysteinyl peptide **14a** (6.02mg, 0.8 µmol, 2 mM final concentration) were dissolved in NCL buffer (400 µL, protocol PS9) and the resulting yellow solution was gently stirred under inert atmosphere at 37°C for 20 h. The mixture was then diluted with 4 ml of a water/MeCN/TFA 8:2:0.1 mixture, then desalted by dialysis for 16 h against the same solvent mixture and lyophylized. The ligated product was treated according to the PS10 protocol to eliminate the Hmb protective groups. The crude mixture was purified by semi-preparative HPLC.

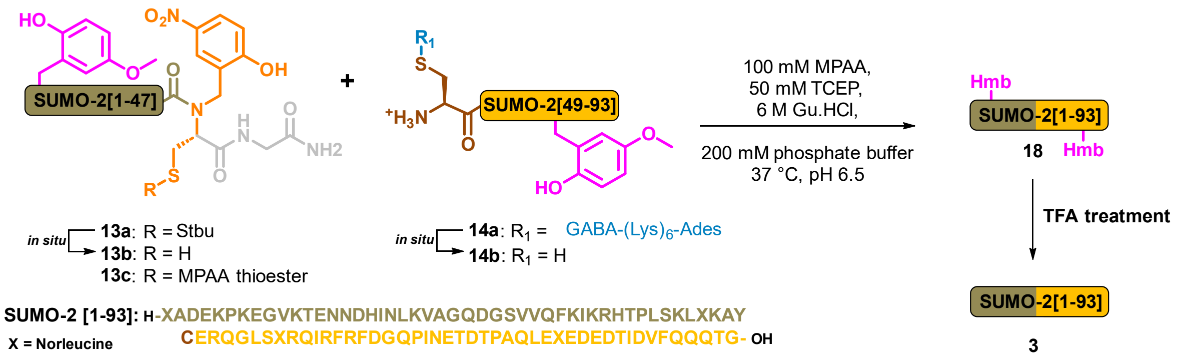

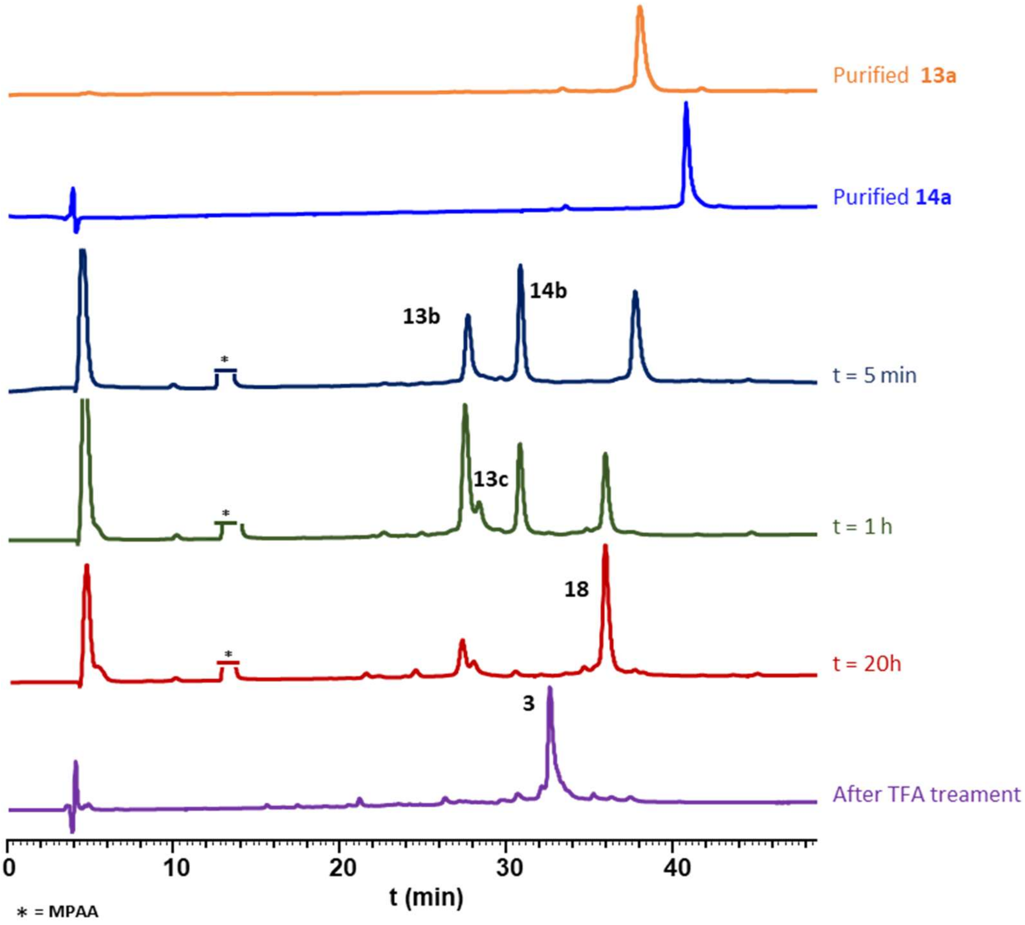

Supplementary figure S51: HPLC monitoring (Nucleosil C18, gradient: 15-40% B/A over 50 min) of ligation between compounds **13a** and **14a**.

ESI-MS (*m/z*): [M] calcd. for $C_{459}H_{739}N_{133}O_{149}S$: 10536.6, found: 10535.4 (average mass, deconvoluted).

ESI-HRMS (*m/z*): [M] calcd. for $C_{459}H_{739}N_{133}O_{149}S$: 10530.4059, found: 10530.4060 (deconvoluted)

HPLC analysis: t_R = 3.39 min (Chromolith, gradient: 20-50% B/A over 5 min).

HPLC purification: [2 mg/mL] Nucleosil C18, gradient: 26-38% B/A over 40 min affording a white solid after lyophilisation (overall yield: 21%).

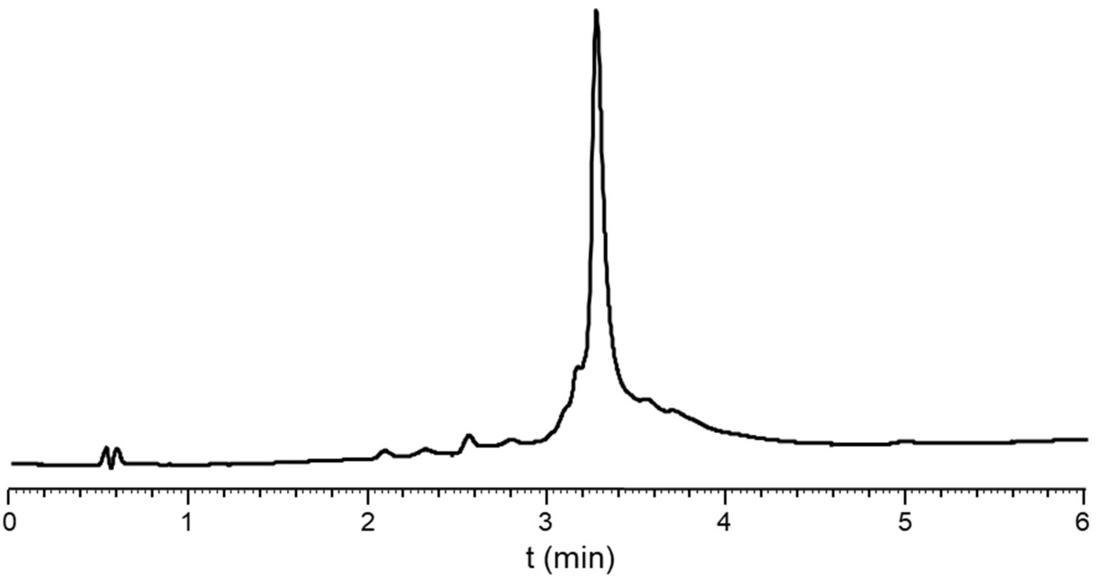

Supplementary figure S52: HPLC trace of crude **3** (λ = 214 nm).

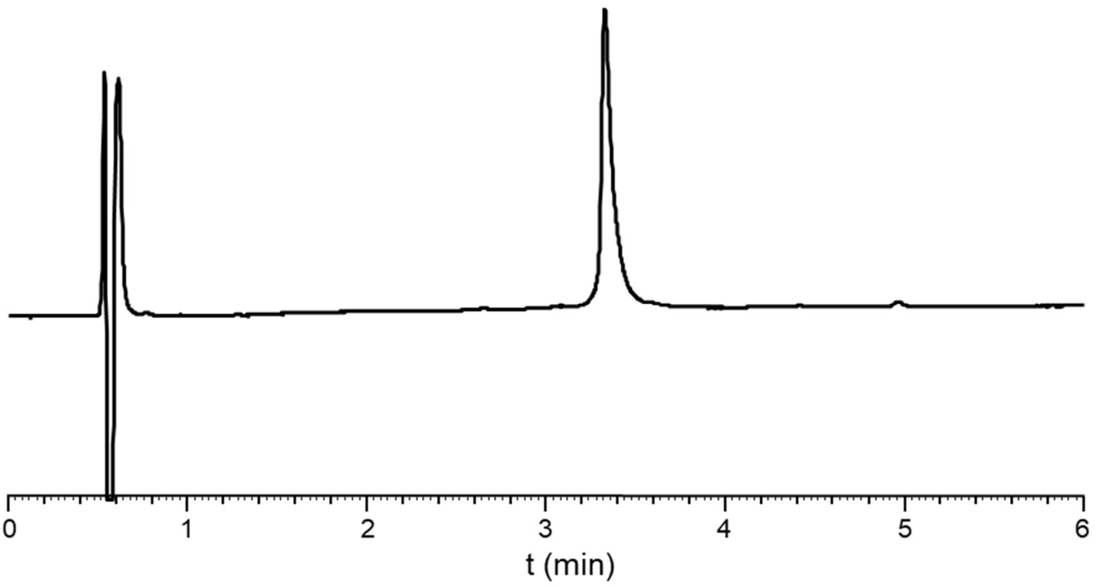

Supplementary figure S53: HPLC trace of purified **3** (λ = 214 nm).

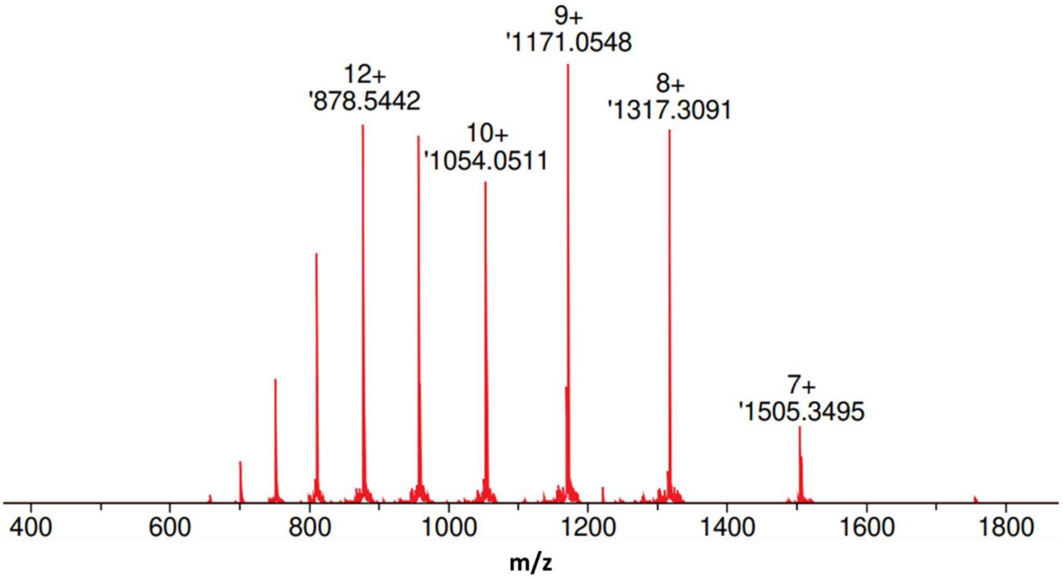

Supplementary figure S54: High resolution mass spectrum of purified SUMO-2[1-93] (**3**).

a. Previous work[4]: synthesis of SUMO-2 without *N*-Hmb protection

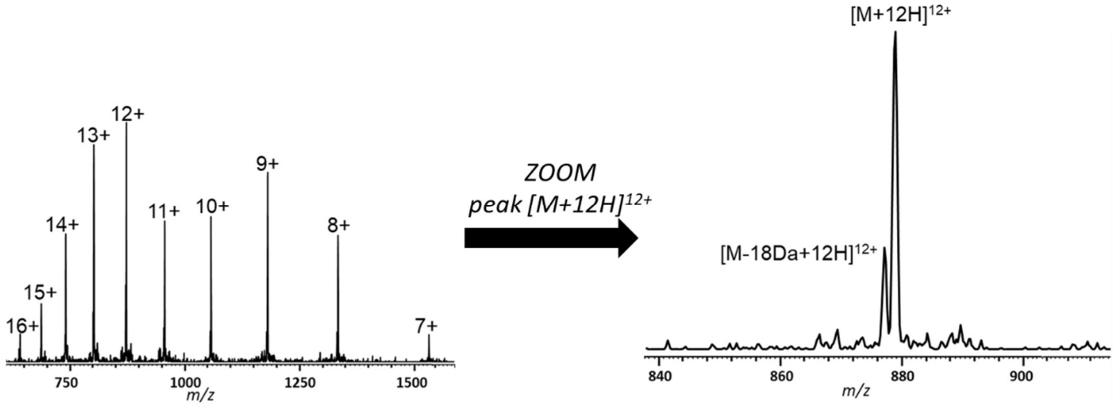

b. This work: synthesis of SUMO-2 using the Hmb strategy

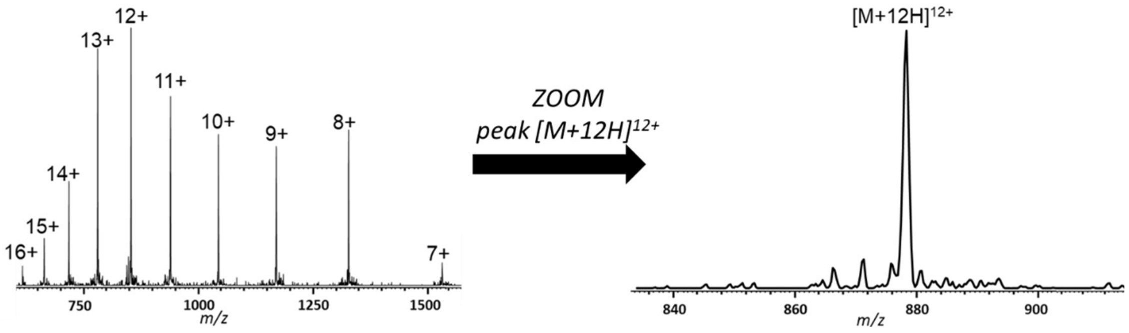

Supplementary figure S55: Comparison of mass spectra of purified SUMO-2 protein (**3**) synthesized with or without Hmb protecting group.

12.2 Synthesis of a SUMO-2 protein equipped with a C-terminal alkyne moiety

Crypto-thioester **14a** (6.5 mg, 0.88 µmol, 1.1 equiv.) and cysteinyl peptide **20a** (6.01 mg, 0.80 µmol, 2 mM final concentration) were dissolved in NCL buffer (400 µL, protocol PS8) and the resulting yellow solution was gently stirred under inert atmosphere at 37 °C for 20 h, then quenched by dilution into 4 ml of a 6 M Gn·HCl solution containing 1% *v*/*v* AcOH. Semi preparative HPLC purification afforded *bis*-Hmb SUMO-2 propargylamide **19**. Hmb deprotection following protocol PS10 gave SUMO-2 propargylamide **S11** which pas purified by semi preparative HPLC.

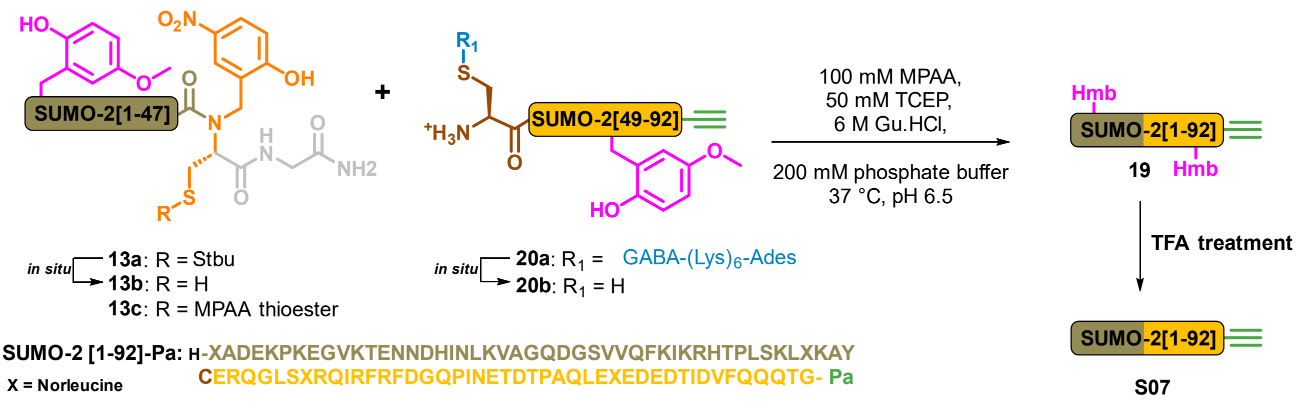

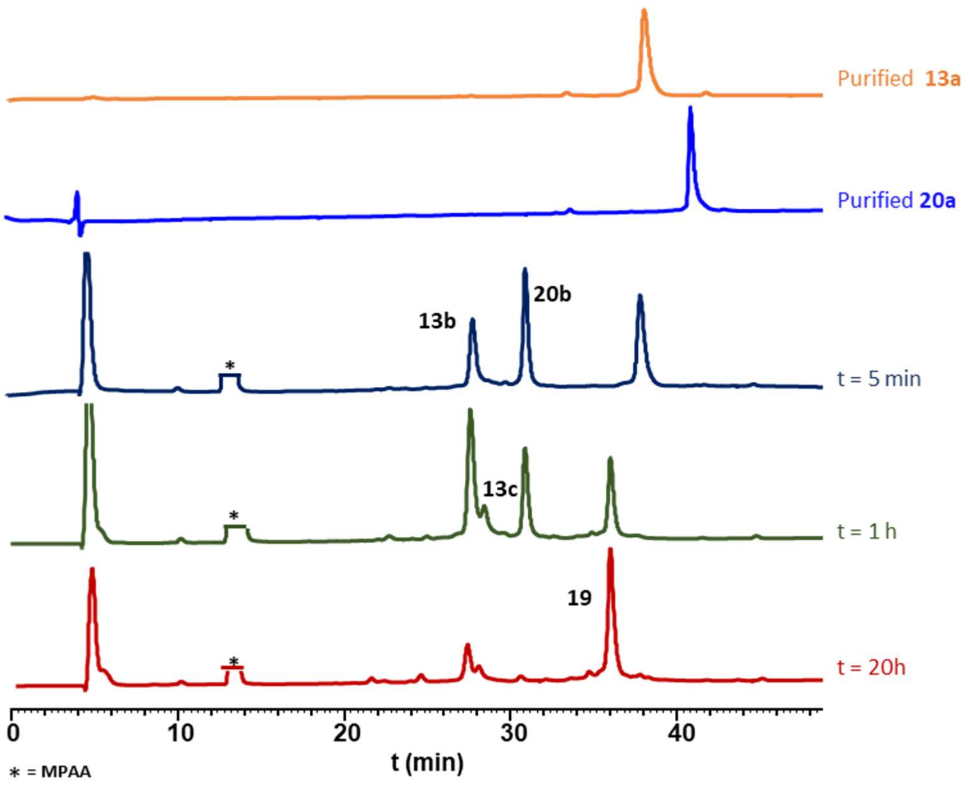

Supplementary figure S56: HPLC monitoring (Nucleosil C18, gradient: 15-40% B/A over 50 min) of ligation between compounds **13a** and **20a**.

Bis-Hmb SUMO-2 propargylamide (**19**)

ESI-MS (*m/z*): [M] calcd. for $C_{476}H_{755}N_{133}O_{151}S$: 10789.0, found: 10787.7 (average mass, deconvoluted).

HPLC analysis: t_R = 3.60 min (Chromolith, gradient: 25-45% B/A over 5 min).

HPLC purification: [2 mg/mL] Nucleosil C18, gradient: 26-38% B/A over 40 min affording a white solid after lyophilisation (52 % yield).

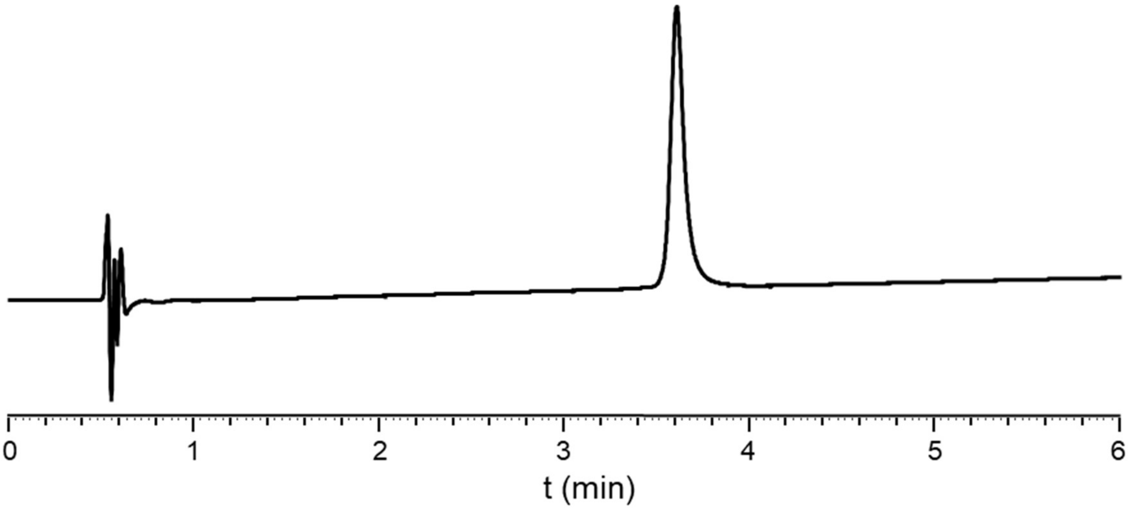

Supplementary figure S57: HPLC trace of purified **19** (λ = 214 nm).

SUMO-2 propargylamide (**S07**)

ESI-MS (*m/z*): [M] calcd. for $C_{459}H_{739}N_{133}O_{149}S$: 10516.7, found: 10515.4 (average mass, deconvoluted).

HPLC analysis: t_R = 2.61 min (Chromolith, gradient: 20-50% B/A over 5 min).

HPLC purification: [2 mg/mL] Nucleosil C18, gradiet: 26-38% B/A over 40 min affording a white solid after lyophilisation (30% yield).

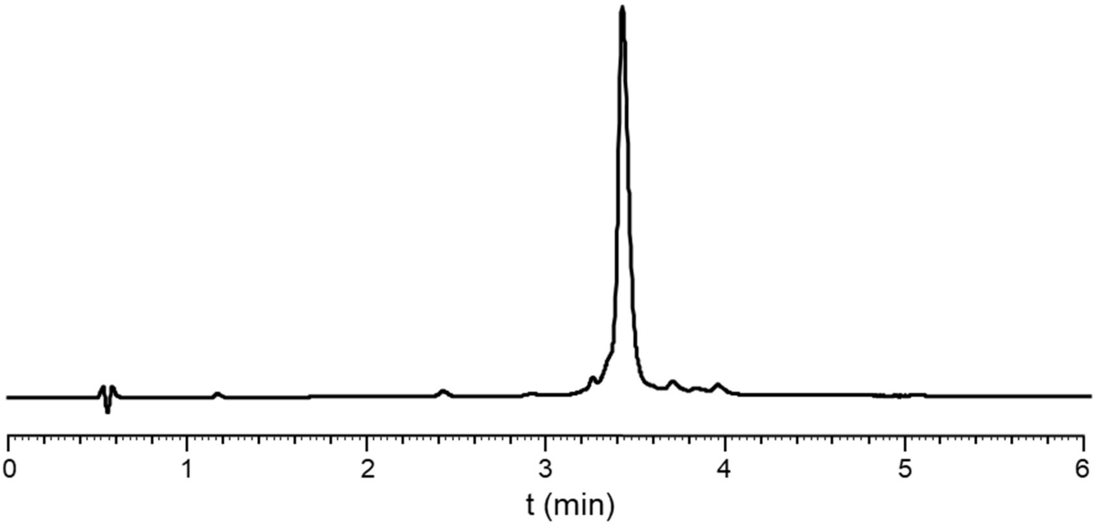

Supplementary figure S58: HPLC trace of crude **S07** (λ = 214 nm).

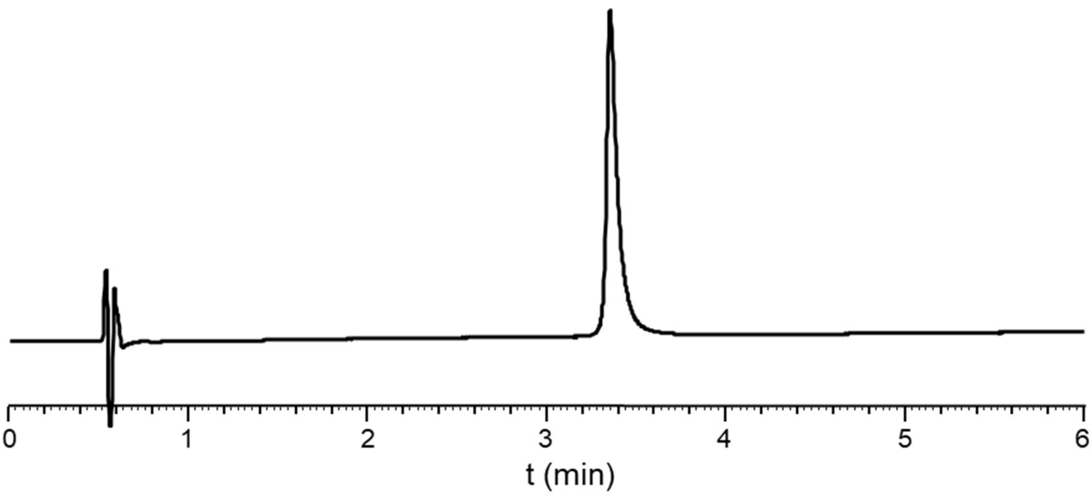

Supplementary figure S59: HPLC trace of purified **S07** (λ = 214 nm).

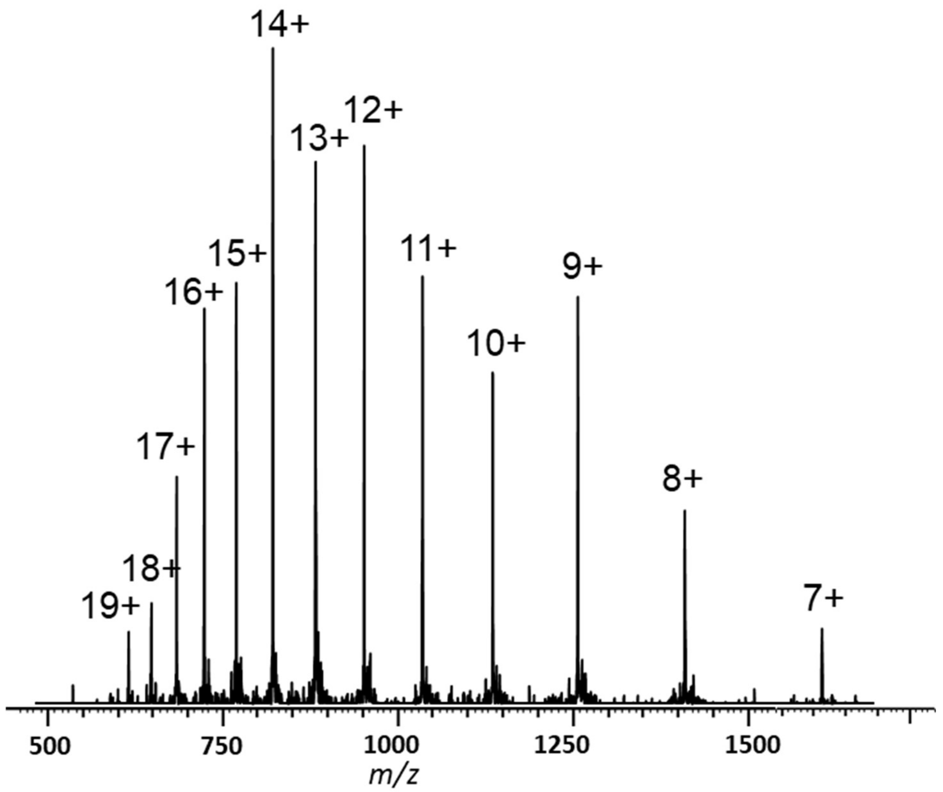

Supplementary figure S60: Mass spectrum of purified SUMO-2[1-92]-Pa **S07**.

13 Chemical SUMOylation

13.1 Synthesis of azido model peptide (21)

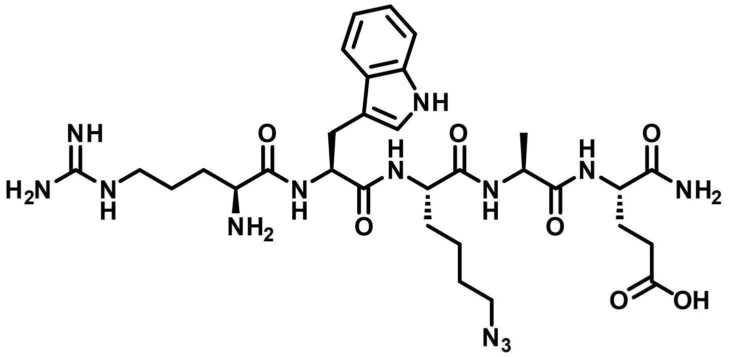

The peptide was synthesized on a Rink amide MBHA polystyrene resin (0.68 mmol/g) following protocol PS1, using Fmoc-Lys(N_3)-OH as building block to introduce the azido moiety. Cleavage was performed for 2 h following protocol PS5. The crude mixture was purified by semi-preparative HPLC.

ESI-MS (*m/z*): [M+H] calcd. for $C_{31}H_{48}N_{13}O_7^+$: 714.4, found: 904.4 (monoisotopic mass).

HPLC analysis: t_R = 3.30 min (Chromolith, gradient: 5-50% B/A over 5 min).

HPLC purification: [5 mg/mL] Nucleosil C18, gradient: 15-45% B/A over 60 min.

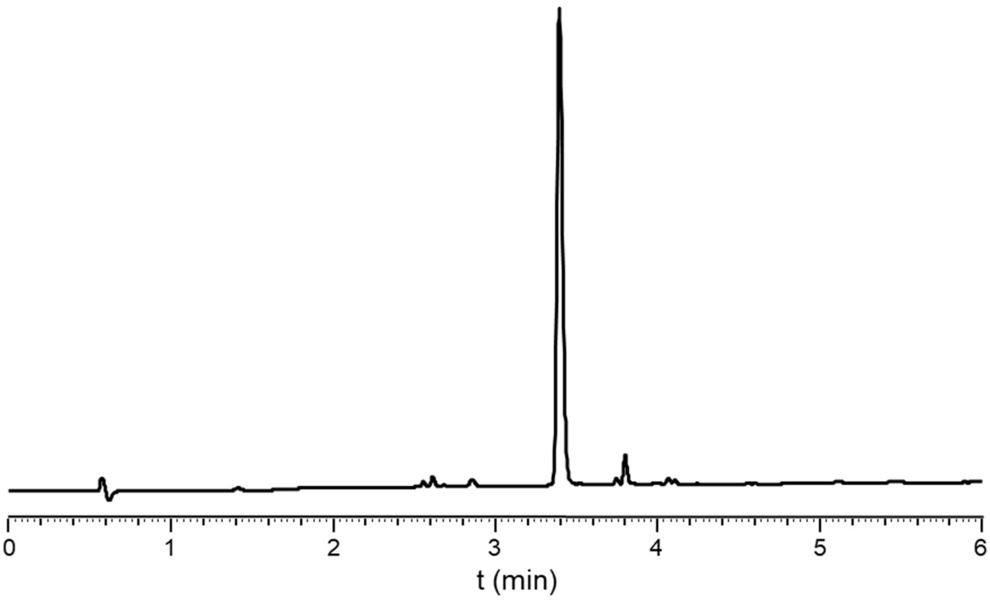

Supplementary figure S61: HPLC trace of crude **21** (λ = 214 nm).

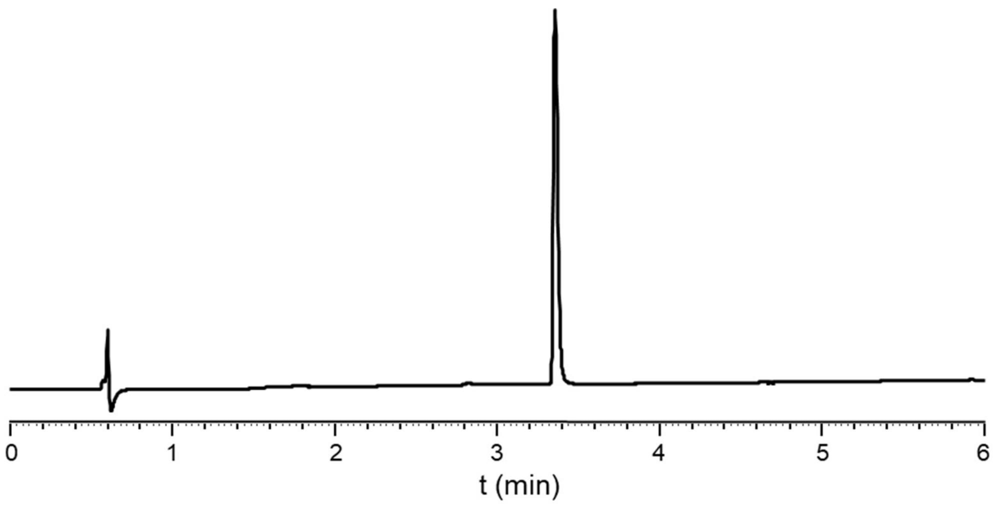

Supplementary figure S60: HPLC trace of purified **21** (λ = 214 nm).

13.2 CuAAC reaction between 19 and 21

To a solution of **19** (1.62 mg, 150 nmol, 1 mM final concentration) and peptide **21** (2 equiv.) in 75 µL of deoxygenated HFIP was added 50 µL of deoxygenated 200 mM HEPES buffer pH = 8.0 under argon. To prepare a fresh solution of Cu(I)·THPTA in HEPES buffer, 12.5 µL mixture containing $CuSO_4$ pentahydrate (10 equiv.) and THPTA (30 equiv.) was added to 12.5 µL of sodium ascorbate (42.5 equiv.). The Cu(I) solution was added to the ligation mixture and the reaction was stirred during 5 h at 25 °C. The reaction was quenched by adding 200 µL of a disodium EDTA solution (50 equiv.), then the mixture was diluted with 1.1 ml of a 6 M Gn·HCl solution. Semi preparative HPLC purification afforded *bis*-Hmb conjugate **22**. Hmb deprotection following protocol PS10 gave SUMOylated peptide mimic **23** which pas purified by semi preparative HPLC.

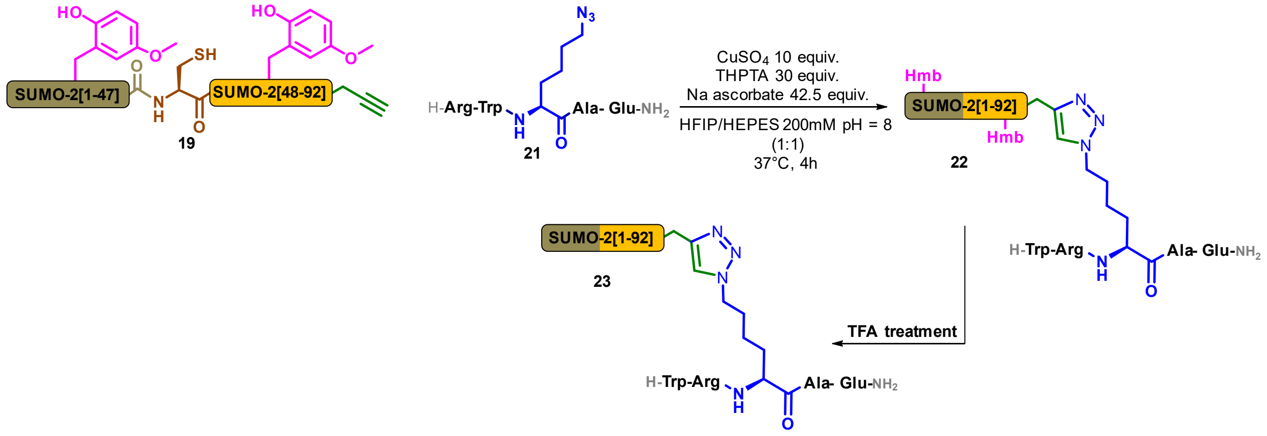

Bis-Hmb SUMOylated peptide mimic (**22**)

ESI-MS (*m/z*): [M] calcd. for $C_{507}H_{804}N_{146}O_{158}S$: 11504.8, found: 11500.7 (average mass, deconvoluted).

HPLC analysis: t_R = 3.72 min (Chromolith, gradient: 25-45% B/A over 5 min).

HPLC purification: [2 mg/mL] Chromolith, gradient: 20-50% B/A over 8 min affording a white solid after lyophilisation (75 % yield).

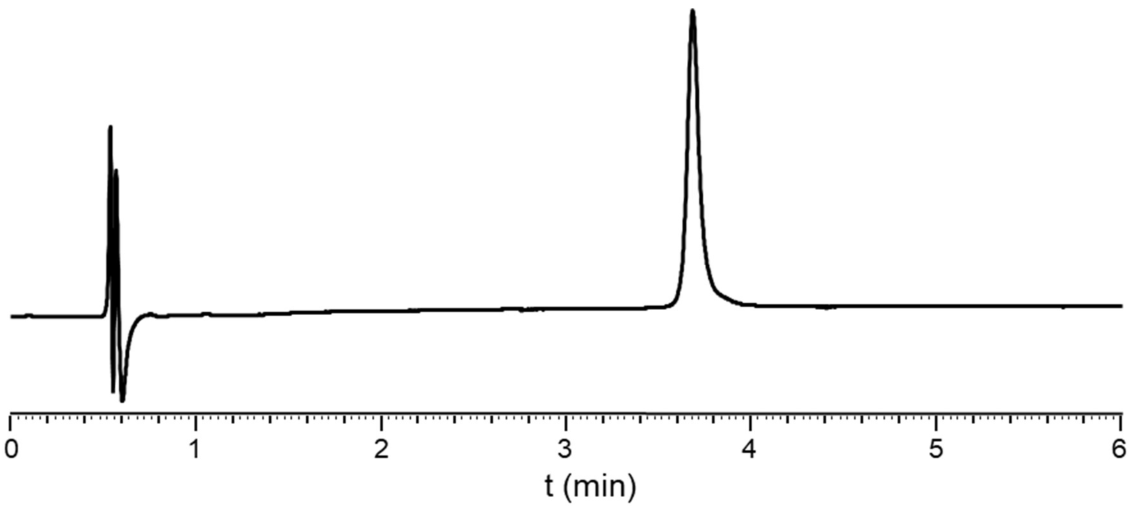

Supplementary figure S61: HPLC trace of purified **22** (λ = 214 nm).

SUMOylated peptide mimic **23**

ESI-MS (*m/z*): [M] calcd. for $C_{507}H_{802}N_{146}O_{158}S$: 11226.8, found: 11228.3 (average mass, deconvoluted).

HPLC analysis: t_R = 3.61 min (Chromolith, gradient: 25-45% B/A over 5 min).

HPLC purification: [2 mg/mL] Nucleosil C18, gradient: 26-38% B/A over 40 min affording a white solid after lyophilisation (35% yield).

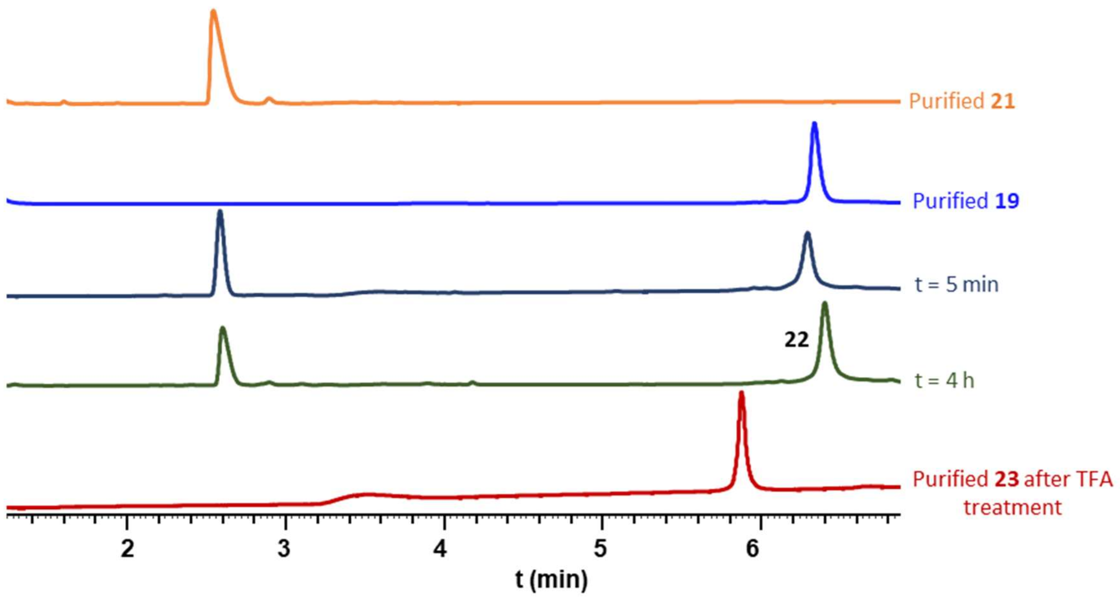

Supplementary figure S62: HPLC monitoring (Nucleosil C18, gradient: 15-40% B/A over 50 min) of CuAAC between compounds **19** and **21** followed by TFA treatment and HPLC purification.

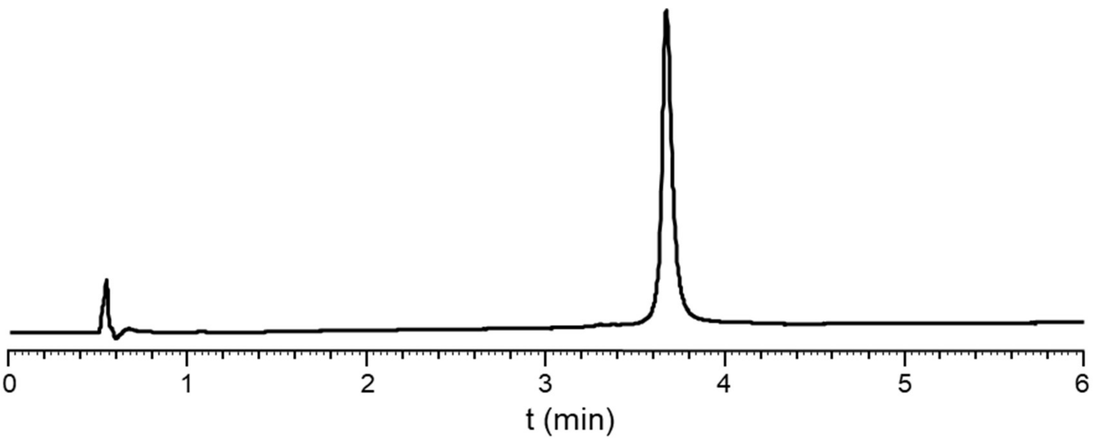

Supplementary figure S63: HPLC trace of purified **23** (λ = 214 nm).

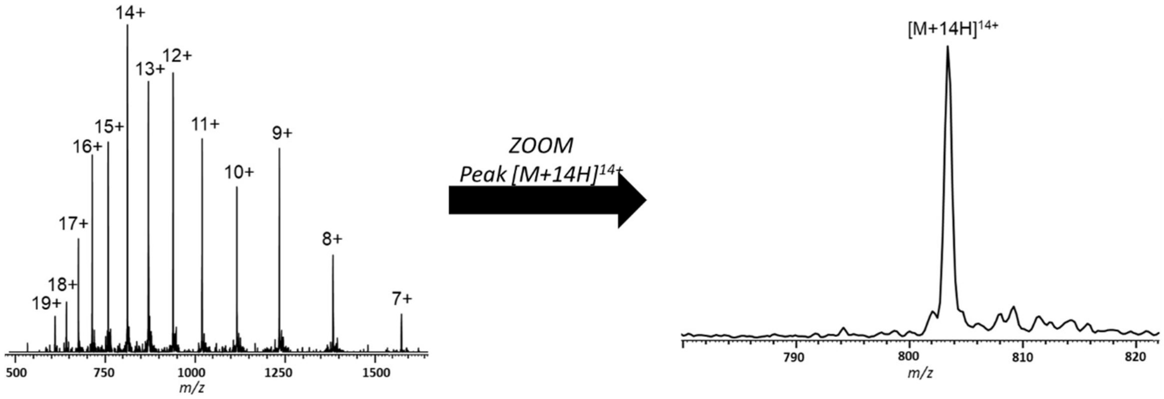

Supplementary figure S64: Mass spectrum of purified SUMOylated peptide mimic **23**.

14 Bibliography

1 C. N. Pace, F. Vajdos, L. Fee, G. Grimsley and T. Gray, *Protein Sci.*, 1995, **4**, 2411–2423.
2 D. Lelièvre, V. P. Terrier, A. F. Delmas and V. Aucagne, *Org. Lett.*, 2016, **18**, 920–923.
3 M. M. Garcia-Mira and J. M. Sanchez-Ruiz, *Biophys. J.*, 2001, **81**, 3489–3502.
4 S. A. Abboud, E. hadji Cisse, M. Doudeau, H. Bénédetti and V. Aucagne, *Chem. Sci.*, 2021, **12**, 3194–3201.
5 A.-B. M. Abdel-Aal, R. Raz, G. Papageorgiou and J. Offer, in *Peptide Synthesis*, eds. W. M. Hussein, M. Skwarczynski and I. Toth, Springer US, New York, NY, 2020, vol. 2103, pp. 225–237.
